# SPA70 is a potent antagonist of human pregnane X receptor

**DOI:** 10.1038/s41467-017-00780-5

**Published:** 2017-09-29

**Authors:** Wenwei Lin, Yue-Ming Wang, Sergio C. Chai, Lili Lv, Jie Zheng, Jing Wu, Qijun Zhang, Yong-Dong Wang, Patrick R. Griffin, Taosheng Chen

**Affiliations:** 1 Department of Chemical Biology and Therapeutics, St. Jude Children’s Research Hospital, Memphis, TN 38105-3678 USA; 2 Structure Biology, Shanghai Medicilon Inc., Shanghai, 201299 China; 3 Department of Molecular Medicine, The Scripps Research Institute, Scripps Florida, Jupiter, FL 33458 USA; 4 Department of Computational Biology, St. Jude Children’s Research Hospital, Memphis, TN 38105-3678 USA

## Abstract

Many drugs bind to and activate human pregnane X receptor (hPXR) to upregulate drug-metabolizing enzymes, resulting in decreased drug efficacy and increased resistance. This suggests that hPXR antagonists have therapeutic value. Here we report that SPA70 is a potent and selective hPXR antagonist. SPA70 inhibits hPXR in human hepatocytes and humanized mouse models and enhances the chemosensitivity of cancer cells, consistent with the role of hPXR in drug resistance. Unexpectedly, SJB7, a close analog of SPA70, is an hPXR agonist. X-ray crystallography reveals that SJB7 resides in the ligand-binding domain (LBD) of hPXR, interacting with the AF-2 helix to stabilize the LBD for coactivator binding. Differential hydrogen/deuterium exchange analysis demonstrates that SPA70 and SJB7 interact with the hPXR LBD. Docking studies suggest that the lack of the para-methoxy group in SPA70 compromises its interaction with the AF-2, thus explaining its antagonism. SPA70 is an hPXR antagonist and promising therapeutic tool.

## Abstract (web summary)

The xenobiotic-activated human pregnane X receptor (hPXR) regulates drug metabolism. Here the authors develop hPXR modulators, which are of potential therapeutic interest and functionally and structurally characterize the antagonist SPA70 and the structurally related agonist SJB7.

## Introduction

The xenobiotic receptor pregnane X receptor (PXR) is an orphan member of the nuclear receptor superfamily^1–4^. The activity of human PXR (hPXR) can be induced by many structurally diverse chemicals, leading to the transcriptional upregulation of drug-metabolizing enzymes (e.g., cytochrome P450 3A4 [CYP3A4]) and transporters (e.g., multidrug resistance protein 1 [MDR1]) to enhance the metabolism and excretion of xenobiotics, such as therapeutic agents, and endobiotics, such as steroid hormones, bile acids and glucose^5, 6^. Such upregulation of hPXR has broad implications not only for the therapeutic and toxic effects of drugs but also for the development of diseases such as diabetes and cancers^7–11^. PXR forms heterodimers with retinoid X receptor (RXR) to bind to the promoters of its target genes. The transcriptional activity of PXR is enhanced by co-activators, such as steroid receptor coactivator 1 (SRC-1) and transcriptional mediator/intermediary factor 2 (TIF2), and repressed by co-repressors, such as nuclear receptor corepressor (NCoR) and silencing mediator for retinoid or thyroid hormone receptors (SMRT)^12^. The activation function 2 (AF2) domain at the C-terminus of PXR mediates interaction with these cofactors^4^. Because of the species-specificity of PXR ligands, a mouse model in which mouse PXR (mPXR) is replaced with hPXR (a ‘humanized’ mouse model) enables us to examine the in vivo function of hPXR^13, 14^.

Having been first characterized as a xenobiotic-activated receptor that regulates drug metabolism and excretion, PXR is now known to be a signaling molecule (that can also serve as a ‘scaffold’) that interacts with other signaling molecules^15^. Because of the possible nongenomic scaffolding function of PXR, the phenotypes of PXR knockout mice (i.e., mice in which there is a permanent loss of PXR protein function) may differ from those of pharmacologically modulated mice (i.e., mice in which there is only a temporary change in protein function). Therefore, a PXR antagonist is a critical pharmacological tool for understanding the regulation and function of PXR. In addition, there is growing evidence that PXR mediates adverse metabolic phenotypes of both xenobiotics (e.g., acetaminophen and rifampicin/isoniazid toxicity) and endobiotics (e.g., hepatic steatosis)^16–18^. Furthermore, PXR activation induces resistance to chemotherapeutic agents for cancer^18, 19^. Therefore, PXR antagonists might have significant therapeutic value. However, although many PXR agonists have been characterized, with multiple co-crystal structures having been reported^20–23^, only a few structurally unrelated hPXR antagonists have been described, and they are known to target other biologic processes and are not appropriate pharmacological tools for investigating the specific regulation of hPXR^18^. Sulforaphane and ketoconazole are the most comprehensively studied of these reported hPXR antagonists, but they are not effective in vivo^24, 25^. Although the mode of action of sulforaphane remains unclear, toxicity might contribute to the lack of in vivo efficacy of ketoconazole as an effective hPXR antagonist^26^. There is, therefore, an urgent need to systematically develop a series of potent, more specific, less toxic and structurally related hPXR antagonists that are active in vivo and can be used for structure-function studies and, potentially, in clinical applications. We report the characterization of SPA70 as a potent, nontoxic, highly selective and cellularly active hPXR antagonist, along with the results of our initial investigation of the effect of antagonizing hPXR in humanized mouse and cancer cell models. Furthermore, the design and structural and functional characterization of SJB7, a close analog of SPA70 that we unexpectedly found to act as a potent hPXR agonist, provide insights into the structure-activity relationship (SAR) of hPXR-ligand interactions. SPA70 and its analogs represent a useful set of hPXR modulators that will facilitate further investigation of the structural and functional regulation of hPXR.

## Results

### Characterization of SPA70 as an hPXR antagonist

**Fig. 1:**
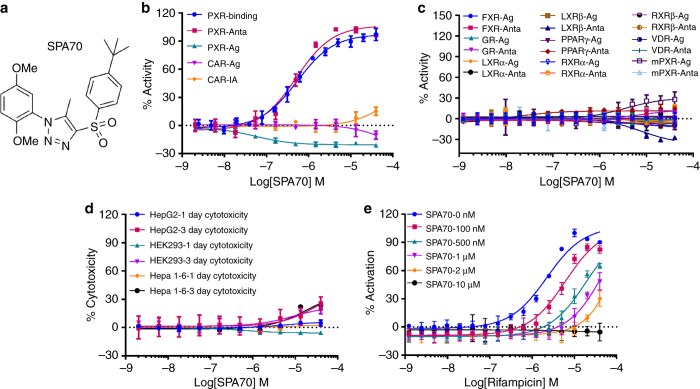
SPA70 is a specific hPXR antagonist. **a** Chemical structure of SPA70. **b**–**d** Dose–response curves of SPA70 in various assays. **b** PXR-binding: hPXR TR-FRET-binding assay (T0901317 IC_50_ = 96.1 nM); PXR-Anta and PXR-Ag: hPXR transactivation assay using the HepG2 stable cell line in antagonistic and agonistic modes (rifampicin EC_50_ = 1.18 µM); CAR-Ag and CAR-IA: hCAR transactivation assay in agonistic and inverse agonistic modes (CITCO EC_50_ = 60.8 nM in agonistic mode; PK11195 IC_50_ = 247.2 nM in inverse agonistic mode); **c** FXR-Ag and FXR-Anta, GR-Ag and GR-Anta, LXRα-Ag and LXRα-Anta, LXRβ-Ag and LXRβ-Anta, PPARγ-Ag and PPARγ-Anta, RXRα-Ag and RXRα-Anta, RXRβ-Ag and RXRβ-Anta, and VDR-Ag and VDR-Anta: GeneBLAzer NR-UAS-bla HEK 293 T assays for FXR, GR, LXRα, LXRβ, PPARγ, RXRα, RXRβ, and VDR in agonistic (Ag) and antagonistic (Anta) modes; mPXR-Ag and mPXR-Anta: mPXR transactivation assay in agonistic and antagonistic modes. % Activity refers to the % inhibition or % activation as described for each assay (EC_50_ = 77.1, 2.2, 21.3, 50.4, 5.0, 13.3, 7.6, 0.4 and 293.8 nM for GW4604/FXR, dexamethasone/GR, T0901317/LXRα, T0901317/LXRβ, rosiglitazone/PPARγ, 9-*cis*-retinoic acid/RXRα, 9-*cis*-retinoic acid/RXRβ, 1α, 25-dihydroxyvitamin D3/VDR and pregnenolone 16α-carbonitrile [PCN]/mPXR, respectively). **d** One- and 3-day cytotoxicity assays in HepG2, HEK293, and Hepa 1-6 cells. **e** SPA70 inhibits rifampicin-induced hPXR activation in a dose-responsive manner. Each data point represents the mean ± S.E.M. from quadruplicate measurements. Representative results from at least triplicated experiments are shown

The unique ligand-binding pocket of hPXR enables it to bind to structurally diverse molecules of different sizes, making it a challenging task to design antagonists of hPXR by using a structure-based approach. Therefore, we sought a cell-based, unbiased, high-throughput chemical biology approach by which to identify antagonists of hPXR. By using the HepG2 human liver carcinoma cell line stably expressing hPXR and a luciferase reporter under the control of the promoter of *CYP3A4* ^27^, a transcriptional target of hPXR, we screened a library of 160,000 chemically diverse compounds in the presence of rifampicin, an hPXR agonist and identified a number of hPXR antagonists, including SJ000076745-1, the most potent hPXR antagonist identified from the screen (Lin et al., in submission), which we call SPA70 (specific PXR antagonist #70, also known as LC-1) (Supplementary Table 1). SPA70 has a 1-substituted-phenyl-4-substituted-phenylsulfonyl-5-methyl-1*H*-1,2,3-triazole scaffold (Fig. 1a) with an IC_50_ of 510 nM in the cell-based hPXR antagonistic assay (Fig. 1b and Supplementary Table 1). In a cell-free competitive hPXR TR-FRET-binding assay, SPA70 demonstrated an IC_50_ of 540 nM (*K*_i_ = 390 nM) (Fig. 1b and Supplementary Table 1). SPA70 was highly selective for hPXR, as compared to 10 other nuclear receptors that we tested (constitutive androstane receptor [CAR], farnesoid X receptor (FXR), glucocorticoid receptor [GR], liver X receptor α [LXRα] and LXRβ, peroxisome proliferator-activated receptor γ [PPARγ], RXRα and RXRβ, vitamin D receptor [VDR], and mPXR): SPA70 very weakly inhibited the basal activity of hPXR and CAR (Fig. 1b); it functioned as a weak agonist of mPXR and marginally increased the agonistic activity of T0901317 on LXRβ (only at concentrations higher than 5 µM) (Fig. 1c). SPA70 showed marginal cytotoxicity in the cell models used, i.e., HepG2, HEK293 and Hepa 1-6 cells (Fig. 1d). To obtain additional information on the selectivity of SPA70 for hPXR, we performed a kinase inhibition profiling assay (Supplementary Data 1). Among the 384 kinases tested, only three were inhibited by more than 50% by 10 µM SPA70, and only KIT and colony-stimulating factor 1 receptor (CSF1R) were confirmed to be moderately inhibited by SPA70, with *K*_d_ values of 2.5 and 4 µM, respectively. Cyclin-dependent kinase 2 (CDK2), cyclin-dependent kinase 5 (CDK5), protein kinase A (PKA or PKAC), glycogen synthase kinase 3 (GSK3) and dual-specificity tyrosine phosphorylation-regulated kinase 2 (DYRK2) have been reported to possibly affect the function of PXR^4, 12^, but they are not inhibited by SPA70 (Supplementary Data 1).

SPA70 antagonized the agonistic effect of rifampicin in a dose-dependent manner (Fig. 1e). In the absence of SPA70, the EC_50_ of rifampicin was 1.2 μM. Increasing the concentrations of SPA70 decreased the activity of rifampicin, and an SPA70 concentration of 10 μM completely blocked the activity of rifampicin at all concentrations of that drug tested (Fig. 1e). We designed and evaluated 15 analogs of SPA70 (Supplementary Table 1). Among the SPA70 analogs, SJC2 also exhibited strong hPXR antagonistic activity. However, SJC2 also moderately antagonized PPARγ and reduced its basal activity (Supplementary Fig. 1). Therefore, we focused on SPA70 in our subsequent analysis.

**Fig. 2:**
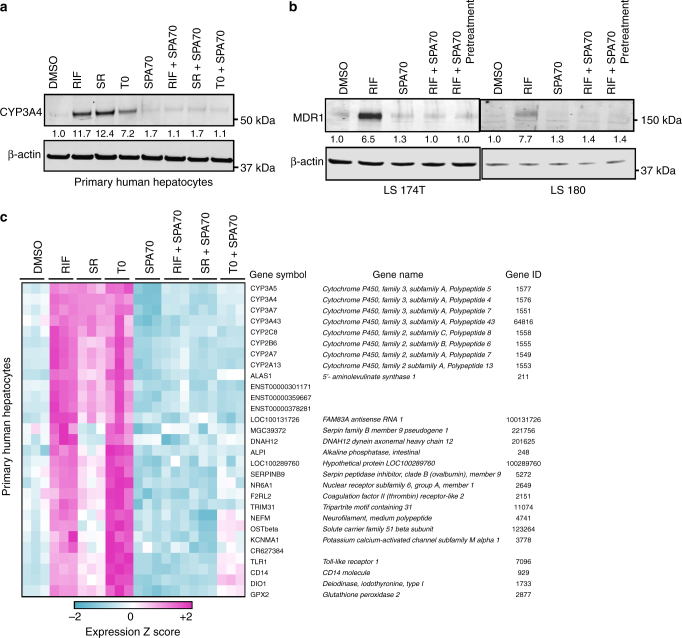
SPA70 antagonizes hPXR target genes induced by various hPXR agonists. **a** CYP3A4 protein levels in primary human hepatocytes treated with vehicle control (0.1% DMSO), 1 μM rifampicin (RIF), 0.1 μM SR12813 (SR) or 0.03 μM T090137 (T0) in the presence or absence of 2.5 μM SPA70 for 48 h. Primary human hepatocytes from at least three donors were used; representative data from donor #HH1811-1 are shown. **b** MDR1 protein levels in LS180 and LS174T cells treated for 72 h with 1.6 μM RIF and 10 μM SPA70 alone or in combination (RIF + SPA70: simultaneous co-treatment; RIF + SPA70 pretreatment: cells treated with SPA70 24 h before RIF treatment). Final DMSO concentration was 0.2% in both **a**, **b**. The levels of β-actin were used as loading controls. The numbers between the blots indicate the relative intensity of the protein bands (CYP3A4 or MDR1) in the respective lanes, with the DMSO-treated sample being set as 1.0. **c** DNA microarray analysis (with representative results displayed as a heatmap), showing the expression of hPXR transcriptional target genes in human hepatocytes in response to hPXR agonists (RIF, SR or T0) with or without SPA70 co-treatment

To confirm the effect of SPA70 on the transcriptional activity of endogenous hPXR, we used primary human hepatocytes. As shown in Fig. 2a, the induction of CYP3A4 in primary human hepatocytes by rifampicin was substantially blocked by SPA70. Interestingly and importantly, SPA70 was able to block the CYP3A4-inducing effect of two other structurally different hPXR agonists, SR12813 and T090137, thereby demonstrating the potential of SPA70 to antagonize a broad range of structurally diverse hPXR agonists (Fig. 2a). We further confirmed the activity of SPA70 in blocking the agonist-induced transcriptional activity of hPXR, and we demonstrated that SPA70 was able to block rifampicin induction of *MDR1*, another hPXR transcriptional target, in the human colorectal adenocarcinoma cell lines LS180 and LS174T (Fig. 2b and Supplementary Fig. 2).

To determine whether SPA70 blocked the expression of all genes induced by hPXR agonists, we used human primary hepatocytes in DNA microarray analysis. As shown in Fig. 2c, a broad range of hPXR transcriptional target genes were induced by the structurally different hPXR agonists rifampicin, SR12813 and T090137, but SPA70 blocked their induction. Taken together, these data indicate that SPA70 is a low toxic, specific antagonist of hPXR that blocks the induction of expression of a broad range of hPXR transcriptional target genes by several hPXR agonists.

### SPA70 blocks hPXR-mediated drug metabolism in vivo

**Fig. 3:**
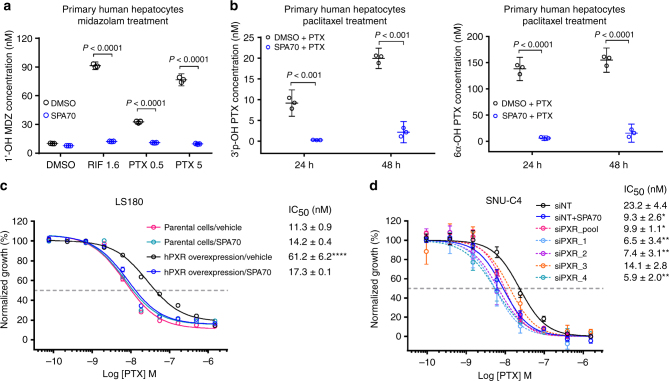
SPA70 reduces the metabolism and enhances the efficacy of chemotherapeutic agents. **a**, **b** SPA70 reduces the metabolism of paclitaxel (PTX) in primary human hepatocytes (PHHs). In **a**, PHHs were pretreated for 72 h with 0.1% DMSO, 1.6 µM RIF or 0.5 µM or 5 µM PTX in the presence or absence of 10 µM SPA70 before receiving 3.3 µM midazolam (MDZ) and being incubated for a further 4 h. The concentrations of 1′-OH MDZ (an MDZ metabolite) in the medium for each treatment were determined using LC/MS/MS. In **b**, PHHs were treated with 1 µM PTX in the presence or absence of 10 µM SPA70. The concentrations of PTX metabolites, 3-p-OH PTX (generated by CYP3A4) and 6α-OH PTX (generated by CYP2C8), were determined using LC/MS/MS. Each data point represents the compound concentration measured after 24 or 48 h of treatment. Data are expressed as the mean and 95% confidence interval (CI). **c**, **d** hPXR levels inversely correlate with the sensitivity of cancer cells to PTX. In **c**, LS180 cells with (hPXR overexpression) or without (parental cells) stable exogenous expression of hPXR were treated with PTX in the presence or absence of 10 µM SPA70 for 96 h before a CellTiter-Glo assay was performed. The vehicle was DMSO. In **d**, SNU-C4 cells treated with pooled siRNAs (siPXR_pool) or individual siRNAs (siPXR_1, 2, 3 and 4) targeting hPXR, or with nontargeting controls (siNT) with or without SPA70, were treated with PTX for 96 h before a CellTiter-Glo assay was performed. The ‘% normalized growth’ was determined by normalizing the luminescent signal from compound-treated cells to that from DMSO-treated cells. The drug concentration is expressed using a log scale. Final DMSO concentration was 0.2% in **a**–**d**. The half-maximal inhibitory concentration (IC_50_) is indicated. Data are presented as the means ± S.E.M. (*n* = 4 in **c** and *n* = 3 in **d**). *P < 0.05, **P < 0.01 or ****P < 0.0001 for each group relative to the parental cells/vehicle in **c** or siNT-treated cells in **d**. Statistical significance was calculated by one-way analysis of variance with Tukey’s multiple-comparison test

To determine the blocking effect of SPA70 on hPXR-mediated drug metabolism, we first examined the effect of SPA70 on hPXR agonist-induced metabolism of midazolam, a CYP3A4 substrate^28, 29^, in primary human hepatocytes. As shown in Fig. 3a, the hPXR agonists rifampicin and paclitaxel both robustly induced the metabolism of midazolam to 1′-OH-midazolam. Both rifampicin- and paclitaxel-induced metabolism of midazolam were substantially blocked by SPA70 (Fig. 3a). Paclitaxel itself is a substrate of CYP3A4^30^ and, as expected, the metabolism of paclitaxel to 3′-p-hydroxypaclitaxel or 6α-hydroxypaclitaxel was substantially blocked by SPA70 (Fig. 3b).

It was previously reported that knocking down hPXR in cancer cells enhanced the efficacy of chemotherapeutic agents that are substrates of CYP3A4^19^. The blocking effect of SPA70 on the metabolism of paclitaxel led us to investigate whether SPA70 enhanced the efficacy of chemotherapeutic agents that were also CYP3A4 substrates. As shown in Fig. 3c, the human colorectal adenocarcinoma cell line LS180 is sensitive to paclitaxel (IC_50_ = 11.3 nM). Exogenously expressed hPXR decreased this sensitivity (IC_50_ = 61.2 nM), which is consistent with the known role of hPXR in regulating drug sensitivity. As expected, SPA70 alone was marginally toxic (the IC_50_ value of paclitaxel is 11.3 nM in the absence of SPA70 and 14.2 nM in its presence); however, it abolished the effect of exogenous hPXR and re-sensitized the cells to paclitaxel, further confirming its hPXR antagonistic effect. The cytotoxic effect of vincristine and vinblastine on LS180 cells exogenously expressing hPXR was also enhanced by SPA70; SPA70 decreased the IC_50_ value of vinblastine from 16.9 nM to 5.0 nM (*n* = 4, *P* < 0.01), and decreased the IC_50_ value of vincristine from 52.2 to 11.8 nM (*n* = 4, *P* < 0.0001) (Supplementary Fig. 3a). Analysis using the Cancer Cell Line Encyclopedia database identified the human colorectal adenocarcinoma cell line SNU-C4 and the human colorectal carcinoma cell line LS1034 as expressing high levels of hPXR^31^. Knocking down hPXR in SNU-C4 cells by using siRNA sensitized the cells to paclitaxel and vincristine, and as expected, SPA70 mimicked the genetic downregulation of hPXR in sensitizing the cells to paclitaxel and vincristine (Fig. 3d and Supplementary Fig. 3b). Similarly, SPA70 sensitized LS1034 cells to both paclitaxel and vincristine (Supplementary Fig. 3c). Supplementary Fig. 4 shows the hPXR levels in the cell models used, with and without genetic modulation.

**Fig. 4:**
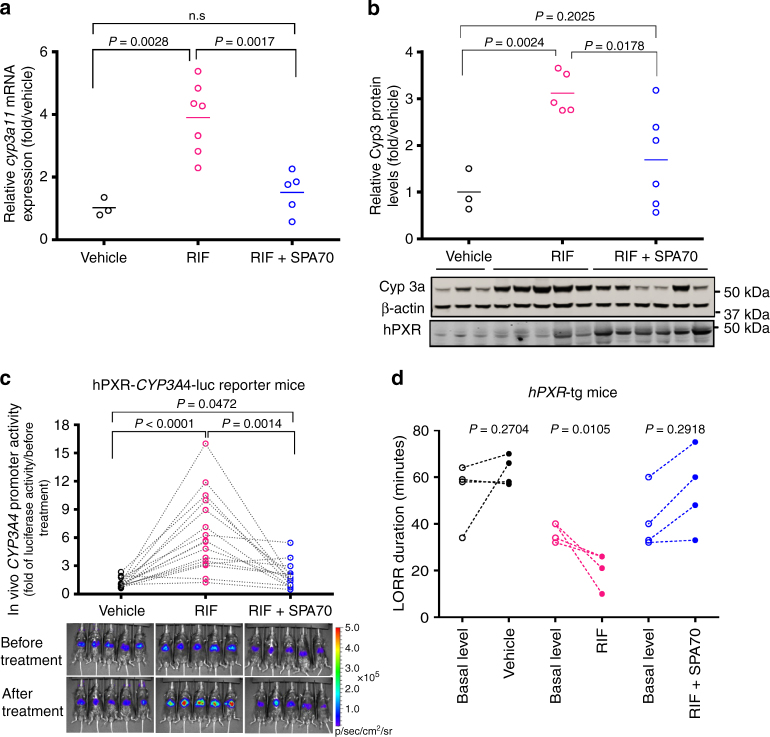
SPA70 antagonizes hPXR-mediated drug metabolism in humanized PXR mouse models. **a** Liver *Cyp3a11* mRNA was analyzed by real-time PCR in hPXR transgenic (hPXR-tg) mice treated for 72 h with vehicle control (Vehicle), rifampicin (RIF) (10 mg kg^−1^), or RIF (10 mg kg^−1^) plus SPA70 (200 mg kg^−1^). **b** Liver CYP3A protein levels in the hPXR-tg mice described in **a** were determined by western blotting. Each data point represents the level of *Cyp3a11* mRNA **a** or protein **b** from an individual mouse; the *short horizontal lines* indicate the mean value for three to seven mice from the experimental group. Representative western blots from each group are shown in the *lower* panel of **b**. **c** The *hPXR-CYP3A4-luc* reporter mice (*n* = 15) were generated by hydrodynamic injection of the pGL3-*CYP3A4-luc* reporter and pcDNA3-*hPXR*. Each mouse received three rounds of sequential treatment with vehicle control (Vehicle), RIF (10 mg kg^−1^) or RIF (10 mg kg^−1^) plus SPA70 (150 mg kg^−1^) every 24 h for 2 days, with a washout period of 72 h between each treatment round. The luciferase activity in these mice was recorded by bioluminescence imaging 10 h after the last treatment in each round, and the induction of *CYP3A4-luc* activity was calculated as described in Methods. Each data point represents the *CYP3A4* promoter reporter activity in an individual mouse, and each *dotted line* indicates the change in luciferase activity between rounds of treatment for the same mouse. The bioluminescence images of luciferase activity from five representative *hPXR-CYP3A4-luc* reporter mice after each treatment are shown. The rainbow scale represents relative light units (photon flux per second per square centimeter), positively reflecting the luciferase activity. **d** Duration of the loss of righting reflex (LORR), recorded as described in Methods to measure the metabolism of the anesthetic (2,2,2-tribromoethanolamine) in hPXR-tg mice before (basal level) and after treatment with vehicle, RIF, or RIF plus SPA70. Each data point represents the LORR duration in an individual mouse; the *lines* indicate the LORR duration change in individual mice before and after treatment. *P-*values were obtained for the two indicated treatment groups by using a paired *t*-test

The blocking effect of SPA70 on hPXR-mediated effects in cancer cell lines and primary hepatocytes prompted us to examine the hPXR antagonistic effect of SPA70 in vivo by using mouse models. We first determined the maximum tolerated dose of SPA70. No detrimental health effects were observed at 500 mg kg^−1^, which was the highest dose tested (Supplementary Fig. 5). Oral administration of SPA70 at 500 mg kg^−1^ led to an increase in mouse body weight at days 13 and 15 (*P* < 0.05). At a dose of 200 mg kg^−1^, SPA70 effectively blocked the rifampicin-induced expression of Cyp3a11, a mouse homolog of human CYP3A4, at both the mRNA (Fig. 4a) and protein (Fig. 4b and Supplementary Fig. 2) levels in an hPXR humanized transgenic mouse model^14^. We noticed that hPXR protein levels elevated in mice treated with both rifampicin and SPA70 (Fig. 4b). SPA70 is a high-affinity ligand for hPXR, and stabilization of receptor induced by high-affinity ligands has been observed in other nuclear receptors such as VDR^32^ and PPARδ^33^, possibly by blocking ubiquitination. Similarly, in a transient transgenic mouse model created by hydrodynamic injection^34^, 150 mg kg^−1^ of SPA70 effectively blocked the rifampicin-induced activation of the *CYP3A4* promoter (Fig. 4c). The loss of righting reflex (LORR) assay has been used to monitor the in vivo metabolism of 2,2,2-tribromoethanol by Cyp3a11^35^. In the hPXR humanized mouse model, the rifampicin-induced metabolism of 2,2,2-tribromoethanol by Cyp3a11 was effectively blocked by SPA70 (Fig. 4d). Together, these data demonstrate that SPA70 antagonizes hPXR in primary human hepatocytes, cancer cell lines, and animal models.

### Unexpected agonistic activity of close analogs of SPA70

Sulforaphane and ketoconazole are the most comprehensively studied of the reported hPXR antagonists, but they are not effective in vivo^24–26^. The blocking activity of SPA70 on hPXR-mediated drug metabolism in vivo led us to further investigate its SAR by designing and evaluating analogs of SPA70 (Supplementary Table 1). Surprisingly, subtle structural modifications dramatically affected the activity of the analogs. Whereas some SPA70 analogs (such as SJC2) maintained the hPXR antagonistic activity, others (such as SJA1) exhibited decreased hPXR antagonistic activity (Supplementary Table 1). Unexpectedly, we found that some SPA70 analogs acted as hPXR agonists (Supplementary Table 1). For example, although SJB7 (also known as LC-24) and SPA70 have comparable activities in a cell-free competitive hPXR binding assay (with IC_50_ values of 750 nM and 540 nM, respectively), SJB7 is an agonist of hPXR in cell-based assays (EC_50_ = 880 nM). SPA70 differs structurally from SJB7 only by lacking the para-methoxy group and by having a meta-methoxy group instead of a meta-methyl group (Supplementary Table 1). Although hPXR was previously known for its ligand promiscuity, resulting in its SAR being considered difficult to study, our observation that SPA70 and its close analogs display differing hPXR antagonistic and even agonistic activities demonstrates the feasibility of studying the SARs of hPXR-ligand interactions.

### SPA70 and SJB7 affect cofactor association with hPXR

The transcriptional activity of hPXR is enhanced by co-activators such as SRC-1 and TIF2, but it is inhibited by co-repressors such as NCoR and SMRT. Therefore, we examined the effect of SPA70 on the association of hPXR with NCoR and SMRT, along with the effect of SJB7 on the association of hPXR with SRC-1 and TIF2. As shown in Supplementary Fig. 6a, in a mammalian two-hybrid assay, SPA70 increased the association of hPXR with SMRT 1.8-fold (*P* = 0.0003) and the association of hPXR with mNCoR 2.1-fold (*P* = 0.0002), whereas SJB7 increased the association of hPXR with SRC-1 1.8-fold (*P* = 0.0001) and increased the association with TIF2 1.6-fold (*P* = 0.0001). SJB7 did not affect the association of SMRT or mNCoR with hPXR, and SPA70 did not affect the association of SRC-1 or TIF2 with hPXR. Similarly, in a TR-FRET biochemical cofactor recruitment assay, SPA70 enhanced the hPXR–NCoR interactions (NCoR interaction domain 2 [NCoRID2]: DPASNLGLEDIIRKALMGSFDDK), whereas SJB7 enhanced the hPXR–SRC-1 interactions (SRC-1: CPSSHSSLTERHKILHRLLQEGSPS) (Supplementary Fig. 6b). Consistent with the agonistic activity of SJB7 and the antagonistic activity of SPA70, Supplementary Fig. 6c shows that SPA70 could effectively block the activation of hPXR by its agonistic analog SJB7. Taken together, these data indicate that SPA70 enhances the association of hPXR with co-repressors, thereby contributing to its antagonistic effect, whereas SJB7 enhances the association of hPXR with co-activators, thereby contributing to its agonistic effect.

### SJB7 co-crystalizes with hPXR

**Fig. 5:**
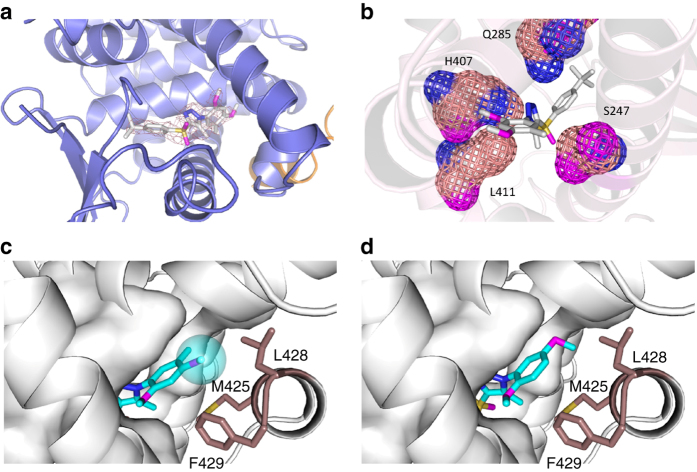
Crystal structure of the hPXR LBD in complex with SJB7 and the SRC-1 coactivator peptide. **a** 2Fo-Fc map (1.0 sigma) around SJB7. The hPXR LBD is depicted in *blue* and SRC-1 in *orange*. **b** SJB7 is fixed in a tunnel created by S247, Q285, H407 and L411 (shown as *meshes*). **c** SJB7 interacting with the AF-2 helix residues M425, L428 and F429. The *sphere* indicates the methoxy group that contacts the L428 and F429 pair. **d** The antagonist SPA70 docked in the ligand-binding site, showing the missing interactions with L428 and F429. Color code for the stick representations: *blue*, nitrogen; *magenta*, oxygen; *yellow*, sulfur

Multiple co-crystal structures of the hPXR ligand-binding domain (LBD) in complex with its agonist have been reported, with an SRC-1 peptide typically being used to increase the stability and solubility of hPXR^36, 37^. Similarly, we determined the crystal structure of the hPXR LBD in complex with the agonist SJB7 and the co-activator peptide SRC-1 (CPSSHSSLTERHKILHRLLQEGSPS) at a resolution of 2.66 Å (Fig. 5 and Table 1). The hPXR LBD displays the canonical α-helical sandwich common to nuclear receptors, along with the characteristic five-stranded antiparallel β-sheet unique to hPXR. The complex exhibits a homodimer in the asymmetric unit, as observed in a number of previously reported hPXR LBD structures^38^, with the dimer interface being formed by the β1′-strands from each monomer. Protein chains A and B are similar in structure, sharing a root mean square deviation of 0.727 Å over all atoms. The hPXR LBD adopts an active conformation with the AF-2 helix (helix 12) positioned to favor co-activator binding, in which the SRC-1 peptide is seen packed against the AF-2 groove of each LBD.

**Table 1:** Data collection and refinement statistics

	hPXR LBD-SRC1-SJB7
*Data collection*	
Space group	P2_1_2_1_2_1_
Cell dimensions	
*a*, *b*, *c* (Å)	84.34, 89.30, 106.76
α, β, γ (°)	90.00, 90.00, 90.00
Resolution (Å)	2.66 (2.76–2.66)^a^
*R*_merge_ (%)	10.8 (31.3)
*I*/σ*I*	9.2 (6.0)
Completeness (%)	97.3 (91.3)
Redundancy	5.4 (5.4)
*Refinement*	
Resolution (Å)	45.15–2.66
No. reflections	19,141
*R*_work_/*R*_free_	0.20/0.25
No. atoms	4199
Protein	4119
Ligand	60
Water	20
*B*-factors	
Protein	54.03
Ligand	85.01
Water	49.96
R.m.s. deviations	
Bond lengths (Å)	0.013
Bond angles (°)	1.538

^a^Values in parentheses are for the highest-resolution shell

SJB7 resides in the highly hydrophobic ligand-binding site, with no hydrogen bond having been clearly observed (Fig. 5a). The *t*-butyl group makes hydrophobic contacts with W299, which interacts with most, if not all, of the ligands co-crystallized to date (Supplementary Fig. 7)^39, 40^. The ring of the *t*-butylphenyl group interacts with M243 and M323, with L209, V211, Y306 and L308 providing additional contacts to enclose the *t*-butylphenyl moiety. The triazole group is fastened in place by a narrow channel that is ~8–9 Å wide and is formed by S247, Q285, H407 and L411 (Fig. 5b), which reflects the plasticity of the hPXR LBD in molding to the shape of the ligand and may contribute to the favorable positioning of key protein–ligand interactions. Even though the residue H407 has been previously reported to participate in hydrogen bonding with several ligands, it resides at some distance (3.7 Å) from the sulfonyl group of SJB7. However, we cannot discount the possibility that interactions between the sulfonyl moiety and H407 are dependent on the histidine protonation states and the potential rotation of the residue to shorten the intervening distance (the H407 residues in the two chains are seen in different rotations).

It is significant that SJB7 appears to interact with the residues M425, L428 and F429 from the AF-2 helix (Fig. 5c), thereby stabilizing the LBD in the active conformation for coactivator binding. The dimethoxy-methylphenyl moiety forms π-π interactions with F429, in addition to F281, whereas L428 and F429 form contacts with the para-methoxy group, which is also engaged with A280. In addition, the residue M425 appears to interact with the methyl group in the triazole moiety. The 5-methyl group in the phenyl ring appears to reside loosely in a small cavity whose mouth is formed mainly by residues S247, F251, M425 and L428. The co-crystal structure of SJB7-hPXR presented here, combined with data from hydrogen/deuterium exchange (HDX) coupled with mass spectrometry (MS), as discussed below, provides insights into the molecular basis of antagonism by SPA70 (Fig. 5d).

### A model for SJB7 agonism and SPA70 antagonism

**Fig. 6:**
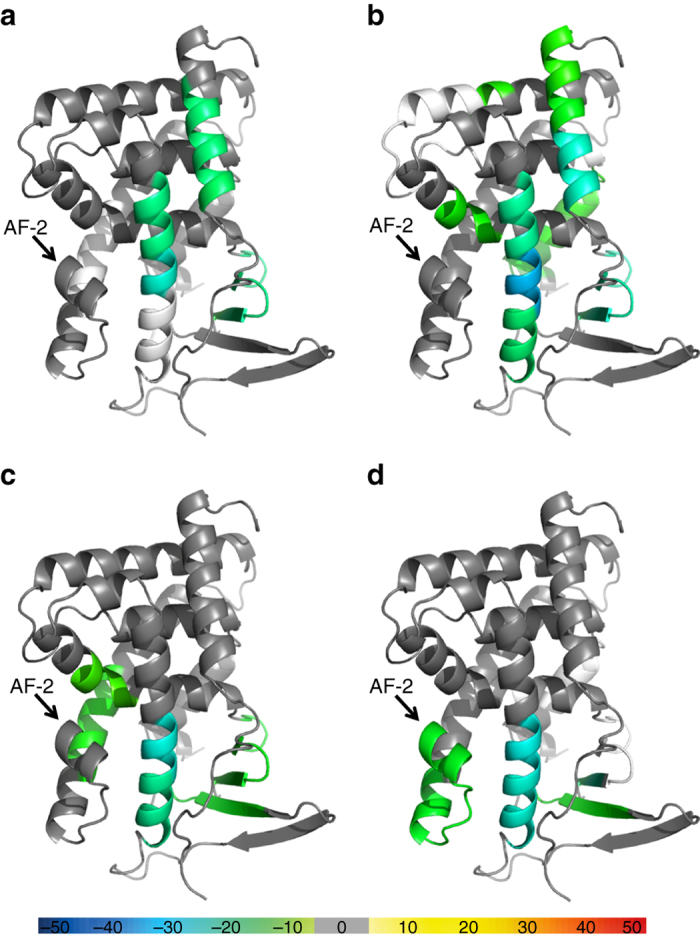
HDX studies indicating structural rigidity changes in the hPXR LBD due to the presence of SJB7 and SPA70. Overlays of the differential HDX data onto the crystal structure of the hPXR LBD (PDB code 5X0R) for **a** SJB7, **b** SPA70 **c** SJB7 with the subsequent addition of SRC-1, and **d** SPA70 with the subsequent addition of NCoR. The structures are color-coded according to the color bar at the bottom of the figure. Regions that were not covered are represented in *white*. See Supplementary Fig. 8 for additional information on the color coding and score used

No co-crystal structure of hPXR with an hPXR antagonist has been reported. Because we were unable to obtain crystals of the hPXR LBD in complex with SPA70 by using the conditions for co-crystallization with an agonist (e.g., by using SRC-1 peptide to improve protein solubility), we employed HDX coupled with MS, a technique successfully used to analyze protein–ligand interactions^41^. Importantly, HDX can probe the effect of protein structural dynamics and stability upon ligand binding to the hPXR LBD, providing information that is complementary to crystallographic data regarding protein–ligand interactions. The HDX experiments were conducted in the presence or absence of ligand and with or without the subsequent addition of SRC-1 peptide (CPSSHSSLTERHKILHRLLQEGSPS) for SJB7 or NCoR1-3 peptide (ASNLGLEDIIRKALMGSFD) for SPA70. HDX analysis indicates that adding SJB7 or SPA70 leads to an increase in protein rigidity and the loss of structural flexibility (Fig. 6a, b and Supplementary Fig. 8). In the presence of SJB7, protection from solvent exchange has been observed in three regions in the hPXR LBD, encompassing residues 150–161, 238–257 and 293–307, which suggests that SJB7 binds directly to the hPXR LBD (Fig. 6a). The reduced HDX kinetics are consistent with the crystal structure, in which W299, M243, S247 and Y306 derived from the ligand-binding pocket form extensive hydrophobic interactions with SJB7.

Differential HDX profiles in the presence of SPA70 (Fig. 6b) reveal increased rigidity in areas (residues 144–161, 238–257 and 293–307) similar to those affected by SJB7, with additional protected sections being observed in the peptides corresponding to residues 276–281, 319–335 and 349–368. The increase in structural compactness due to the presence of SPA70 indicates that direct binding of SPA70 occurs in a region in the ligand-binding pocket similar to that where SJB7 resides. The subsequent addition of SRC-1 to the hPXR LBD–SJB7 mixture resulted in a few more areas of protection from solvent exchange, including residues 210–215, 276–281 and 401–411 (Fig. 6c); however, no further protection was observed in the vicinity of the AF-2 groove. In contrast, adding NCoR to the hPXR LBD–SPA70 complex (Fig. 6d) decreased the fluidity of the AF-2 helix (residues 412–428). On the basis of our biochemical and cellular data, the binding of SRC-1 or NCoR to the hPXR LBD was enhanced by the presence of an agonist or antagonist, respectively, and the perturbations observed in the HDX experiments due to SRC-1 and NCoR further confirm that SPA70 interacts directly with the hPXR LBD.

The analogs SJB7 and SPA70 are structurally very similar, yet SJB7 is an agonist and SPA70 an antagonist of hPXR. Whereas SJB7 contains a 2,4-dimethoxy-5-methylphenyl component, SPA70 contains a 2,5-dimethoxy phenyl component with the 5-methoxy (meta-methoxy) group replacing the 5-methyl group in SJB7. With these few exceptions, the rest of the chemical scaffold of both molecules remains structurally the same. We hypothesize that because SPA70 lacks the 4-methoxy (para-methoxy) group that is directed towards the AF-2 helix, SPA70 fails to stabilize the AF-2 helix for co-activator binding because there are no interactions with the L428 and F429 pair that forms a hydrophobic ‘spot’. Without these interactions, the AF-2 helix can move freely, leading to disorder in the region allocated for co-activator binding. Figure 5d illustrates SPA70 docked in the hPXR LBD, lacking the concerted interactions with the ‘spot’ formed by L428 and F429. The residue M425 is part of the AF-2 helix and also forms a hydrophobic contact with SPA70 through the methyl group on the triazole moiety, as with SJB7. However, unlike L428 and F429, M425 resides at the beginning of the AF-2 helix and may not be sufficient to stabilize the helix in its active form. The small cavity into which the 5-methyl group in the phenyl ring of SJB7 projects is spacious enough to accommodate larger groups such as the 5-methoxy group of SPA70. Although the accommodation of such larger groups in the small cavity might not contribute significantly to stabilizing AF-2 for co-activator binding, it may provide opportunities to improve the compound binding affinity. This concept of converting an agonist into an antagonist by weakening or abolishing interactions between the ligand and the terminal helix 12 (AF-2) is reminiscent of VDR antagonists, which unlike their agonist counterparts, have insufficient interactions with residues in the AF-2 helix^42, 43^.

## Discussion

Our biochemical, cellular, and in vivo characterization identified SPA70 as the most potent and selective hPXR antagonist and one of the least toxic characterized to date. Supplementary Table 2 summarizes the reported hPXR antagonists. SPA70 is a submicromolar antagonist of hPXR in both biochemical and cell-based assays, exhibiting no detectable agonistic or antagonistic activity against a panel of seven other human nuclear receptors and having only marginal cytotoxicity in the cell models used. SPA70 is a very weak CAR inverse agonist (Fig. 1b) and a very weak mPXR agonist only at concentrations higher than 5 µM (Fig. 1c). Interestingly, SPA70 weakly promoted the agonistic effect of T0901317 on LXRβ (Fig. 1c). Our TR-FRET competitive binding data (Fig. 1b and Supplementary Table 1) suggest that SPA70 is a competitive antagonist. The decline in the basal activity of hPXR in the cell-based assay with SPA70 (Fig. 1b) might indicate that SPA70 has an antagonistic effect on the basal agonistic activity in that assay system. It is also possible that SPA70 enhances the recruitment of co-repressors to reduce the basal hPXR activity, and thus function as an inverse agonist. Most importantly, the hPXR antagonistic activity and low cellular toxicity were reproducible in a humanized hPXR mouse model: when we tested SPA70 at a dose of 500 mg kg^−1^, which was the highest dose tested, we observed no detrimental health effects in mice, and SPA70 effectively blocked the rifampicin-induced activation of hPXR in hPXR transgenic mouse models at doses of 150 and 200 mg kg^−1^. Furthermore, our design and characterization of analogs of SPA70 led us to SJB7, a close analog of SPA70 that is an agonist of hPXR. By crystal structural studies, we demonstrated that SJB7 resides in the ligand-binding site. Among other interactions revealed by the co-crystalized structure, SJB7 interacts with residues M425 (via the methyl group on the triazole moiety) and L428 and F429 (via the para-methoxy group on the dimethoxy-methylphenyl moiety) of the AF-2 helix to stabilize the LBD in the active conformation for co-activator interaction. Analogs with a relatively bulky hydrophobic substituent in place of the hydrogen at position R1 in the phenyl group of SPA70 (Supplementary Table 1) may display agonistic behavior by forming additional interactions with residues such as L428 and F429 to stabilize the hPXR AF-2 helix, whereas analogs with an antagonistic character possess a substituent, such as fluorine (F), chlorine (Cl) or a methyl group (Me), that is too small to increase any significant stabilization of the hPXR AF-2 helix. We used HDX-MS to obtain complementary information on SJB7-hPXR interaction, which was consistent with information derived from the crystal structure. HDX analysis revealed regions of hPXR that are similarly protected by SPA70 and SJB7, indicating that SPA70 and SJB7 bind to common regions of the hPXR ligand-binding pocket.

On the basis of the subtle differences in the chemical structures of the agonist and antagonist, on the results of hPXR LBD crystallography, and on HDX analysis, we have proposed a model to explain the antagonistic activity of SPA70 (Fig. 5d). Briefly, both SJB7 and SPA70 interact with the M425 residue through the methyl group on the triazole moiety, but such interaction is insufficient to stabilize the AF-2 helix for coactivator recruitment and render agonistic activity. Whereas SJB7 further interacts with a hydrophobic ‘spot’ comprised of residues L428 and F429 to stabilize the AF-2 helix for co-activator recruitment through the para-methoxy group, SPA70 fails to do so because it lacks the para-methoxy group. Our data also show that SPA70 facilitates the recruitment of co-repressor to suppress hPXR activity. Supported to a large extent by our HDX results, we conclude that the hPXR LBD is structurally more fluid and less rigid in the absence of any ligand, which would limit the binding of co-regulatory peptides. It is, therefore, conceivable that SPA70 stabilizes the hPXR LBD, to the extent that NCoR binding is enhanced, by establishing an extensive network of internal interactions that are bridged by the ligand and acting as a ‘glue’ for added structural stability. The argument that interaction with M425 is insufficient for SPA70 to stabilize the AF-2 helix for co-activator interaction but enables SPA70 to competitively prevent agonist binding and thereby carry out its antagonistic function is supported by data presented in Supplementary Fig. 6c, which shows that SPA70 could effectively block hPXR activation by its agonistic analog SJB7. Because of the loss of function when M425 or F429 was mutated (as revealed by the activity of hPXR M425A or hPXR F429A in Supplementary Fig. 6a, c), we were unable to functionally investigate the effect of mutating M425 or F429 on the interaction of hPXR with SPA70 or SJB7.

Taken together, our findings have characterized a potent, specific hPXR antagonist that is active in cell and animal models. SAR analysis suggests that subtle structural modification of SPA70 (to become SJB7) can dramatically change the cellular activity of the analogs. Therefore, the cellular outcome of ligand binding is probably determined by the conformational change induced by the ligand. Structural analysis provides evidence that SJB7 interacts directly with key residues of the hPXR ligand-binding pocket and predicts that SPA70 interacts directly with hPXR, but the effect of SPA70 on AF-2 and cellular activity differs from that of SJB7. Our discovery of structurally similar analogs that modulate hPXR in opposing ways as a result of subtle chemical structure modifications represents a considerable advance in the development of chemical probes for hPXR and reveals the possibility of studying the SARs of the promiscuous PXR. Biological data in conjunction with protein–ligand structural information can provide avenues to increase the potency of hPXR antagonists. It is anticipated that exploring further modifications will result in compounds that demonstrate increased potency, particularly through enhancing the formation of favorable hydrogen-bond interactions with residues such as H407. Encouraged by the activities of the SPA70 analogs that we have developed so far, we are designing more analogs, with a view to further increasing their efficiency/efficacy in antagonizing hPXR. However, in addition to efficiency/efficacy, we will continue to emphasize selectivity and low toxicity. The ability of SPA70 to sensitize drug-resistant cancer cells confirms the role of hPXR in regulating the drug sensitivity of cancer cells and further validates the concept of using hPXR antagonists as co-drugs to improve therapeutic efficacy. The structural and functional regulation of hPXR warrant further investigation, and SPA70 and its analogs will facilitate these efforts.

## Methods

### Representative chemical synthesis

Details on the general chemical synthesis procedures employed^44^ can be found in the Supplementary Information. The chemical SPA70 was prepared by following a published protocol^45^ or purchased from ChemDiv (San Diego, CA). The chemical SJB7 was prepared by following a similar protocol to that used to prepare SPA70 (Supplementary Fig. 9) but with modifications. Briefly, the compound 4-bromo-2-methoxy-5-methylaniline (**89d**) was first converted to 1-azido-4-bromo-2-methoxy-5-methylbenzene (**90d**) via diazonium salts. The resulting bromo-azide compound **90d** was then converted to the bromo-triazole compound **SJB3** by coupling with 1-((4-(tert-butyl)phenyl)sulfonyl)propan-2-one. The resulting bromo-triazole compound **SJB3** was then further converted to the product **SJB7** via a copper-catalyzed reaction^46^.

1-(4-bromo-2-methoxy-5-methylphenyl)-4-((4-(tert-butyl)phenyl)sulfonyl)-5-methyl-1H-1,2,3-triazole (SJB3): NaNO_2_ (20 g, 0.29 mol) in water (100 ml) was added to a solution of **89d** (4-bromo-2-methoxy-5-methyl-aniline, 48 g, 0.22 mol) in concentrated HCl (200 ml) at 0 °C, and the reaction mixture was then stirred for 15 min at 0 °C. A solution of NaN_3_ (62 g, 0.95 mol) in water (150 ml) was added dropwise. The solution was then stirred for 2 h at ambient temperature. The resulting precipitate was collected by filtration and washed with ice water. The solid was dried to give product **90d** (38 g, 71% yield). ^1^H NMR (400 MHz, CDCl_3_) *δ* (ppm) 7.01 (s, 1H), 6.83 (s, 1H), 3.82 (s, 3H), 2.28 (s, 3H).

MeONa (15 g, 0.28 mol) and 1-((4-(tert-butyl)phenyl)sulfonyl)propan-2-one (20 g, 79 mmol) were added to a solution of compound **90d** (16.5 g, 68 mmol) in MeOH. The mixture was stirred at 60 °C overnight and then poured into water. The precipitate was collected by filtration. The crude product was washed with water then with MeOH to give product **SJB3** (14.6 g, 45% yield, 96.0% purity). ^1^H NMR (CDCl_3_, 400 MHz): *δ* (p.p.m.) 8.02 (dd, 2H, *J = *8.8 Hz, 2 Hz), 8.55 (dd, 2H, *J = *8.8 Hz, 2 Hz), 7.24 (s, 1H), 7.17 (s, 1H), 3.76 (s, 3H), 2.42 (s, 3H), 2.34 (s, 3H), 1.32 (s, 9H). ^13^C NMR (126 MHz, DMSO-*d*_*6*_) *δ* (ppm) 157.86, 152.50, 143.59, 139.25, 138.23, 130.60, 130.52, 127.95, 127.64, 127.13, 122.38, 117.24, 57.16, 57.15, 35.50, 31.12, 21.66, 8.97. ESI-TOF HRMS: *m*/*z* 478.0790 (C_21_H_24_BrN_3_O_3_S + H^+^ requires 478.0800).

4-((4-(tert-butyl)phenyl)sulfonyl)-1-(2,4-dimethoxy-5-methylphenyl)-5-methyl-1H-1,2,3-triazole (SJB7): MeONa (130 mg, 2.4 mmol) and CuCl_2_ (119 mg, 1.2 mmol) were added to a solution of compound **SJB3** (300 mg, 0.6 mmol) in DMF (10 ml). The reaction mixture was stirred overnight at 130 °C. It was then poured into water and extracted with EtOAc. The EtOAc layer was washed with water, dried with anhydrous Na_2_SO_4_, and concentrated with a rotary evaporator. The residue was purified by preparative HPLC to give product **SJB7** (30 mg, 11.7% yield, 97.5% purity). ^1^H NMR (CDCl_3_, 400 MHz): *δ* (p.p.m.) 7.97 (d, 2H, *J = *8.4 Hz), 7.49 (d, 2H, *J = *8.4 Hz), 6.97 (s, 1H), 6.44 (s, 1H), 3.84 (s, 3H), 3.71 (s, 3H), 2.36 (s, 3H), 2.08 (s, 3H), 1.27 (s, 9H). ^13^C NMR (126 MHz, CDCl_3_) *δ* (ppm) 159.386, 156.463, 152.089, 142.885, 137.533, 137.198, 128.544, 126.686, 125.241, 118.416, 114.416, 94.310, 55.028, 54.753, 34.241, 30.048, 14.140, 7.942. ESI-TOF HRMS: *m*/*z* 430.1790 (C_22_H_27_N_3_O_4_S + H^+^ requires 430.1800). See Supplementary Methods for the synthesis of other compounds. ^1^H and ^13^C NMR spectra are shown in Supplementary Fig. 10.

### Materials and statistics

GST-PXR-LBD, Tb-anti-GST, Tris-HCl, pH 7.5 (1 M), DTT (1 M), bovine serum albumin (BSA), fluorescein–NCoR ID2 corepressor peptide (F-NCoRID2), phenol red-free DMEM (Dulbecco's modified eagle medium), Lipofectamine 3000 and charcoal/dextran-treated fetal bovine serum (FBS) were purchased from Thermo Fisher Scientific (Waltham, MA). The 384-well low-volume solid black plates, tissue culture-treated 384-well black clear-bottom plates, and tissue culture-treated poly-D-lysine-coated 384-well black clear-bottom plates were purchased from Corning Life Sciences (Tewksbury MA). DMSO, MgCl_2_, rifampicin, 1α,25-dihydroxyvitamin D3, 9-*cis*-retinoic acid, GW4604, dexamethasone, T0901317, rosiglitazone, vincristine, vinblastine, paclitaxel, 3′-p-hydroxypaclitaxel, pregnenolone-16α-carbonitrile (PCN) and SR12813 were purchased from Sigma (St. Louis, MO). Tissue culture-treated 384-well white solid-bottom plates and steadylite HTS reagent were purchased from PerkinElmer Life Sciences (Hopkinton, MA). FuGENE 6 was purchased from Roche (Indianapolis, IN). Dual-Glo luciferase assay reagent and CellTiter-Glo luminescent cell viability assay reagent were purchased from Promega (Madison, WI). Staurosporine was purchased from LC Laboratories (Woburn, MA). 6α-hydroxypaclitaxel was purchased from Cayman Chemical (Ann Arbor, MI). Midazolam and 1-OH midazolam were obtained from BD Gentest (Woburn, MA). Midazolam-D4 maleate was obtained from Cerilliant (Round Rock, TX).

HepG2 (ATCC CRL-10741), HEK293 (ATCC CRL-1573), LS180 (ATCC CL-187), LS 174 T (ATCC CL-138), Hepa 1-6 (ATCC CRL-1830) and LS1034 (ATCC CRL-2158) cells were obtained from ATCC (Manassas, VA). SNU-C4 (KCLB No. 0000C4) was obtained from Korean Cell Line Bank (Seoul, Korea). All cell lines have been authenticated by short tandem repeat DNA profiling, and they tested negative for mycoplasma contamination.

The curve-fitting software GraphPad Prism 4.0 (GraphPad Software, La Jolla, CA) was used to generate the dose–response curves and determine the IC_50_ and EC_50_ values. We performed statistical calculations by using one-way analysis of variance with Tukey’s multiple-comparison test or a paired *t*-test when comparing two groups. Sample sizes were chosen according to previous studies that showed statistical significance^27, 35, 47^, which also contained information on assay validation and determination of Z’ factor (a measure of statistical effect size)^47^. No data were excluded from the analyses.

### hPXR TR-FRET-binding assay

The TR-FRET-binding assays using BODIPY FL vindoline were performed by following a published protocol^47^ with minor modifications. Briefly, the test chemical, negative control (DMSO), or positive control (10 μM T0901317) were incubated with GST-hPXR-LBD (5 nM), Tb-anti-GST (5 nM), and BODIPY FL vindoline (100 nM), in 20 μl per well of assay buffer (50 mM Tris, pH 7.5, 20 mM MgCl_2_, 0.1 mg ml^−1^ BSA, 0.05 mM DTT) in a black 384-well low-volume assay plate, for 30 min. For all the assay wells, the final DMSO concentration was 1.1%. A PHERAstar FS plate reader (BMG LABTECH, Durham, NC) was used to detect the TR-FRET signals, by using the following instrumentation setting: a 340 nm excitation filter, a 100 μs delay time, and a 200 μs integration time. The TR-FRET ratio was expressed as 10,000 × 520 nm/490 nm. The activity of the positive (10 μM T0901317) and negative control (1.1% DMSO) was set as 100% and 0% inhibition, respectively. The activity of each assay well was then normalized to that of the positive and negative controls.

### Nuclear receptor transactivation assays

The hPXR transactivation assay using the HepG2 stable cell line (agonistic and antagonistic modes) were performed in the HepG2 cells stably expressing FLAG-hPXR and CYP3A4-luciferase reporter as previously described^27, 48^. Briefly, serially diluted hPXR agonist rifampicin (final DMSO concentration: 0.5%) and various concentrations of the test chemicals, either alone or in combination with 5 µM rifampicin, were added to the wells of white 384-well tissue culture-treated plates with 5000 cells in 25 µl of phenol red-free DMEM supplemented with 5% charcoal/dextran-treated FBS and incubated for 24 h at 37 °C before a luciferase assay was performed using steadylite HTS (PerkinElmer Life Sciences). The luminescence signal was detected with an EnVision plate reader (PerkinElmer Life Sciences). In the agonistic assays, DMSO (0.5% final concentration) was used as the negative control (0% activation) and rifampicin (10 µM in 0.5% DMSO) was used as the positive control (100% activation). In the antagonistic assays, rifampicin alone (5 µM with a final DMSO concentration of 0.5%) was used as the negative control (0% inhibition) and DMSO (0.5%) was used as the positive control (100% inhibition). The activities of individual chemicals tested at various concentrations were normalized to the corresponding positive and negative controls to generate the % activation in agonistic assays and the % inhibition in antagonistic assays.

The hPXR transactivation assay using transient transfection (agonist and antagonist modes) were performed as described previously^35^. Briefly, LS180 cells were co-transfected with pGL3-CYP3A4-luc (CYP3A4-luciferase reporter) and pRL-TK (as a transfection control), together with pcDNA3, pcDNA3-hPXR, pcDNA3-hPXRM425A, or pcDNA3-hPXRF429A, by using FuGENE 6 (Roche). The pcDNA3-hPXR mutants (pcDNA3-hPXRM425A and pcDNA3-hPXRF429A) were obtained from Codex BioSolutions, Inc. (Gaithersburg, MD). Mutations were verified through nucleotide sequencing, using primers shown in Supplementary Table 3. Twenty-four hours after the transfection, 15,000 cells per well were treated with DMSO or compounds in a 96-well culture plate (PerkinElmer) for 24 h in phenol red-free MEM (Thermo Fisher Scientific) supplemented with 5% charcoal/dextran-treated FBS before performing the Dual-Glo luciferase assay (Promega). The luminescence signal was detected with an EnVision plate reader (PerkinElmer).

For the mPXR transactivation assay, Hepa 1-6 cells were co-transfected with pCMV6-mPXR (Cat. No. MR226044, OriGene Technologies, Rockville, MD), pGL3-CYP3A4-luc and pRL-TK by using Lipofectamine 3000. Twenty-four hours after the transfection, 5000 cells per well were treated with SPA70 with or without 5 μM PCN in 384-well white culture plates (PerkinElmer) for 24 h in phenol red-free DMEM supplemented with 5% charcoal/dextran-treated FBS before performing the Dual-Glo luciferase assay (Promega). The relative luciferase activity was determined by normalizing the firefly luciferase signal to the *Renilla* luciferase signal. The final DMSO concentration was 0.3% in all assay wells. In the agonistic assays, 10 μM PCN and DMSO were used as the positive (100% activation) and negative control (0% activation), respectively. In the antagonistic assays, DMSO and 5 μM PCN were used as positive (100% inhibition) and negative (0% inhibition) controls, respectively. Compound activity was normalized to the controls.

The hCAR transactivation assays were performed as described previously^48^. Briefly, 5 million HepG2 cells were seeded into a T25 flask, allowed to grow for 24 h, then transiently transfected with a plasmid:FuGENE 6 mixture (0.75 µg of pcDNA3-FLAG-hCAR, 2.25 µg of CYP2B6-2.2 kb, and 0.3 µg of pRL-TK). Twenty-four hours after transfection, 5000 cells in 25 µl of phenol red-free DMEM supplemented with 5% charcoal/dextran-treated FBS were seeded into each well of 384-well white culture plates and treated with compounds for a further 24 h before a Dual-Glo luciferase assay was performed. The relative luciferase activity was determined by normalizing the firefly luciferase signal to the *Renilla* luciferase signal and was used to represent the ‘relative activity of CAR’. The final DMSO concentration was 0.4% in all assays. The activities of individual chemicals tested at various concentrations were normalized to that of the positive (1 μM CITCO for 100% activation in agonistic assays and 10 μM PK11195 for 100% inhibition in inverse agonistic assays) and negative (0.4% DMSO for both the agonistic and inverse agonistic assays) controls to generate the % activation in agonistic assays and the % inhibition in inverse agonistic assays.

For the GeneBLAzer NR-UAS-bla HEK 293 T assays for VDR, RXRα, RXRβ, FXR, GR, LXRα, LXRβ and PPARγ, GeneBLAzer panel nuclear receptor cells, the LiveBLAzer FRET-B/G Loading Kit with CCF4-AM, and all tissue culture reagents were purchased from Thermo Fisher Scientific. The assays were performed in accordance with the manufacturer’s instructions, with minor modifications as reported previously^48–50^. Briefly, positive control (as described for each assay), DMSO, and dilutions of test chemicals either alone (for agonistic assays) or in combination with an approximately EC_80_ concentration of a specific agonist (for antagonistic assays) were incubated with an optimized number of cells for each assay in wells of black 384-well tissue culture-treated clear-bottom plates with 30 µl of the respective assay medium in a 37 °C cell-culture incubator. A group of wells containing assay medium and DMSO, but no cells, served as a background control. The final DMSO concentration was 0.5% for all wells. After 24 h, 6 µl per well of loading solution was added, and this step was followed by an optimal period of incubation at room temperature in the dark before the fluorescent emission at 460 and 535 nm (using excitation at 400 nm) was measured with an EnVision plate reader. After the background was subtracted, the emission signals at 460 and 535 nm were used to determine the 460 nm/535 nm ratio and to represent the activity of the tested chemical. The reference agonists used included 1α,25-dihydroxyvitamin D3 for VDR (2 nM for 0% inhibition (antagonistic assay) or 100 nM for 100% activation (agonistic assay)), 9-*cis*-retinoic acid for RXRα and RXRβ (100 nM for 0% inhibition (antagonistic assay) or 10 μM for 100% activation (agonistic assay)), GW4604 for FXR (200 nM for 0% inhibition (antagonistic assay) or 10 µM for 100% activation (agonistic assay)), dexamethasone for GR (5 nM for 0% inhibition (antagonistic assay) or 100 nM for 100% activation (agonistic assay)), T0901317 for LXRα (300 nM for 0% inhibition (antagonistic assay) or 2 µM for 100% activation (agonistic assay)), T0901317 for LXRβ (3 µM for 0% inhibition (antagonistic assay) or 20 µM for 100% activation (agonistic assay)), and rosiglitazone for PPAR γ (100 nM for 0% inhibition (antagonistic assay) or 2.5 µM for 100% activation (agonistic assay)). DMSO was used as a 0% activation control for all agonistic assays and as a 100% inhibition control in the antagonistic assays for VDR, RXRα, RXRβ, FXR, LXRα and LXRβ. For GR and PPARγ antagonistic assays, 5 nM dexamethasone in the presence of 100 nM mifepristone and 100 nM rosiglitazone in the presence of 1 µM GW9662 were used as the respective 100% inhibition controls.

### Cytotoxicity assays

For the HepG2, HEK293 and Hepa 1-6 cytotoxicity assays, serially diluted SPA70, staurosporine (56 µM), or DMSO (0.5%) were added to the wells of white 384-well tissue culture-treated plates containing HepG2 (5000 cells for 1-day cytotoxicity or 2,500 cells for 3-day cytotoxicity), HEK293 (20,000 cells for 1-day cytotoxicity or 2,500 cells for 3-day cytotoxicity) or Hepa 1-6 (5,000 cells for 1-day cytotoxicity or 2,500 cells for 3-day cytotoxicity) in 25 µl of phenol red-free DMEM supplemented with 5% charcoal/dextran-treated FBS (for the 1-day assay) or DMEM supplemented with 10% FBS (for the 3-day assay). After the plated had been incubated for 24 or 72 h at 37 °C, the cytotoxicity was determined using a CellTiter-Glo luminescent cell viability assay (Promega). The luminescence signal was detected using an EnVision plate reader. The final DMSO concentration was 0.5% for all wells. DMSO (0.5%) was used as the negative control (0% cytotoxicity) and staurosporine (56 µM with 0.5% DMSO) was used as the positive control (100% cytotoxicity). The activities of SPA70 tested at various concentrations were normalized to the positive and negative controls to generate the % cytotoxicity.

For the LS180, SNU-C4 and LS1034 cytotoxicity assays, cells were seeded in a 96-well plate (Corning) at 4000 cells per well. They were then treated with paclitaxel, vincristine, or vinblastine in quadruplicate at serially diluted concentrations in the presence or absence of 10 μM SPA70 for 72 h at 37 °C. Cell viability was assessed using the CellTiter-Glo assay. Each experiment was repeated at least three times. siRNAs targeting PXR (SMARTpool, M-003415-02; or individual siRNAs D-003415-02, D-003415-03, D-003415-04 and D-003415-05) or nontargeting control siRNA (D-001210-03) (GE Healthcare Dharmacon, Lafayette, CO) were reverse transfected into 2,500 cells in each well of a 96-well plate by using Lipofectamine RNAiMAX (Life Technologies, Carlsbad, CA) at a final concentration of 25 nM. After 48 h of transfection, the cells were further treated with either DMSO or SPA70, and with paclitaxel, vincristine, or vinblastine.

### Western blot analysis

Cells were rinsed once with cold PBS then lysed in RIPA lysis buffer (Thermo Fisher Scientific) containing protease inhibitor (Roche). Mouse liver samples were homogenized in 500 μl of cold RIPA lysis buffer in a Bullet Blender Blue homogenizer (Next Advance, Averill Park, NY). Then, 25 μg of total protein lysates were loaded onto NuPAGE 4%–12% Bis-Tris gels (Invitrogen) with NuPAGE MES SDS running buffer (Invitrogen). The proteins were transferred from the gel to a nitrocellulose membrane by using the iBlot gel-transfer system (Invitrogen). The membrane was then blocked for 1 h with Odyssey Blocking Buffer (LI-COR Biosciences, Lincoln, NE) and probed with mouse monoclonal antibodies against CYP3A4 (K03; stock concentration: 2.5 mg ml^−1^, diluted 1:5000)^51^, MDR1 (D-11; Cat. No. sc-55510, Santa Cruz Biotechnology, Santa Cruz, CA; stock concentration: 0.2 mg ml^−1^, diluted 1:1500), hPXR (H-11; Cat. No. sc-48340, Santa Cruz Biotechnology; stock concentration: 0.2 mg ml^−1^, diluted 1:400), mouse Cyp3a (Cat. No. MAB10041, Millipore, Temecula, CA; Lot No. 1957980, diluted 1:1000), or β-actin (Cat. No. A5441, Sigma; Lot No. 043M4840V, diluted 1:5000). The membranes were then incubated with secondary goat anti-mouse antibody labeled with infrared dye (IRDye® 800CW Goat anti-Mouse IgG [H + L], Cat. No. 926-32210, stock concentration: 1 mg ml^−1^, diluted 1:15,000; or IRDye® 680LT Goat anti-Mouse IgG [H + L], Cat. No. 926-68020, stock concentration: 1 mg ml^−1^, diluted 1:20,000) (LI-COR Biosciences). An Odyssey infrared imager (LI-COR Biosciences) was used to visualize the protein bands. The relative intensity of each protein band was then determined by normalizing the intensity of each protein band to that of actin. The relative intensity of the sample treated with vehicle control (DMSO) was set as 1.0. At least two independent experiments were performed, and a representative gel is shown.

### Microarray analysis

Primary human hepatocytes (#HH1811) grown in Williams’ Medium E containing Hepatocyte Maintenance Supplement Pack (Thermo Fisher Scientific) at 37 °C in 5% CO_2_ were treated with vehicle control (DMSO), 1 μM rifampicin, 100 nM SR12813 or 30 nM T090137, with or without co-treatment with 2.5 μM SPA70, for 48 h in six-well plates. In each case, total RNA was extracted using a Qiagen RNeasy Mini Kit and amplified and labeled by using an One-Color Low Input Quick Amp Labeling Kit (Agilent, Santa Clara, CA, catalog no. 5190-2305), after which it was hybridized to the SurePrint G3 Human GE 8 × 60 K microarray (Agilent-028004), which contains 42,545 unique probes targeting 27,958 Entrez genes. Microarrays were scanned with an Agilent array scanner (G2565CA) at 3-μm resolution. Microarray data were extracted by Agilent Feature Extraction software (v10.5.1.1). Quantile normalization of log-transformed, background-subtracted signal intensity was performed for all samples. This was followed by comparisons between sets of replicates from different experimental groups. Student’s *t*-test was used to determine the statistical significance of the difference between the paired samples from three replicates of each experiment. The expression of a gene was considered significantly different if the *P*-value was less than 0.05 and the expression change was more than twofold in at least one of the group comparisons. The data processing and PCA analysis were performed using Partek software (St. Louis, MO)^52^. The GEO accession number for the microarray analysis is GSE90122.

### LC-MS/MS analysis

To examine the metabolism of midazolam (MDZ), and paclitaxel (PTX), a 100-μL sample of the reaction medium was collected at each time point, and 200 μl of acetonitrile containing midazolam-D4 maleate (the internal standard [IS]) was added. This solution was then vigorously mixed for 10 min and centrifuged at 9633 *g* for 10 min. Calibration and quality-control samples were prepared. For each sample, 5 μl was injected onto an Acquity UPLC BEH C18 column (2.1 × 50 mm, 1.7 µm) (Waters Corporation, Milford, MA) by using an Acquity UPLC system (Waters Corporation). Chromatographic separation was performed by gradient elution at a constant flow rate of either 1 ml min^−1^ for 15 min (in the MDZ experiment) or 0.8 ml min^−1^ for 3 min (in the PTX experiment). The gradient consisted of 0.1% formic acid–water (mobile phase A) and methanol (mobile phase B). In the MDZ experiment, the gradient applied was 0.0 min, 90% A–10% B; 1.35 min, 80% A–20% B; 1.65 min, 5% A–95% B; and 1.95 min, 10% A–90% B. In the PTX experiment, the gradient applied was 0.0 min, 90% A–10% B; 0.2 min, 70% A–30% B; 2.40 min, 5% A–95% B; and 3 min, 10% A–90% B. The eluate was directed to a SCIEX Triple Quad™ 6500 System mass spectrometer (Applied Biosystems SCIEX, Foster, CA) equipped with an electrospray ionization source. Mass transitions of *m*/*z* 326 to *m*/*z* 291 for MDZ, *m*/*z* 342 to *m*/*z* 324 for 1-OH MDZ, *m*/*z* 330 to *m*/*z* 295 for the IS, *m*/*z* 876 to *m*/*z* 308 for PTX, *m*/*z* 892 to *m*/*z* 324 for 3′-p-hydroxypaclitaxel, and *m*/*z* 892 to *m*/*z* 308 for 6α-hydroxypaclitaxel were monitored. Ionization was achieved at 3 kV and a temperature of 650 °C and 550 °C for the MDZ and PTX experiment, respectively. Nitrogen was applied as a curtain, collision, and drying gas at 60 p.s.i. The declustering potentials, entrance potential, and collision energy were as follows: 120, 12 and 35 V for MDZ; 70, 12 and 30 V for 1-OH MDZ; 120, 12 and 37 V for the IS; 242, 37 and 32 V for PTX; 281, 37 and 36 V for 3′-p-hydroxypaclitaxel; and 266, 38 and 38 V for 6α-hydroxypaclitaxel.

### RNA extraction and RT-PCR

The method used for RNA extraction and polymerase chain reaction with reverse transcription (RT-PCR) has been described previously^35^. Briefly, total RNA was isolated from cells or mouse liver tissue by using the Maxwell 16 LEV simplyRNA purification kit (Promega). Quantitative RT-PCR was performed in accordance with the manufacturer’s protocol by using TaqMan gene expression assays (Thermo Fisher Scientific) specific for the *hPXR* and *Cyp3a11* genes, with *GAPDH* or 18s rRNA as the reference gene, in an ABI 7900HT Fast Real-Time PCR System (Thermo Fisher Scientific). The primers used are listed in Supplementary Table 3. The comparative threshold (Ct) method was used for the relative quantification of gene expression by the following formulae: ΔCt = Ct (test gene) − Ct (GAPDH); ΔΔCt (test gene) = ΔCt (test gene in treatment group) − ΔCt (test gene in vehicle control group). The fold change of the mRNA is 2^–ΔΔCt^, which indicates the mRNA level of the corresponding transcript in relation to that in the control samples.

### In vivo toxicity and drug efficacy studies

We performed all animal experiments in accordance with a protocol approved by the St. Jude Children’s Research Hospital Institutional Animal Care and Use Committee. Male C57BL/6 mice (8–15 weeks old) (Charles River Laboratories, Wilmington, MA) and humanized PXR (*hPXR*-tg) mice^14^ were housed with free access to food and water in a room at 22–23 °C with a 12 h light/dark cycle in the St. Jude Animal Resources Center, which is certified by the American Association for Accreditation of Laboratory Animal Care. All animals used in a given experiment were matched for age and body weight. The investigator was not blinded to the group allocation during the experiment or when assessing the outcome.

In the animal acute toxicity studies, C57BL/6 mice were randomly apportioned among six groups, with five mice per group. Each mouse received saline containing 7.5% polyethylene glycol 400 (PEG) (as vehicle control) or 250 or 500 mg kg^−1^ of SPA70, in each case administered as a single dose by intraperitoneal (i.p.) injection or orally. After dosing, mice were monitored daily for 15 days for morbidity and mortality. Signs of morbidity included ruffled fur, hunching, tremors, lethargy, hyperactivity and diarrhea, in addition to a reduction in weight gain. The body weight of each mouse was recorded before and after treatment. At day 15, all mice were killed and a terminal blood sample was collected for examination of clinical chemistries (e.g., renal, liver and hematopoietic function). Histopathological examination of the major organs was performed with hematoxylin and eosin staining.

For the hydrodynamic injections, treatment, and in vivo imaging, in vivo delivery of the *CYP3A4-luc* reporter gene and the *hPXR* gene into mouse liver was performed using the hydrodynamic injection method described previously^34, 53, 54^. Briefly, by a rapid (5–10 s) tail-vein injection, each mouse received 25 μg of linearized CYP3A4-luc plasmid DNA with linearized hPXR plasmid DNA in sterile saline in a volume equal to 10% of its body weight. Imaging for luciferase activity was performed 2–8 weeks after somatic gene transfer (the bioluminescence was stable in the mice during the 2–8 weeks period). Fifteen mice were given i.p. injections of VivoGlo luciferin (Promega) (150 mg kg^−1^ of body weight), anesthetized with 2.5% isoflurane, and imaged 10 min later in a Xenogen IVIS 200 system (Xenogen) to obtain a basal image. The mice then underwent three rounds of sequential treatment, each consisting of i.p. injection of vehicle control, 10 mg kg^−1^ rifampicin or 10 mg kg^−1^ rifampicin plus 150 mg kg^−1^ SPA70 every 24 h for 2 days, with a washout period of 72 h between rounds of treatment. The mice were imaged 10 h after the last treatment in each round. The Living Image 3.2 software (Xenogen) was used to analyze the total photon flux of uniform regions of interest drawn around the liver. The CYP3A4-luc reporter activity (expressed as induction rate) was determined by using the total photon flux from the same mouse before and after treatment: induction rate = total photon flux (after treatment)/total photon flux (before treatment).

*hPXR*-tg mice were generated previously^14^. Three to five mice in each group were dosed orally with vehicle control, 10 mg kg^−1^ rifampicin, or 10 mg kg^−1^ rifampicin plus 200 mg kg^−1^ SPA70 (administered by i.p. injection) every 24 h for 3 days. Eight hours after the last dose, the animals were killed with CO_2_ and their liver tissues were harvested. A piece of each liver was preserved in RNAlater solution (Invitrogen) at 4 °C for subsequent mRNA isolation. The remaining tissue was instantly frozen in liquid nitrogen and stored at −80 °C for subsequent total protein extraction.

The LORR assay method has been described previously^35^, by using 2,2,2-tribromoethanol, a substrate of mouse Cyp3a11 which is induced by hPXR. Mice were first injected intraperitoneally with 250 mg kg^−1^ of 2,2,2-tribromoethanol, then placed on their backs under a heat lamp as soon as they lost their righting reflex. The LORR duration was defined as the time period from the start of LORR to when mice could right themselves twice within 1 min, after being placed on their backs. For each mouse, a baseline LORR duration was first established before oral administration of vehicle or compounds. After a 1-week washout period, each mouse underwent oral administration of vehicle, 10 mg kg^−1^ rifampicin or 10 mg kg^−1^ rifampicin plus 200 mg kg^−1^ SPA70 (administered by i.p. injection) every 24 h for 3 days, and the righting reflex experiment was repeated at least 8 h after the last treatment. A paired Student’s *t*-test was used to compare the LORR duration at baseline and after treatment. A *P*-value of less than 0.05 was considered to indicate a significant difference between the compared groups.

### Mammalian two-hybrid assay

The mammalian two-hybrid assay method has been described previously^35^. Briefly, the CheckMate mammalian two-hybrid system (Promega) was used to evaluate the association between hPXR and its co-regulators. LS180 cells were co-transfected with an hPXR plasmid (pACT-hPXR, pACT-hPXRM429A or pACT-hPXRF429A), a coregulator plasmid (pBIND-SRC-1, pBIND-TIF2, pBIND-SMRTτ [amino acids 2077–2471], or pBIND-mNCoR [amino acids 1958–2401]), and a GAL4 firefly luciferase reporter plasmid (pG5-luc). The *Renilla* luciferase is constitutively expressed from the pBIND vector, and served as an internal transfection control. The activity of the firefly and *Renilla* luciferase was measured by using the Dual-Glo Luciferase Assay (Promega), and the relative luciferase activity of pG5-luc was determined by normalizing the activity of firefly luciferase to that of *Renilla* luciferase.

### PXR-cofactor interaction TR-FRET assay

FAM-(PEG)_5_-CPSSHSSLTERHKILHRLLQEGSPS-NH_2_ (F-SRC1) was chemically synthesized by the Hartwell Center at St. Jude Children’s Research Hospital (Memphis, TN). A similar procedure for the nuclear receptor–cofactor interaction TR-FRET assay has been described previously^44^. Briefly, dilutions of chemicals or DMSO were incubated with 5 nM GST-hPXR-LBD, 5 nM Tb–anti-GST and 500 nM F-NCoRID2 (for the corepressor recruitment assay) or 100 nM F-SRC1 (for the coactivator recruitment assay) for 120 min in 20 μl per well assay buffer (50 mM Tris, pH 7.5, 20 mM MgCl_2_, 0.1 mg ml^−1^ BSA, 0.05 mM DTT) in black 384-well low-volume assay plates. The TR-FRET signals (10,000 × 520 nm/490 nm) were then measured using a PHERAstar FS plate reader. The final DMSO concentration was 1.1% for all assay wells.

### Protein purification and preparation

The cDNA fragments of the hPXR LBD (residues 130–434) and mouse SRC-1 (residues 623–710) were synthesized, and the *Escherichia coli*–preferred codon was used (Supplementary Table 4). To express the hPXR LBD and SRC-1 from the same expression vector (for crystallization), the cDNA fragments of the hPXR LBD and SRC-1 were inserted into the pETDuet1 plasmid at *Nco*I/*Hin*dIII and *Nde*I/*Kpn*I sites, respectively. To express only the hPXR LBD (for HDX analysis), the hPXR LBD cDNA fragment was subcloned into pETDuet1 at *Nco*I/*Hin*dIII sites. The recombinant plasmid was then used to transform *E. coli* BL21(DE3). *E. coli* cells were grown in Luria broth cell-culture medium with 100 mg l^−1^ ampicillin and induced at an OD600 of 0.6 by using 0.8 mM IPTG (Isopropyl β-D-thiogalactoside) overnight at 18 °C. The cells were then harvested by centrifugation at 4000 *g* at 4 °C, resuspended in Buffer S (50 mM Tris-HCl, pH 7.5, 150 mM NaCl, 10% glycerol) containing EDTA-free protein inhibitor cocktail (Roche, catalog no. 04693132001) (10 ml buffer per gram of cells), and sonicated on ice. The supernatant was collected and loaded onto a Ni-NTA/XK 26/20 column packed with 20 ml of beads (Qiagen, catalog no. 30310). The column was washed sequentially with Buffer S containing 0, 50, 100 and 200 mM imidazole, and each fraction from the Ni-NTA column was analyzed by sodium dodecyl sulfate-polyacrylamide gel electrophoresis (SDS-PAGE). The 200 mM imidazole-eluted fraction was collected and diluted (1:2) in Buffer S. For crystallization, the diluted protein sample was incubated with 1.5-fold molar SRC-1 peptide (CPSSHSSLTERHKILHRLLQEGSPS) overnight at 4 °C to minimize hPXR LBD precipitation. After this incubation, any precipitate was removed by centrifugation, and the sample was filtered with a 0.45 μm membrane (Millipore, catalog no. SLHP033RS). The clarified sample was then loaded onto a MonoS column (1 ml) (GE, catalog no. 17-5168-01) pre-equilibrated with Buffer A (20 mM Tris-HCl, pH 7.5, 50 mM NaCl, 5% glycerol, 5 mM DTT). The column was washed with the following gradient: 0%–50% Buffer B (20 mM Tris-HCl, pH 7.5, 1 M NaCl, 5% glycerol, 5 mM DTT), 25 CV; 50%–100% Buffer B, 10 CV; and 1 M NaOH. The fractions containing purified hPXR LBD protein were pooled, and the buffer was exchanged to 20 mM Tris-HCl, pH 7.8, 250 mM NaCl, 5% glycerol, 5 mM DTT, and 2.5 mM EDTA with a HiPrep™26/10 Desalting Column (GE, catalog no. 17-5087-01). The protein sample was then concentrated to 9.1 mg ml^−1^ by using centrifugal filter units (10 K MWCO; Millipore). The purity of the concentrated protein was assessed by SDS-PAGE.

### X-ray crystallography

For crystallization, compound SJB7 was mixed with purified hPXR LBD/SRC-1 at a 5:1 molar ratio and incubated for at least 4 h at 4 °C. Co-crystallization of hPXR LBD/SRC-1 with compound SJB7 was set up by using the hanging-drop vapor diffusion method at 20 °C^23^. The SJB7–hPXR LBD/SRC-1 complex was crystallized against 50 mM imidazole (pH 7.8) and 10% 2-propanol (v/v). Crystals were cryoprotected by sequentially dipping them in 15, 25 and 35% ethylene glycol and then flash-frozen in liquid nitrogen.

For crystallization data collection, structure determination and refinement, diffraction data were collected (wavelength: 0.97923 Å; temperature: 100 K) over 180° in 1° increments at the Shanghai Synchrotron 17 U beamline. Diffraction data were indexed, integrated, and scaled using HKL2000 software^55^. The hPXR LBD/SRC-1 co-crystal with compound SJB7 was collected at a resolution of 2.66 Å. The crystal belongs to space group P2_1_2_1_2_1_, with cell parameters of *a* = 84.34 Å, *b* = 89.30 Å, *c* = 106.76 Å, *α* = *β* = *γ* = 90°. The data quality is summarized in Table 1. The structure of the hPXR LBD/SRC-1–SJB7 complex was determined by molecular replacement with the MolRep module of the CCP4 suite, using the structure of 1NRL (Protein Data Bank ID) as the searching model^56, 57^. Two protein molecules (each in complex with the SRC-1 peptide) were observed in the asymmetric unit. The structure was refined with the Refmac5 program^56^. The final model exhibits good geometry, as indicated by the Ramachandran plot (residues in the preferred region, 96%; allowed region, 3.8%; outliers 0.4%).

### Docking of SPA70 to the hPXR LBD

Docking of SPA70 to the hPXR LBD was conducted in a similar manner to that described elsewhere^58^ and was performed using AutoDock Vina version 1.1.1^59^. All water and ligand molecules were removed from the crystal structure by using the Pymol molecular graphics system (http://www.pymol.org) before the protein and ligand PDBQT files needed for docking were generated using AutoDockTools (ADT) version 1.5.6 (http://mgltools.scripps.edu/).

### HDX detected by MS

Differential HDX-MS experiments were conducted as previously described with a few modifications^41, 60^.

Peptides were identified using tandem MS (MS/MS) with an Orbitrap mass spectrometer (Q Exactive, Thermo Fisher Scientific). Product ion spectra were acquired in a data-dependent mode with the top five most abundant ions selected for the product ion analysis per scan event. The MS/MS data files were submitted to Mascot (Matrix Science) for peptide identification. Peptides included in the HDX analysis peptide set had a MASCOT score greater than 20 and the MS/MS spectra were verified by manual inspection. The MASCOT search was repeated against a decoy (reverse) sequence and ambiguous identifications were ruled out and not included in the HDX peptide set.

For the HDX-MS analysis, hPXR LBD (0.1 mg ml^−1^ in 20 mM Tris-HCl, pH 7.8, 250 mM NaCl, 5 mM DTT) was incubated with the respective ligands at a 1:10 molar ratio (protein:ligand) for 1 h or further incubated with the cofactor peptide at a 1:10 molar ratio (protein complex:cofactor peptide) for 1 h before the HDX reactions. Either SRC-1 (CPSSHSSLTERHKILHRLLQEGSPS) or NCoR1-3 (ASNLGLEDIIRKALMGSFD) cofactor peptide was used as indicated. A 5 µl sample of protein or protein complex with ligand/peptide was diluted in 20 µl D_2_O on-exchange buffer (20 mM Tris-HCl, pH 7.8, 250 mM NaCl, 5 mM DTT), incubated for various HDX time points (e.g., 0, 10, 60, 300 and 900 s) at room temperature, then quenched by mixing with 25 µl of ice-cold 3 M urea, 1% trifluoroacetic acid. The sample tubes were immediately placed on dry ice after the quenching reactions until the samples were injected into the HDX platform. The protein was then passed through an immobilized pepsin column (2 mm × 2 cm) at 200 μL min^−1^, and the digested peptides were captured on a 2 mm × 1 cm C_8_ trap column (Agilent) and desalted. Peptides were separated across a 2.1 mm × 5 cm C_18_ column (1.9 μl Hypersil Gold; Thermo Fisher Scientific) with a linear gradient of 4–40% CH_3_CN, 0.3% formic acid, over 5 min. Protein digestion and peptide separation were conducted at 4 °C. Mass spectrometric data were acquired using an Orbitrap mass spectrometer (Q Exactive, Thermo Fisher Scientific) with a measured resolving power of 65,000 at *m*/*z* 400. HDX analyses were performed in triplicate, with single preparations of each protein–ligand complex. The intensity-weighted mean centroid *m*/*z* value of each of the peptide envelopes was calculated and subsequently converted into a percentage of deuterium incorporation. Statistical significance for the differential HDX data was determined by an unpaired *t*-test for each time point, a procedure that is integrated into the HDX Workbench software^61^. The deuterium level was calculated, and corrections for back-exchange were made on the basis of an estimated 70% deuterium recovery, accounting for the known 80% deuterium content of the deuterium exchange buffer.

For data rendering, the HDX data from all overlapping peptides were consolidated to individual amino acid values using a residue averaging approach, in a similar way as previously described^62^. Briefly, for each residue, the deuterium incorporation values and peptide lengths from all overlapping peptides were assembled, and a weighting function was applied in which shorter peptides were weighted more heavily and longer peptides were weighted less. Each of the weighted deuterium incorporation values was then averaged to produce a single value for each amino acid. The initial two residues of each peptide, as well as proline residues, were omitted from the calculations.

### Data availability

The authors declare that all data supporting the findings of this study are available within the article and its Supplementary Information or from the corresponding author upon reasonable request. The accession codes are as follows: 5X0R for hPXR LBD with SJB7 bound (Protein Data Bank); GSE90122 for the microarray analysis (GEO accession number).

## Electronic supplementary material

Supplementary Information

Supplementary Description

Supplementary Data 1
