# Supplementary material for: SPA70 is a potent antagonist of human pregnane X receptor
Source: Nat Commun. 2017 Sep 29;8:741. doi: 10.1038/s41467-017-00780-5 (PMC5622171; doi:10.1038/s41467-017-00780-5)
Supplement: Supplementary file 1 — Supplementary Information [file 41467_2017_780_MOESM1_ESM.pdf]

<u>**Supplementary Figures**</u>

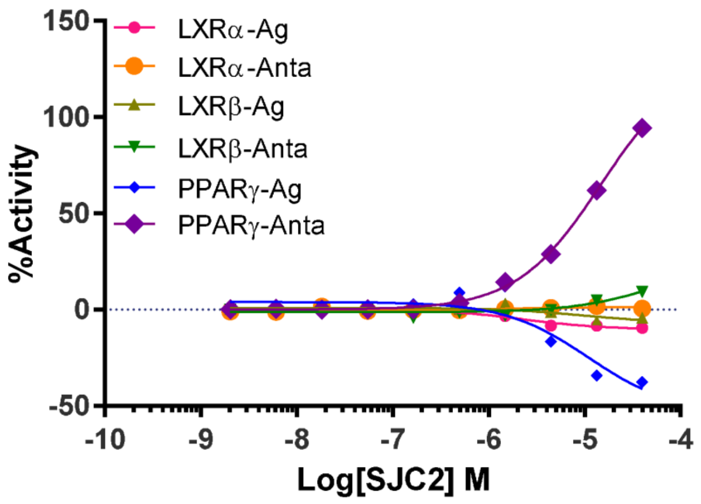

Supplementary Figure 1. SJC2 moderately antagonizes PPARγ. The assays were performed as described for Figure 1b,c.

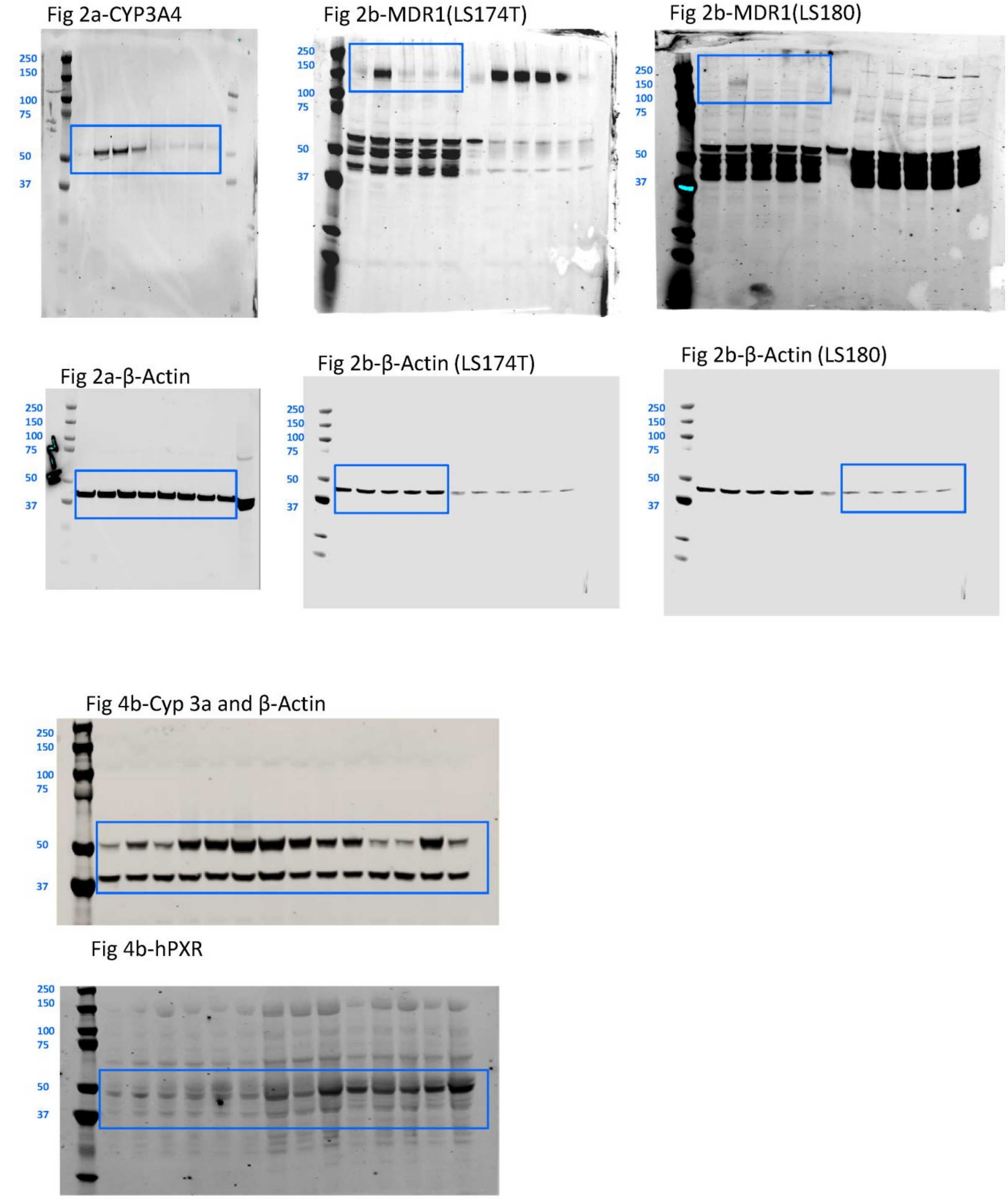

Supplementary Figure 2. Full gel images for Figures 2 and 4.

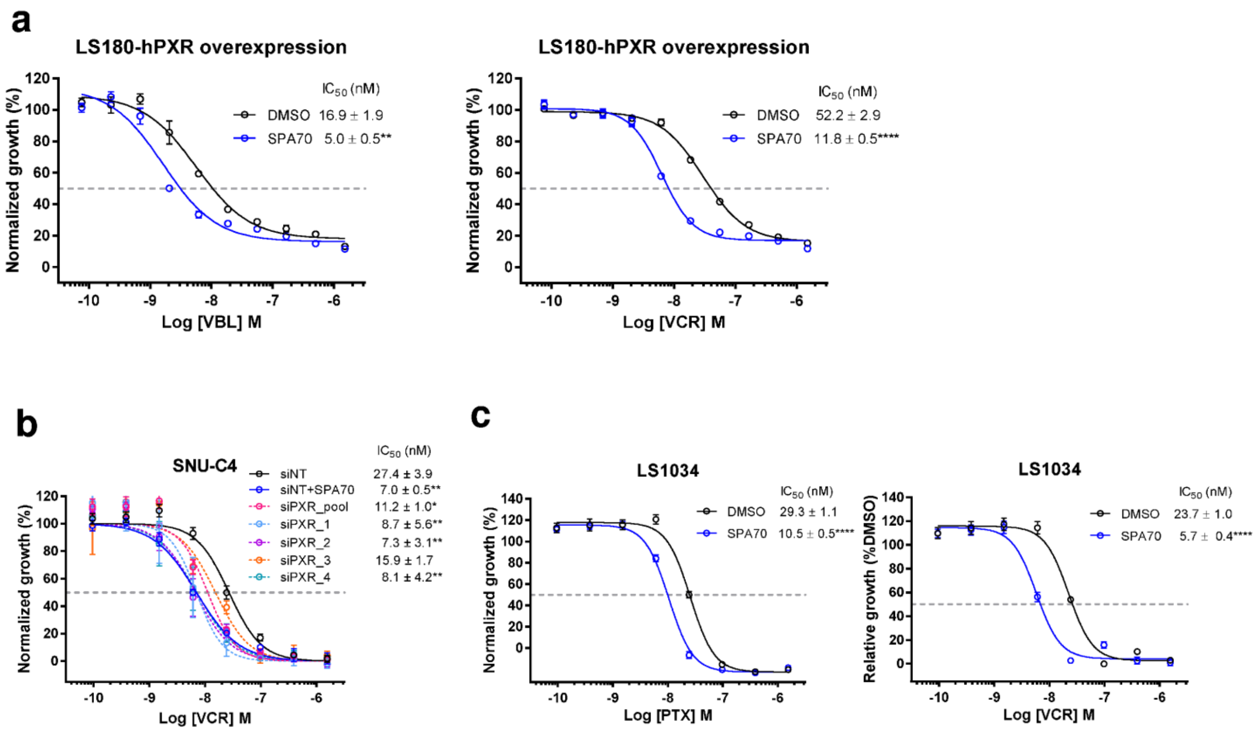

Supplementary Figure 3. SPA70 sensitizes cancer cells to chemotherapeutic agents. LS180 cells with stable overexpression of hPXR (**a**), SNU-C4 with or without siPXR (**b**), and LS1034 cells (**c**) were treated with serially diluted compounds (vinblastine: VBL; vincristine: VCR, paclitaxel: PTX) and either 0.1% DMSO or 10 µM SPA70 for 96 h before a CellTiter-Glo assay was performed. Data are presented as described in the legend for Figure 3. Final DMSO concentration was 0.2% in (a) - (c). $*P < 0.05$, $**P < 0.01$ or $****P < 0.0001$ for SPA70-treated cells compared to DMSO-treated cells by using a *t*-test (a and c) or for each group compared to siNT-treated cells by using one-way ANOVA with Tukey's multiple-comparison test (b).

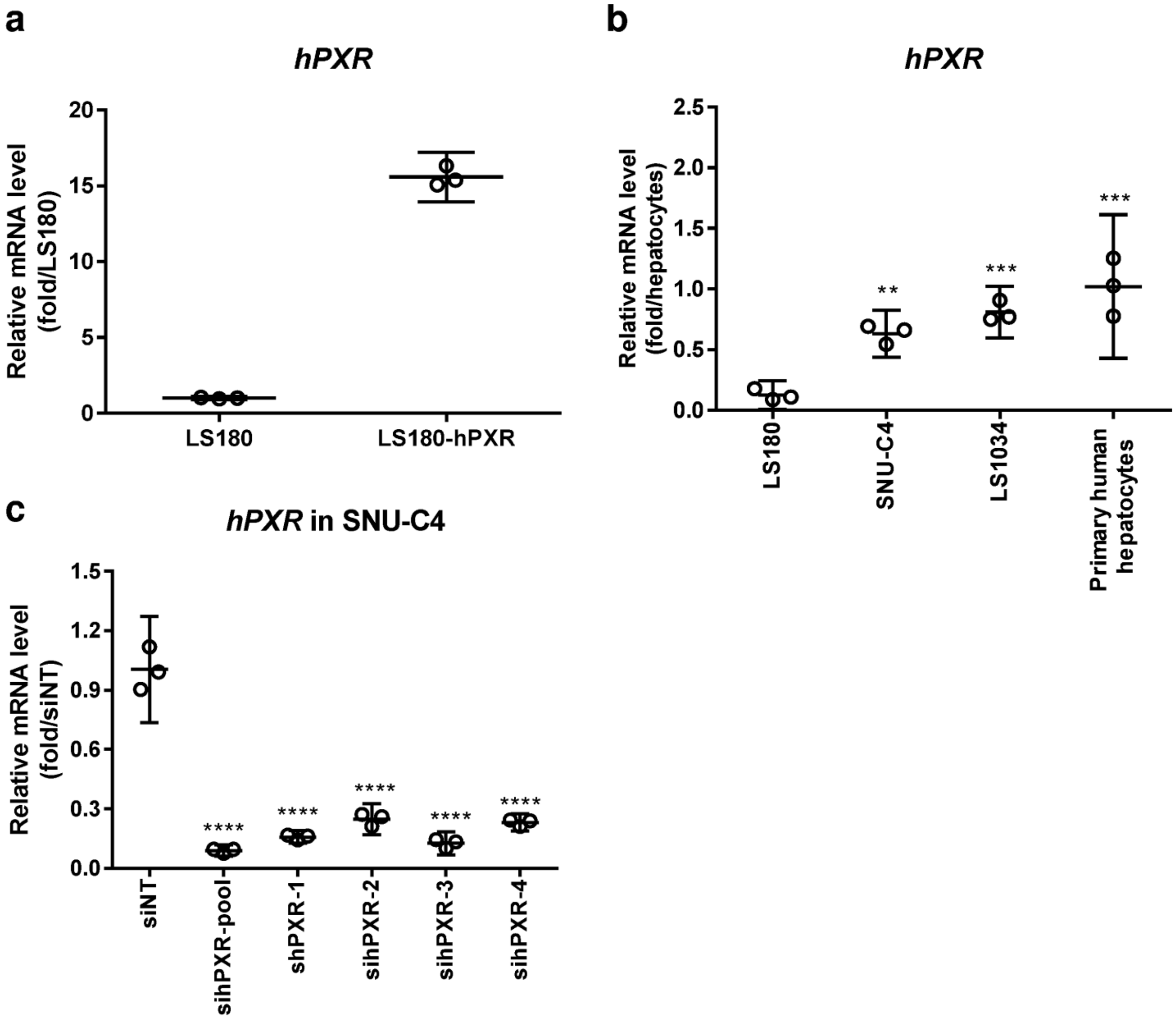

Supplementary Figure 4. Expression levels of hPXR in various cell models. *hPXR* mRNA levels were analyzed by real-time PCR in LS180 and LS180 cells with exogenously expressed hPXR (LS180-hPXR) (**a**); in LS180, SNU-C4, and LS1034 colon cancer cells and in primary human hepatocytes (**b**); and in SNU-C4 cells with either siNT or siPXR (individual or pooled as indicated) (**c**). Each data point represents the level of hPXR mRNA in an individual experiment. Data are expressed as the mean and 95% CI from three independent experiments.

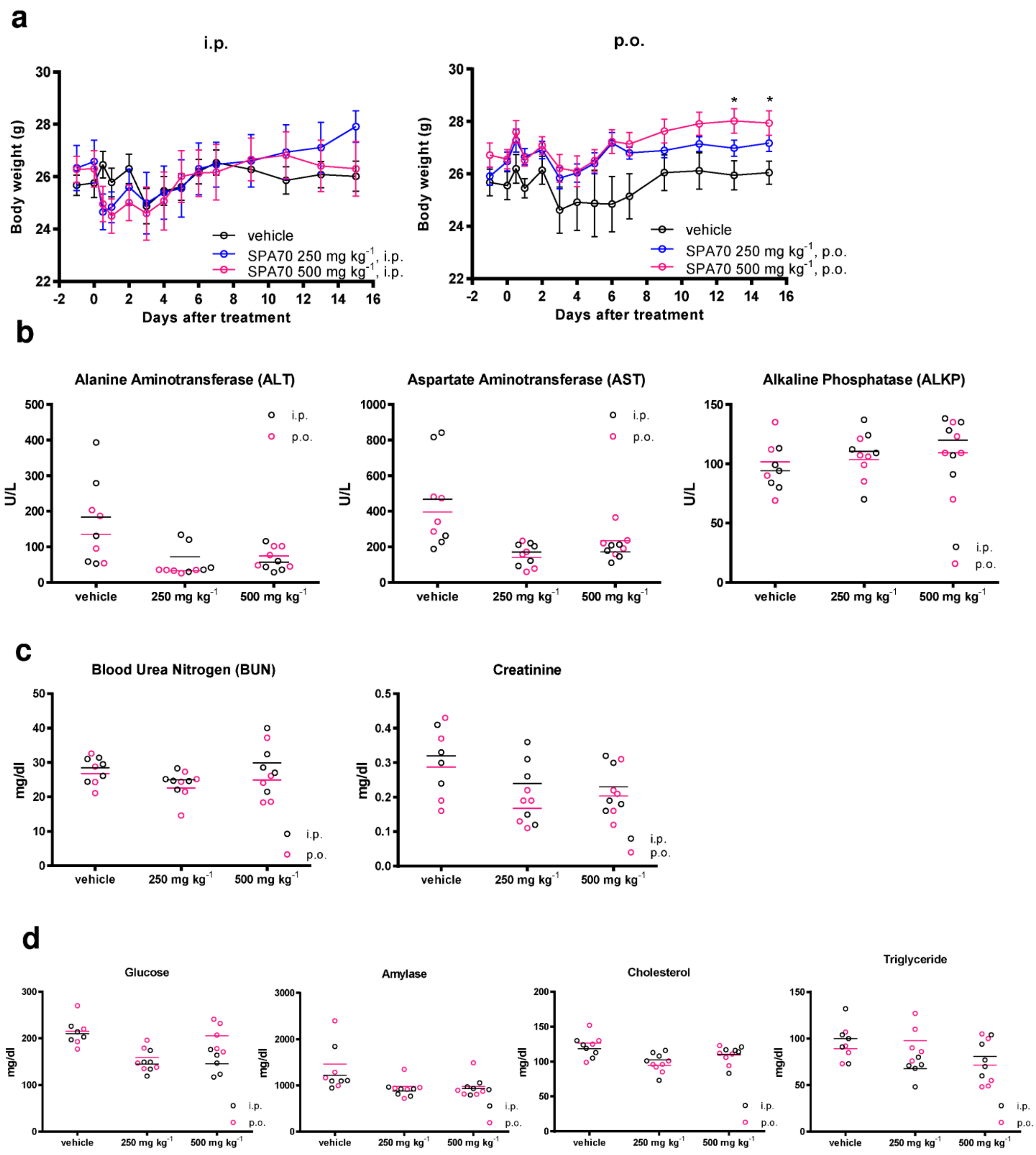
a
i.p.
p.o.
Body weight (g)
Days after treatment
vehicle
SPA70 250 mg kg-1, i.p.
SPA70 500 mg kg-1, i.p.
SPA70 250 mg kg-1, p.o.
SPA70 500 mg kg-1, p.o.
b
Alanine Aminotransferase (ALT)
Aspartate Aminotransferase (AST)
Alkaline Phosphatase (ALKP)
U/L
vehicle
250 mg kg-1
500 mg kg-1
i.p.
p.o.
c
Blood Urea Nitrogen (BUN)
Creatinine
mg/dl
d
Glucose
Amylase
Cholesterol
Triglyceride

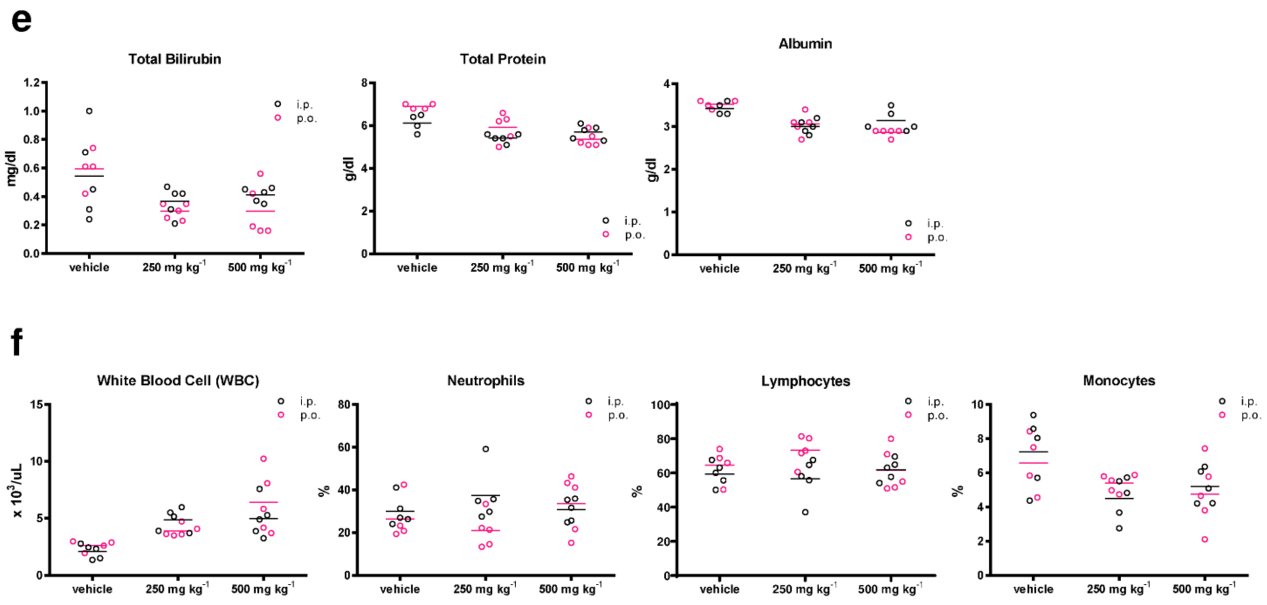

Supplementary Figure 5. SPA70 does not cause detrimental health effects in mice at doses of 250 or 500 mg kg^{-1}. (**a**) In the acute toxicity experiment in healthy mice, the body weight of the animals was monitored daily during SPA70 treatment (by intraperitoneal [i.p.] injection, left panel, or oral administration [p.o.], right panel). Data represent the mean ± SEM for five mice in each treatment group. *P < 0.05 for the SPA70-treated group compared to the vehicle-treated group by one-way ANOVA with Tukey's multiple-comparison test. (**b**) Liver-function profile (alanine transaminase [ALT]; aspartate transaminase [AST]; and alkaline phosphatase [ALKP]) after the indicated treatments. (**c**) Renal-function profile (blood urea nitrogen [BUN] and creatinine) after the indicated treatments. (**d**) Glucose and lipid (amylase, cholesterol, and triglyceride) profile after the indicated treatments. (**e**) Profile of total bilirubin, total serum protein, and albumin after the indicated treatments. (**f**) Complete blood count after the indicated treatments. Each data point represents the level of the measured variable in an individual mouse, and the lines indicate the mean for each treatment group (i.p. or p.o.).

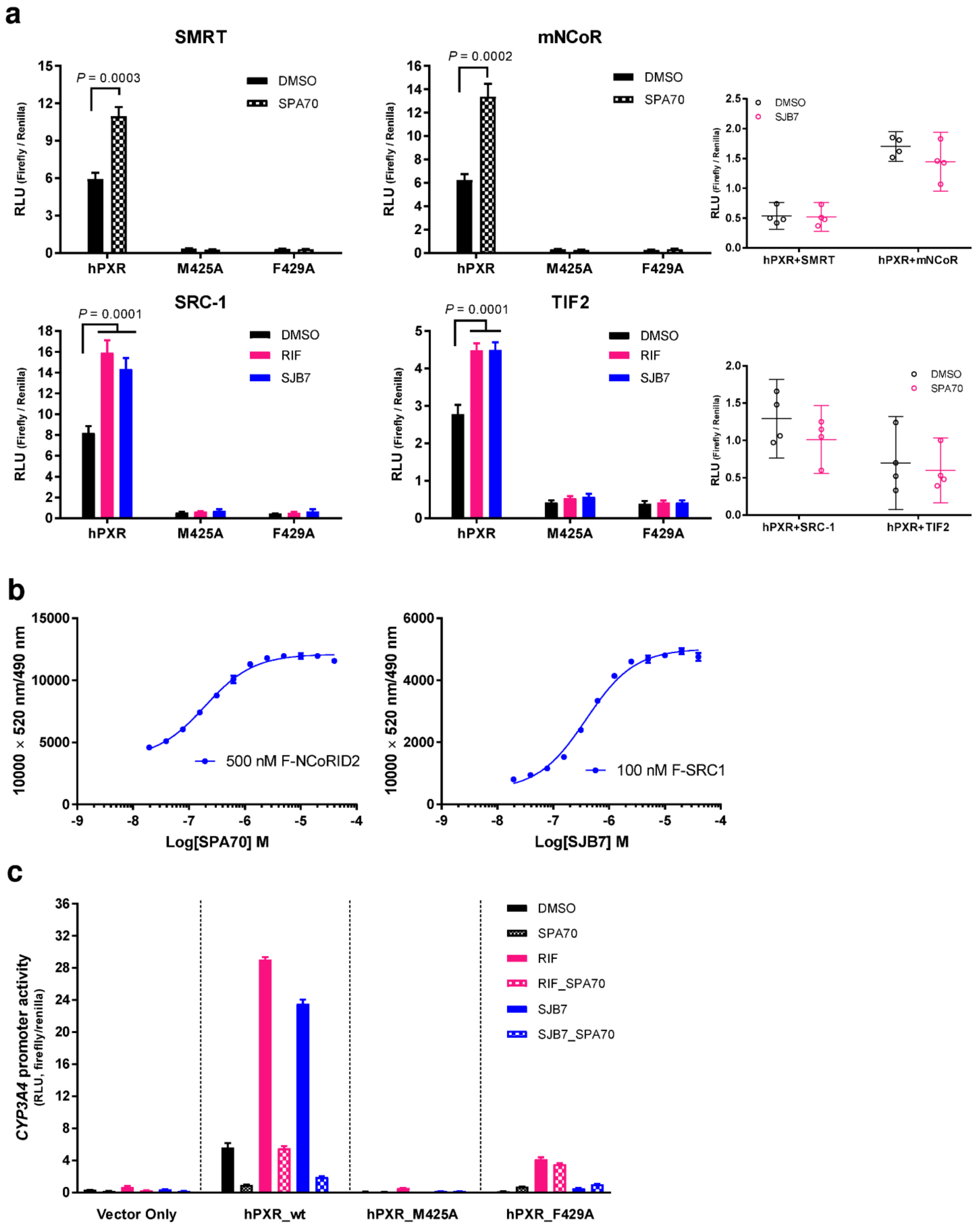

Supplementary Figure 6. SPA70 enhances corepressor association with hPXR, whereas SJB7 enhances coactivator association with hPXR. (**a**) A mammalian two-hybrid assay was used to

measure the association of hPXR with SRC-1, TIF2, SMRT, and mNCoR. VP16-hPXR (hPXR: wild-type hPXR; M425A: hPXR M425A; F429A: hPXR M429A) and GAL4 DBD coregulator (the GAL4 DNA binding-domain fused to the coregulator) were transiently expressed in LS180 cells; the cells were treated with DMSO (0.1%), SPA70 (10 µM), rifampicin (RIF, 4 µM), or SJB7 (4 µM); and the interaction between the two fusion proteins was assayed by quantifying the resultant specific *GAL4-luc* reporter activity (presented as relative luciferase units [RLU]) by normalizing the firefly luciferase activity to that of the *Renilla* luciferase (the internal control). For the effect of SPA70 on hPXR association with SMRT or mNCoR, or the effect of RIF/SJB7 on hPXR association with SRC-1 or TIF2, the data represent the mean ± SEM from six independent assays, and statistical significance was calculated by a *t*-test (for SMRT and mNCoR) or by one-way ANOVA with Tukey's multiple-comparison test (for SRC-1 and TIF2). For the effect of SJB7 on hPXR association with SMRT or mNCoR, or the effect of SPA70 on hPXR association with SRC-1 or TIF2, the data are expressed as the mean and 95% CI from four independent assays. (**b**) A TR-FRET cofactor recruitment assay was used to measure the interaction between hPXR and fluorescently labeled SRC-1 (F-SRC-1) or NCoR peptide (F-NCoR) in response to different concentrations of SPA70 (for F-NCoR) or SJB7 (for F-SRC-1). TR-FRET signals were expressed as 10,000 × the 520 nm/490 nm emission ratio, as described in the online methods. (**c**) SPA70 blocks the agonistic effect of SJB7. Results are shown for vector (pcDNA3), wild-type hPXR (hPXR_wt), hPXR M425A, and hPXR F429A in LS180 cells in response to treatment with 0.1% DMSO (control), RIF (4 µM), SJB7 alone (4 µM), or SJB7 combined with SPA70 (10 µM). CYP3A4 promoter activity was expressed in RLU by normalizing the firefly luciferase activity to that of the *Renilla* luciferase from pRL-TK (as a transfection control). Data are expressed as the mean ± SEM from six independent assays.

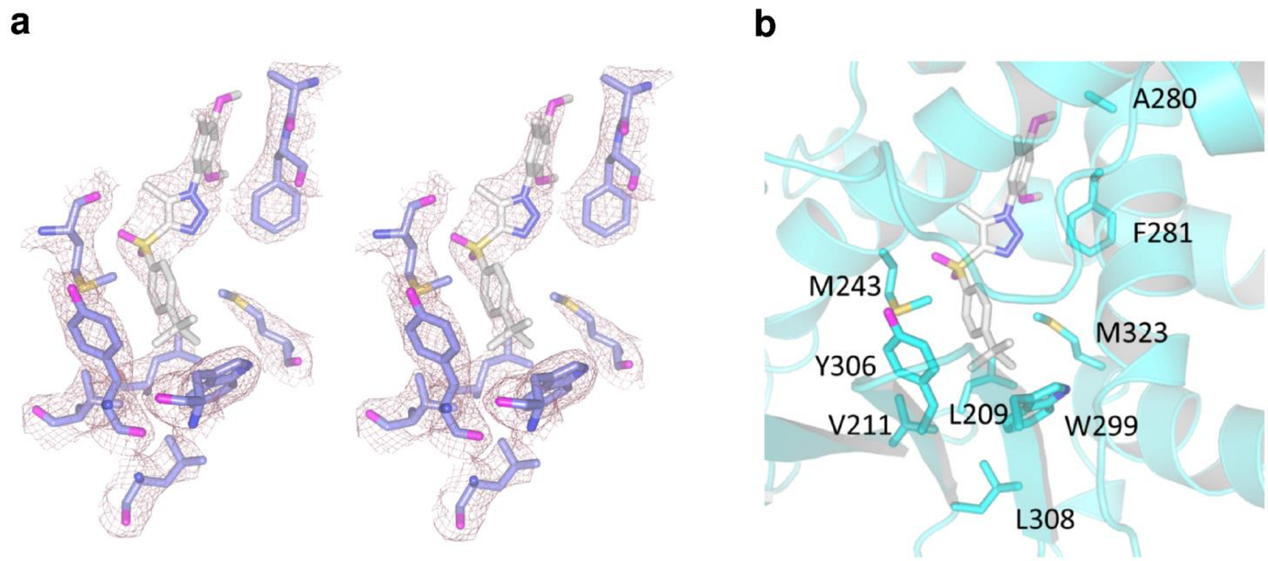

Supplementary Figure 7. Crystal structure of hPXR LBD in complex with SJB7, displaying additional interacting residues. a) Cross-eye stereo view of a section of the 2Fo-Fc map (1.0 sigma) showing the electron density around SJB7 and some of the interacting residues (represented as sticks). Carbon atoms corresponding to SJB7 are shown in white and those of the hPXR LBD in light blue. **b)** For clarity, the selected residues are labeled and illustrated along a cartoon representation of the hPXR LBD (carbon atoms are shown in cyan). Color code for the stick representations: blue, nitrogen; magenta, oxygen; yellow, sulfur.

Sequence	Charge	Start	End	Structure	± SJB7	± SRC-1 + SJB7	± SPA70	± NCOR1-3 + SPA70
TEEQRM	2	144	149	H1	-4 (4)	4 (3)	-5 (3)	2 (3)
MIRELMD	2	150	156	H1	-9 (4)	0 (2)	-6 (4)	-4 (4)
MIRELMDAQM	2	150	159	H1	-14 (6)	-1 (1)	-9 (3)	-2 (3)
LMDAQMKT	1	154	161	H1	-13 (4)	2 (4)	-18 (5)	1 (5)
LMDAQMKT	2	154	161	H1	-11 (3)	0 (2)	-13 (4)	0 (5)
KTFDTTF	2	160	166	H1	1 (4)	2 (3)	-1 (5)	2 (4)
FRLPGVL	2	172	178	random coil	-1 (6)	4 (2)	3 (6)	1 (4)
SSGCELPESLQAPSRE	2	179	194	random coil/H2	-1 (5)	1 (3)	1 (6)	-1 (5)
EAAKWSQVRKDLCSL	2	195	209	H2/random coil	N/A	1 (2)	1 (2)	3 (5)
KVSLQL	2	210	215	sheet	-3 (4)	-9 (3)	-4 (4)	-5 (4)
QLRGEDGSVWNYKPPADS...	4	214	237	sheet	1 (3)	3 (3)	4 (3)	1 (5)
NYKPPADSGGKEIF	2	224	237	sheet	0 (6)	1 (3)	-2 (5)	1 (4)
NYKPPADSGGKEIF	3	224	237	sheet	-2 (5)	2 (3)	-1 (9)	-3 (3)
SLLPHMAD	2	238	245	H3	2 (4)	-7 (4)	0 (5)	-6 (3)
SLLPHMADM	2	238	246	H3	N/A	-9 (3)	-3 (5)	-9 (4)
SLLPHMADMSTYM	2	238	250	H3	-7 (5)	-16 (3)	-14(3)	-12(3)
MSTYM	1	246	250	H3	-19 (5)	-20 (2)	-26 (6)	-13 (4)
MFKGIISF	2	250	257	H3	-13 (4)	-1 (5)	-16 (4)	1 (2)
FKGIISF	1	251	257	H3	-13 (4)	-2 (1)	-12 (4)	-2 (2)
FKGIISF	2	251	257	H3	-15 (3)	-3 (1)	-14 (2)	-2 (2)
AKVISY	1	258	263	H3	0 (4)	3 (2)	-1 (4)	0 (3)
AKVISY	2	258	263	H3	0 (3)	3 (2)	-1 (4)	0 (2)
FRDLPIED	2	264	271	H3/H4	0 (3)	0 (2)	-3 (4)	-2 (4)
FRDLPIEDQ	1	264	272	H3/H4	2 (5)	-1 (2)	0 (5)	1 (4)
FRDLPIEDQ	2	264	272	H3/H4	1 (3)	-1 (2)	-1 (3)	0 (3)
FRDLPIEDQISL	2	264	275	H3/H4	1 (4)	1 (4)	-2 (3)	-2 (3)
FRDLPIEDQISLL	2	264	276	H3/H4	0 (3)	-1 (1)	-1 (2)	-2 (2)
ISLLKGAA	2	273	280	H4	-3 (1)	-3 (0)	-4 (1)	-3 (1)
ISLLKGAAF	2	273	281	H4/H5	-3 (2)	-2 (1)	-4 (2)	-4 (1)
ISLLKGAAFE	2	273	282	H4/H5	-3 (3)	-2 (2)	-4 (2)	-2 (1)
LKGAAF	2	276	281	H4/H5	-4 (4)	-3 (2)	-6 (3)	-5 (2)
FELCQLRF	2	281	288	H5	-3 (2)	0 (1)	-2 (2)	0 (2)
ELCQL	1	282	286	H5	-5 (4)	1 (1)	1 (4)	N/A
ELCQLRF	2	282	288	H5	-1 (1)	0 (0)	0 (1)	0 (1)
LCQLRF	2	283	288	H5	2 (4)	1 (3)	-2 (2)	4 (2)
NAETGTWECGRL	2	293	304	sheet	-10 (4)	-7 (3)	-12 (6)	N/A
NAETGTWECGRLSY	2	293	306	sheet	-15 (5)	-9 (4)	-17 (6)	-11 (4)
NAETGTWECGRLSYC	2	293	307	sheet	-10 (5)	-8 (4)	-13 (4)	-8 (2)
CLEDTAGGFQQL	1	307	318	sheet/H6	3 (3)	1 (3)	5 (5)	0 (5)
CLEDTAGGFQQL	2	307	318	sheet/H6	3 (4)	1 (3)	2 (6)	1 (4)
LEDTAGGFQQL	1	308	318	sheet/H6	1 (4)	1 (3)	3 (6)	-1 (4)
LLEPML	1	319	324	H6/H7	1 (6)	0 (3)	-3 (5)	-2 (4)
LLEPMLKF	2	319	326	H6/H7	-2 (4)	3 (3)	-9 (5)	-2 (4)
HYMLKKLQL	3	327	335	H7	1 (2)	2 (2)	-6 (4)	N/A
HEEEY	1	336	340	H8	-1 (2)	-1 (1)	N/A	-1 (1)
YVLMQA	1	340	345	H8	2 (4)	0 (1)	-1 (3)	0 (1)
MQAISL	1	343	348	H8	0 (2)	-1 (4)	0 (1)	0 (1)
QAISL	1	344	348	H8	-1(1)	0 (1)	-1 (1)	-3 (1)
ISLFSPDRPGVLQHRVVDQL	3	346	365	H8/H9	5 (4)	1 (3)	0 (4)	-2 (3)
FSPDRPGVLQHRVVDQL	3	349	365	H8/H9	3 (2)	3 (3)	N/A	-1 (3)
FSPDRPGVLQHRVVDQLQEQ	3	349	368	H8/H9	-4 (5)	2 (3)	-5 (4)	0 (3)
AITLKS	1	370	375	H9	-2 (1)	0 (0)	-1 (1)	-1 (1)
AITLKS	2	370	375	H9	-1 (2)	0 (0)	-1 (1)	0 (1)
YIECNRPQPAHRFLFL	3	376	391	H9/H10	1 (4)	4 (3)	2 (4)	1 (5)
LKIMAM	1	391	396	H9/H10	0 (1)	1 (1)	0 (1)	0 (1)
LKIMAM	2	391	396	H10	-3 (2)	0 (0)	-1 (3)	-2 (3)
KIMAML	1	392	397	H10	0 (1)	1 (1)	0 (1)	0 (1)
KIMAML	2	392	397	H10	-3 (2)	0 (0)	-1 (3)	-2 (3)
LTELRSINAQHTQRL	2	397	411	H10	-2 (4)	-4 (3)	-1 (6)	-4 (2)
TELRSINAQHTQRL	3	398	411	H10	-1 (4)	-3 (3)	-3 (5)	-3 (3)
RSINAQHTQRL	3	401	411	H10	N/A	-7 (3)	N/A	N/A
LRIQDIHPFATPLM	2	412	425	H10/H12	1 (5)	-1 (3)	1 (6)	-3 (5)
LRIQDIHPFATPLM	3	412	425	H10/H12	2 (5)	-1 (3)	2 (6)	-3 (4)
LRIQDIHPFATPLMQEL	2	412	428	H10/H12	3 (6)	-1 (4)	2 (4)	-6 (3)
LRIQDIHPFATPLMQEL	3	412	428	H10/H12	1 (5)	-1 (4)	2 (5)	-6 (4)
FGITGS	1	429	434	H12	0 (4)	4 (2)	4 (5)	-2 (3)

-50 -40 -30 -20 -10 0 10 20 30 40 50

Supplementary Figure 8. Differential HDX data for SJB7 and SPA70. The sequence of the peptide is provided, along with the charge state of the ion (z) and the hPXR starting and ending residue numbers. The values listed under each HDX experiment demonstrate the averaged difference in the percentage of deuterium incorporation for the corresponding peptide in two different states across all exchange time points (i.e., 10 s, 60 s, 300 s, and 900 s), and the numbers in parentheses in the columns for each HDX experiment represent the standard deviation calculated from three replicates. The HDX data from all overlapping peptides were consolidated to individual amino acid values by using a residue averaging approach[1]. We applied a smooth-color gradient key (shown at the bottom of the figure) to represent the related protein regions with statistically significant differential deuterium incorporations (i.e., regions that show protection against solvent exchange). HDX Workbench (the software used in this study) automates HDX color according to this key. The statistical summary was derived from a two-way ANOVA for each pairwise experiment ($P < 0.05$). A negative value represents decreased deuterium incorporation or stabilization in the corresponding region of the receptor protein when a binding event occurs. Peptides exhibiting statistically insignificant or undetectable changes are colored gray. N/A indicates that the peptide was not detected in that experiment.

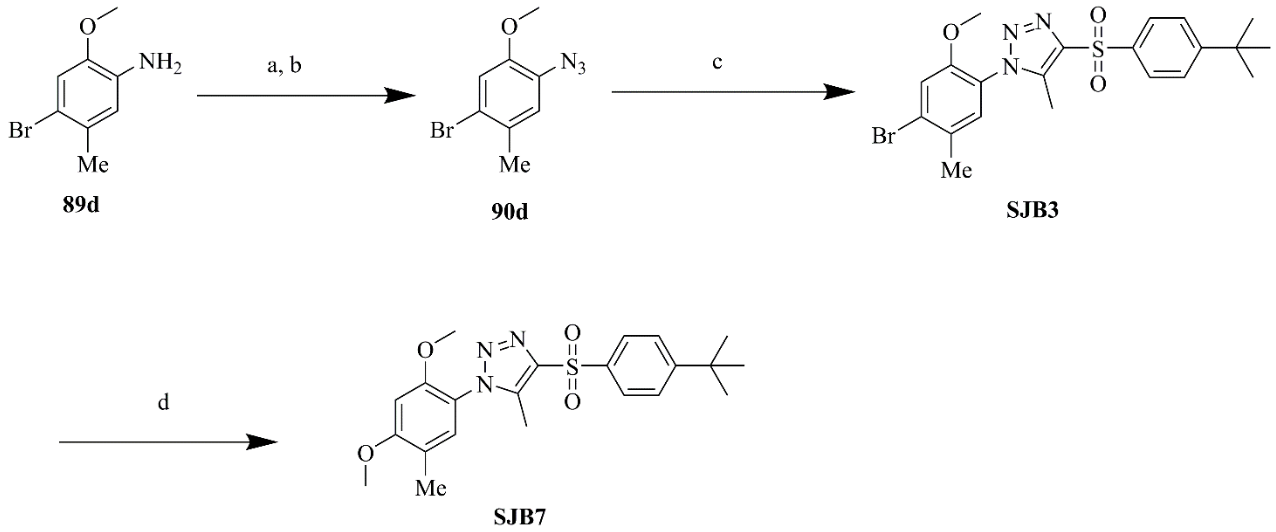

Supplementary Figure 9. Synthesis of SJB7 (LC-24). Reagents and conditions: (a) concentrated HCl, $NaNO_2$, H_2O, 0 °C for 15 min; (b) NaN_3, H_2O, room temperature for 2 h; (c) MeONa, 1-((4-(tert-butyl)phenyl)sulfonyl)propan-2-one, MeOH, 60 °C overnight. (d) MeONa, $CuCl_2$, DMF, 130 °C overnight.

a ^{1}H NMR spectrum of compound **SJA1** (**LC-8**)

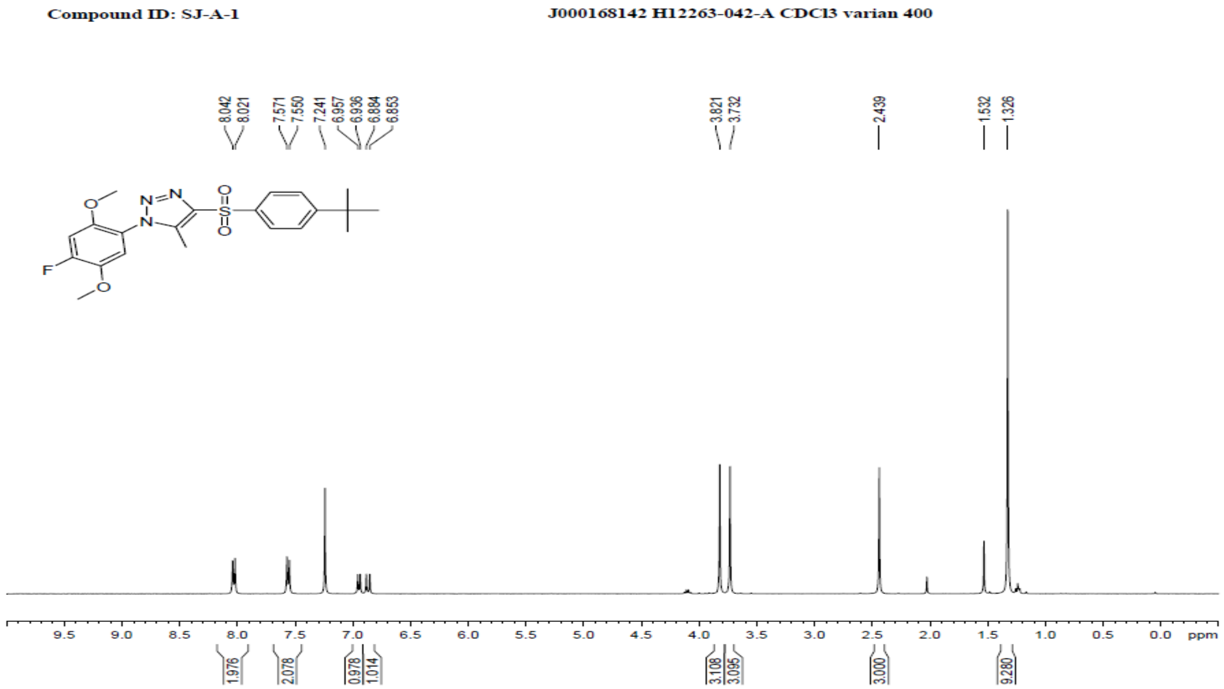

^{13}C NMR spectrum of compound **SJA1** (**LC-8**)

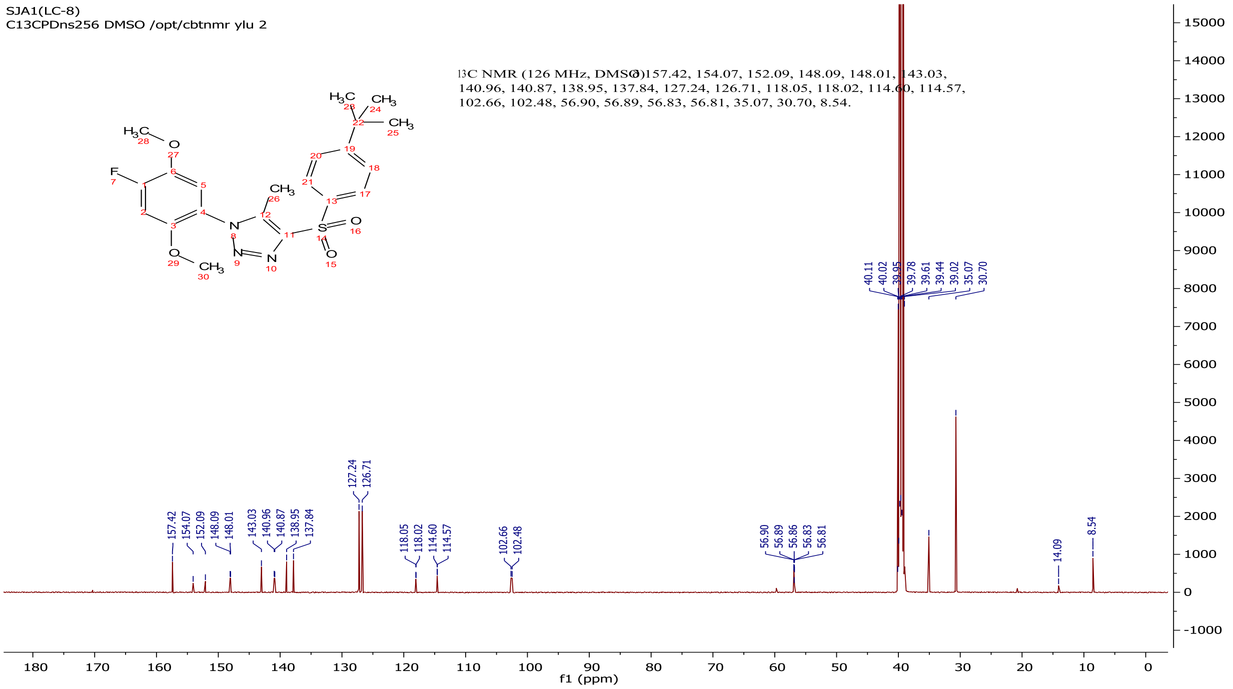

b ^{1}H NMR spectrum of compound **SJA3** (**LC-10**)

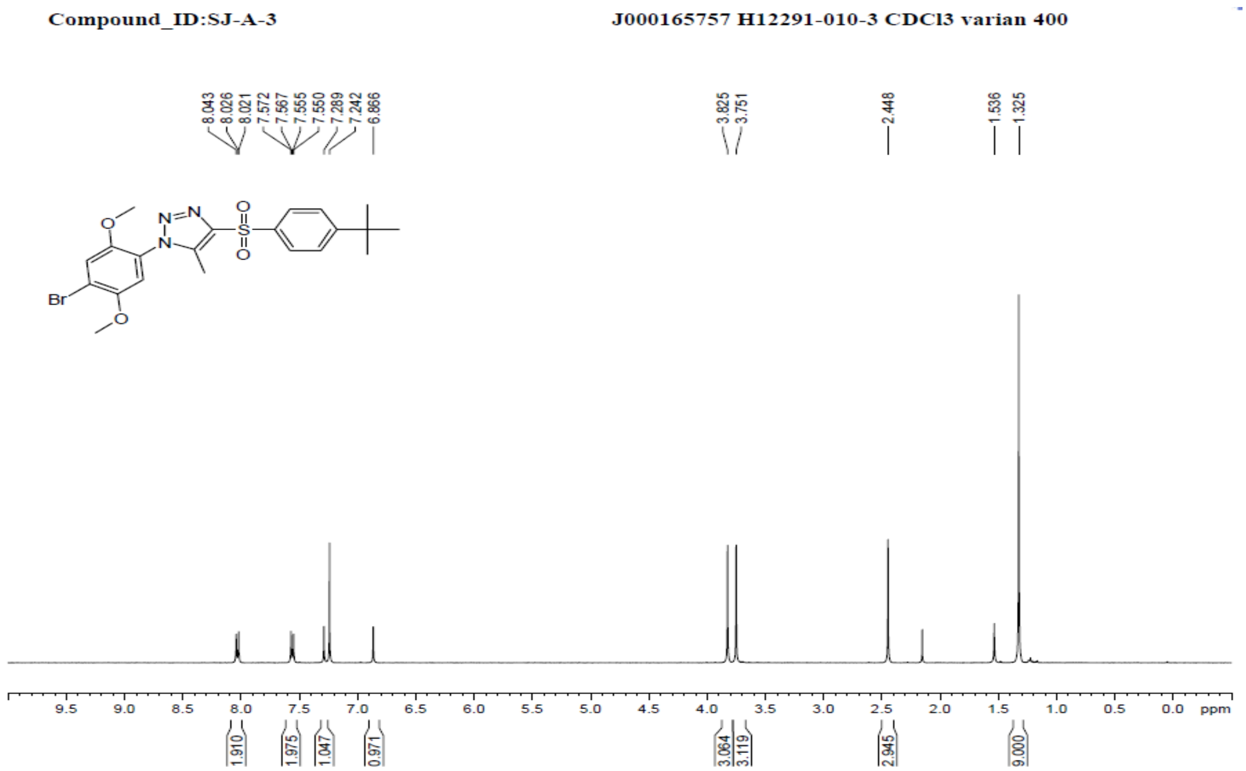

^{13}C NMR spectrum of compound **SJA3** (**LC-10**)

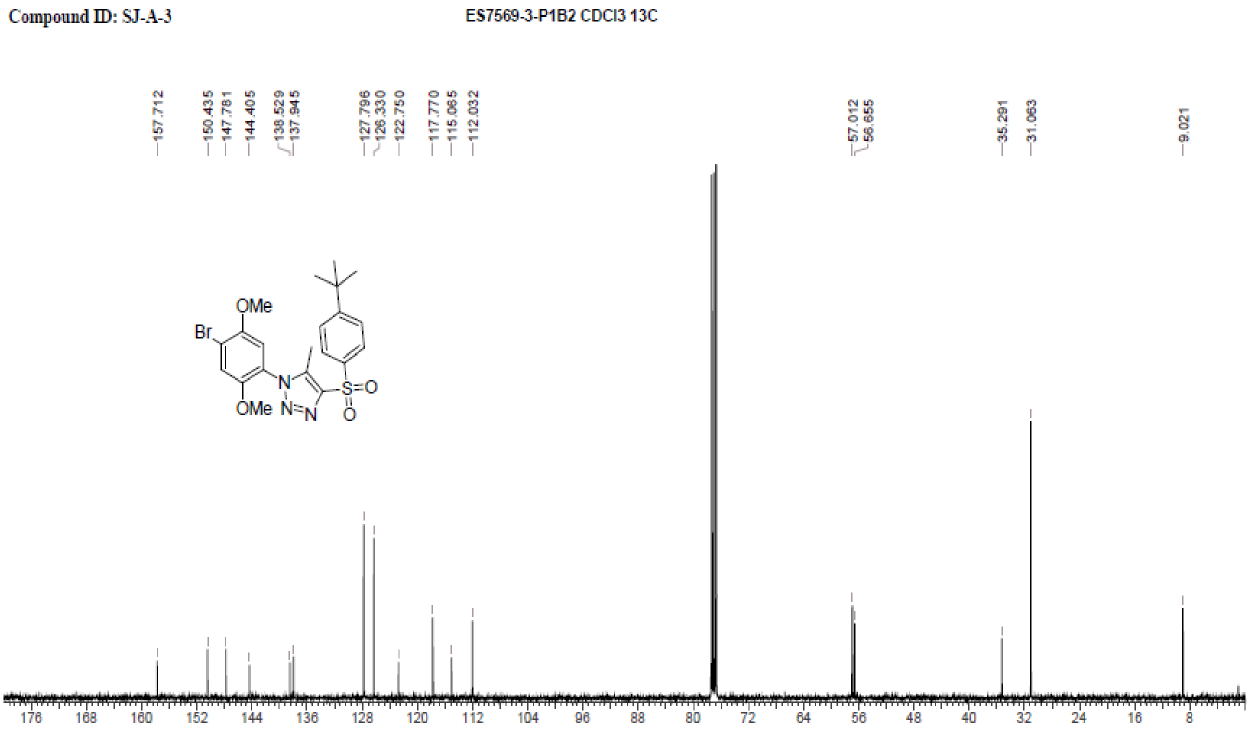

C ^{1}H NMR spectrum of compound **SJA5** (**LC-12**)

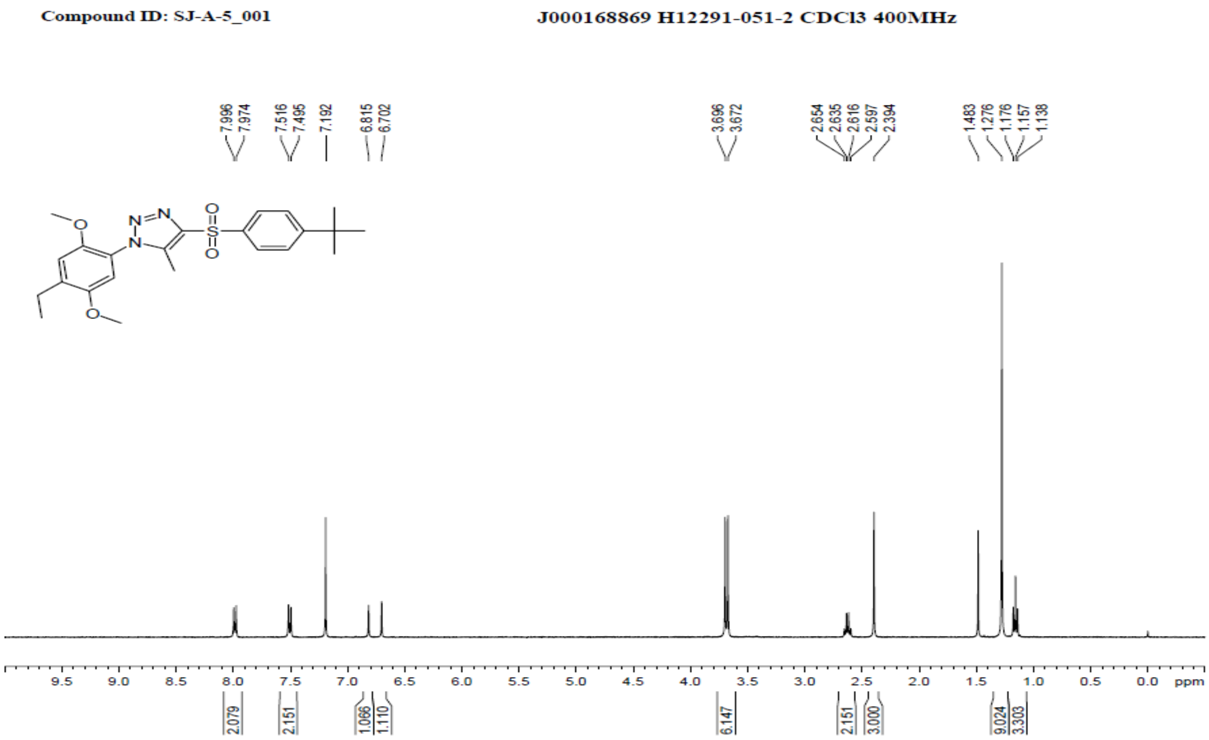

^{13}C NMR spectrum of compound **SJA5** (**LC-12**)

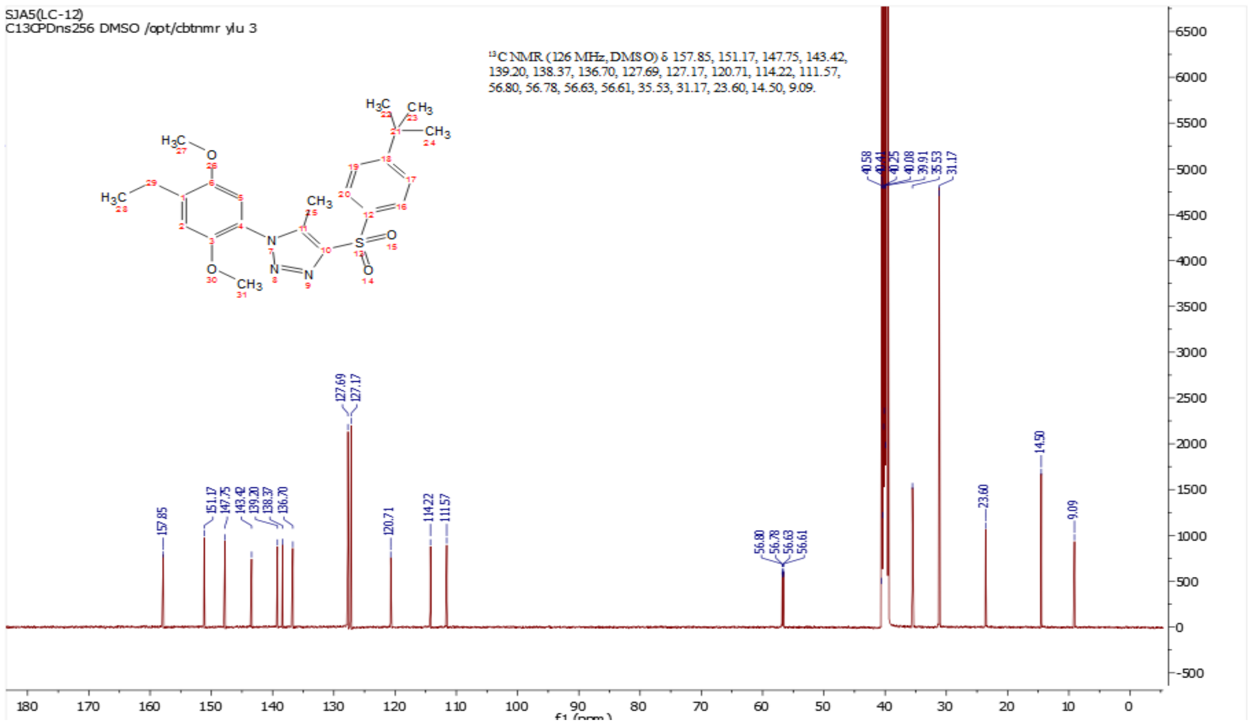

d ^{1}H NMR spectrum of compound **SJA6** (**LC-13**)

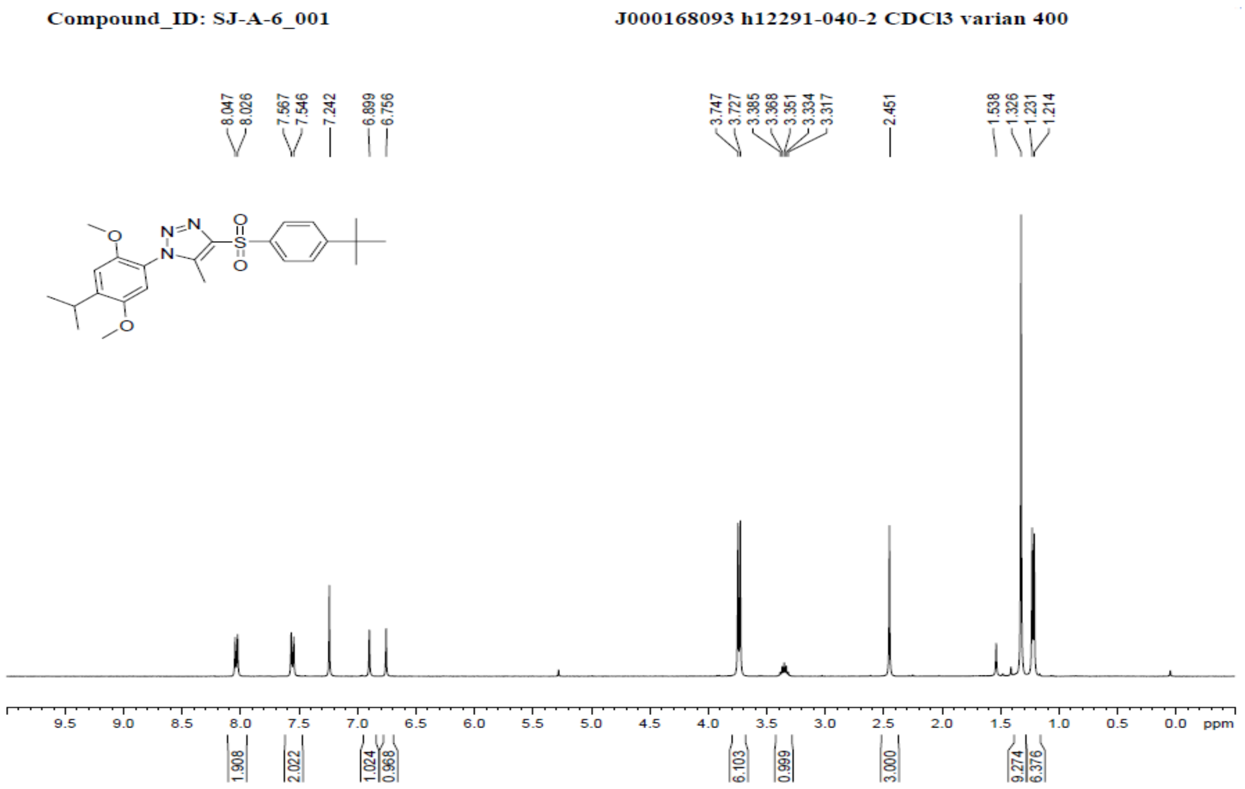

^{13}C NMR spectrum of compound **SJA6** (**LC-13**)

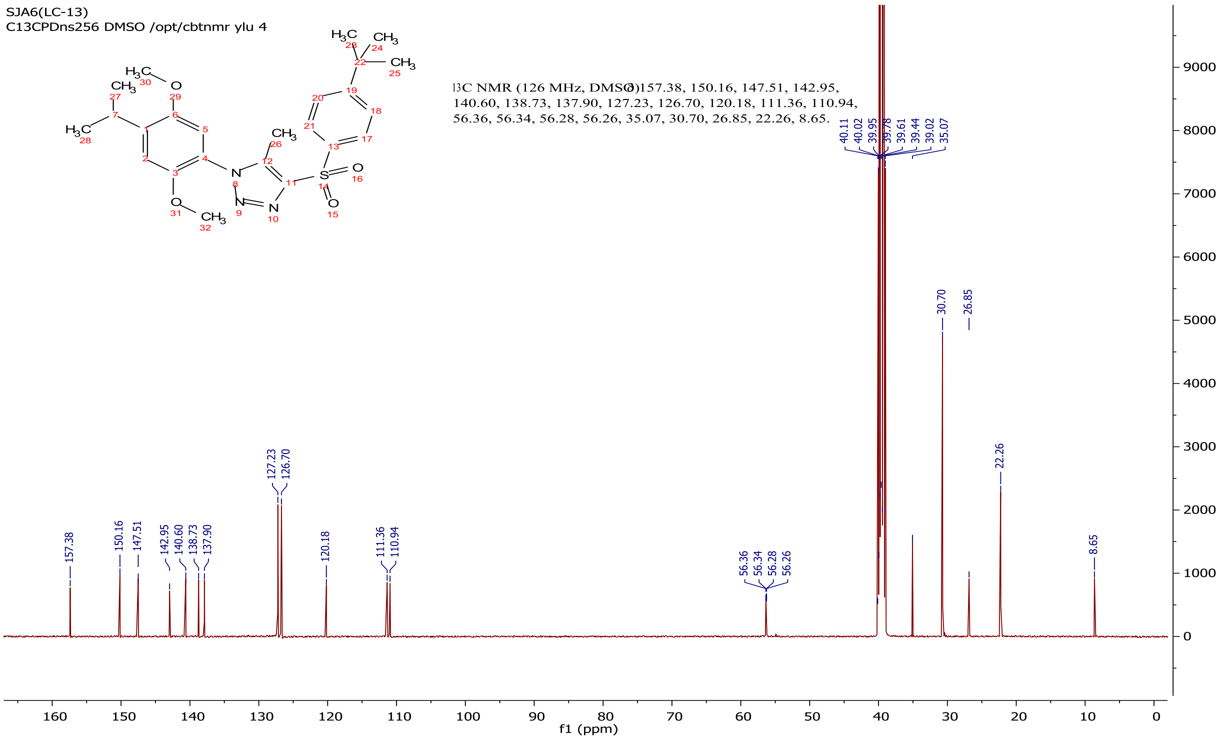

e ^{1}H NMR spectrum of compound **SJA7** (**LC-14**)

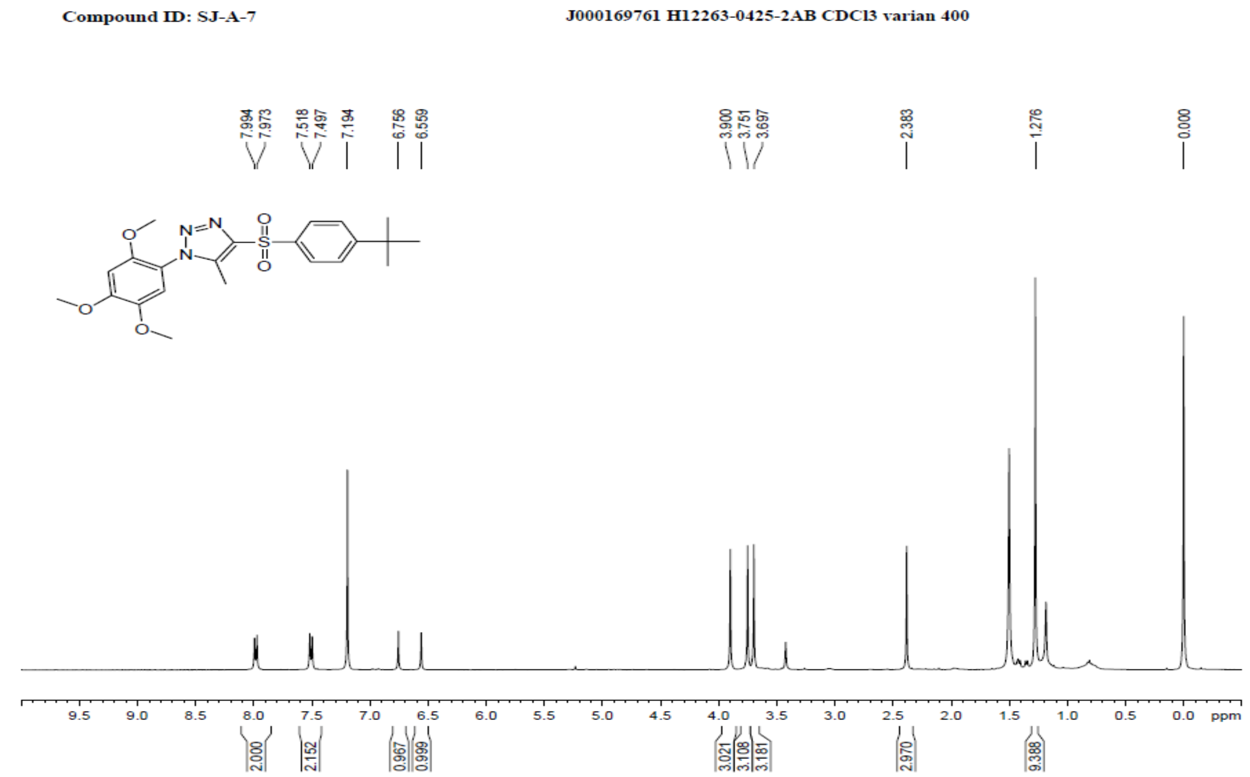

^{13}C NMR spectrum of compound **SJA7** (**LC-14**)

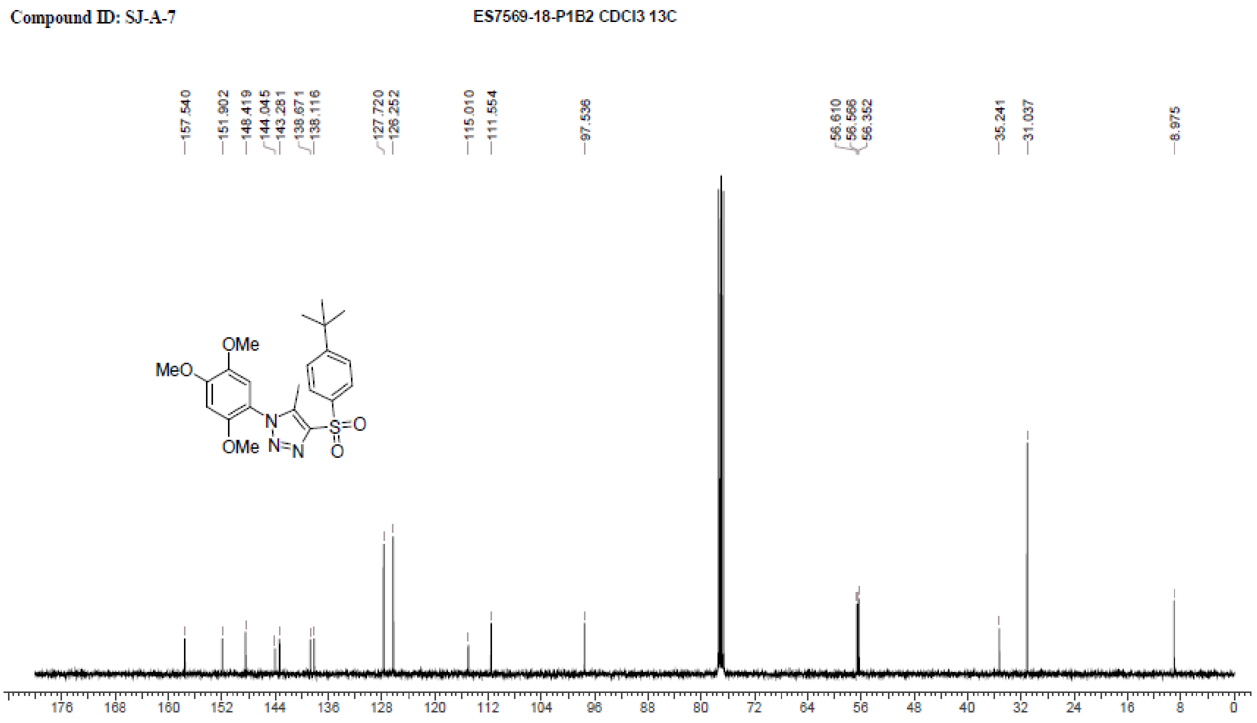

f ^{1}H NMR spectrum of compound **SJA8** (**LC-15**)

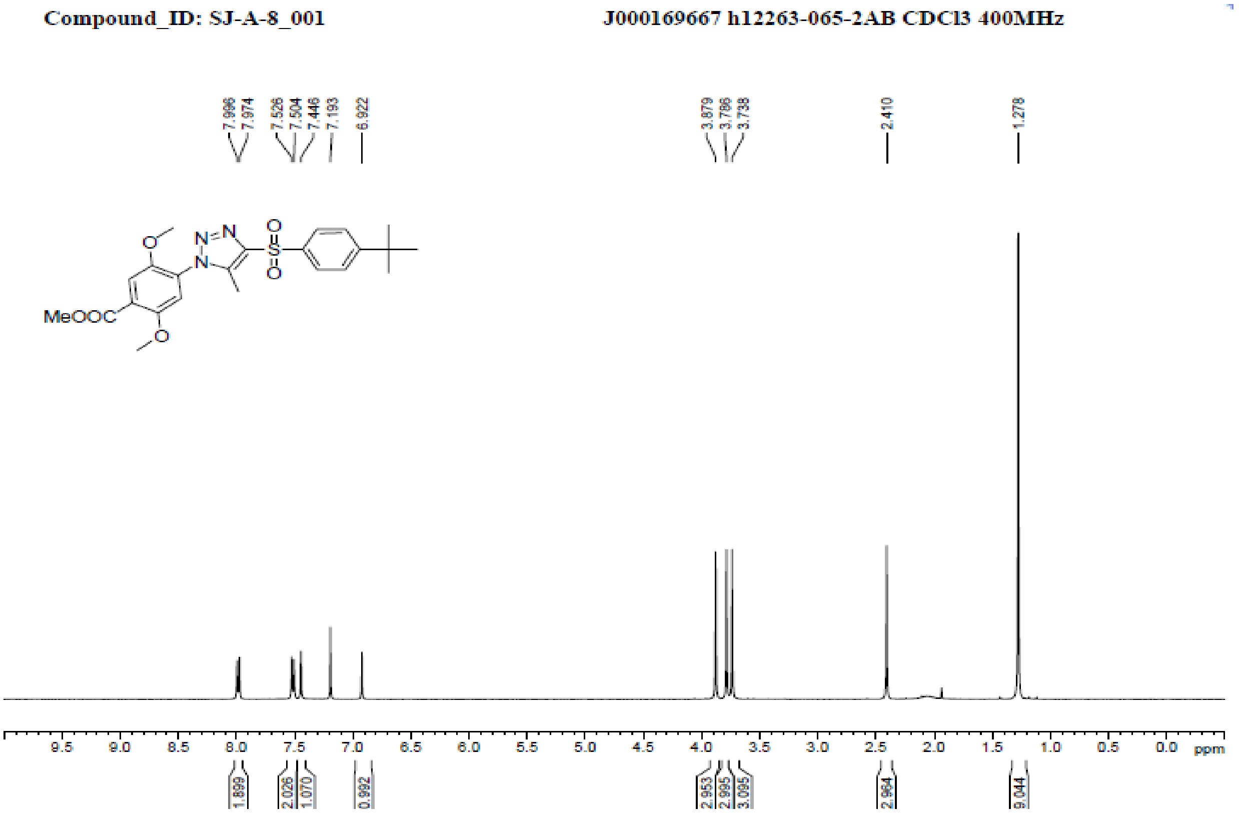

^{13}C NMR spectrum of compound **SJA8** (**LC-15**)

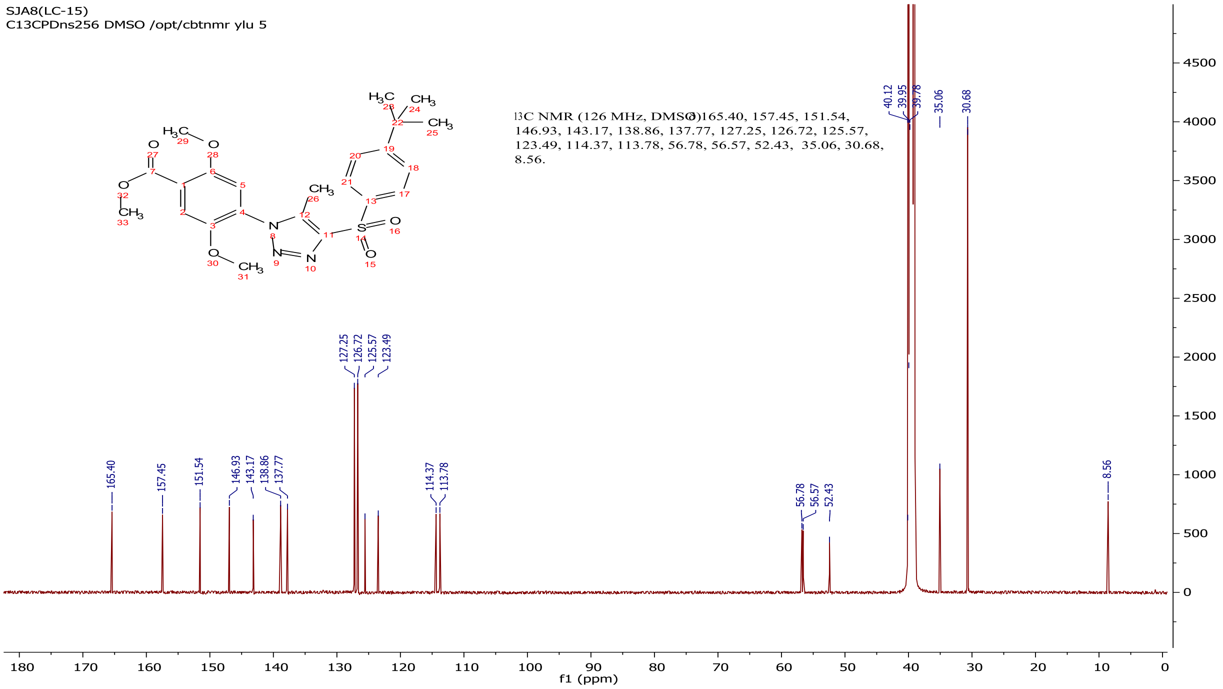

g ^{1}H NMR spectrum of compound **SJA9** (**LC-16**)

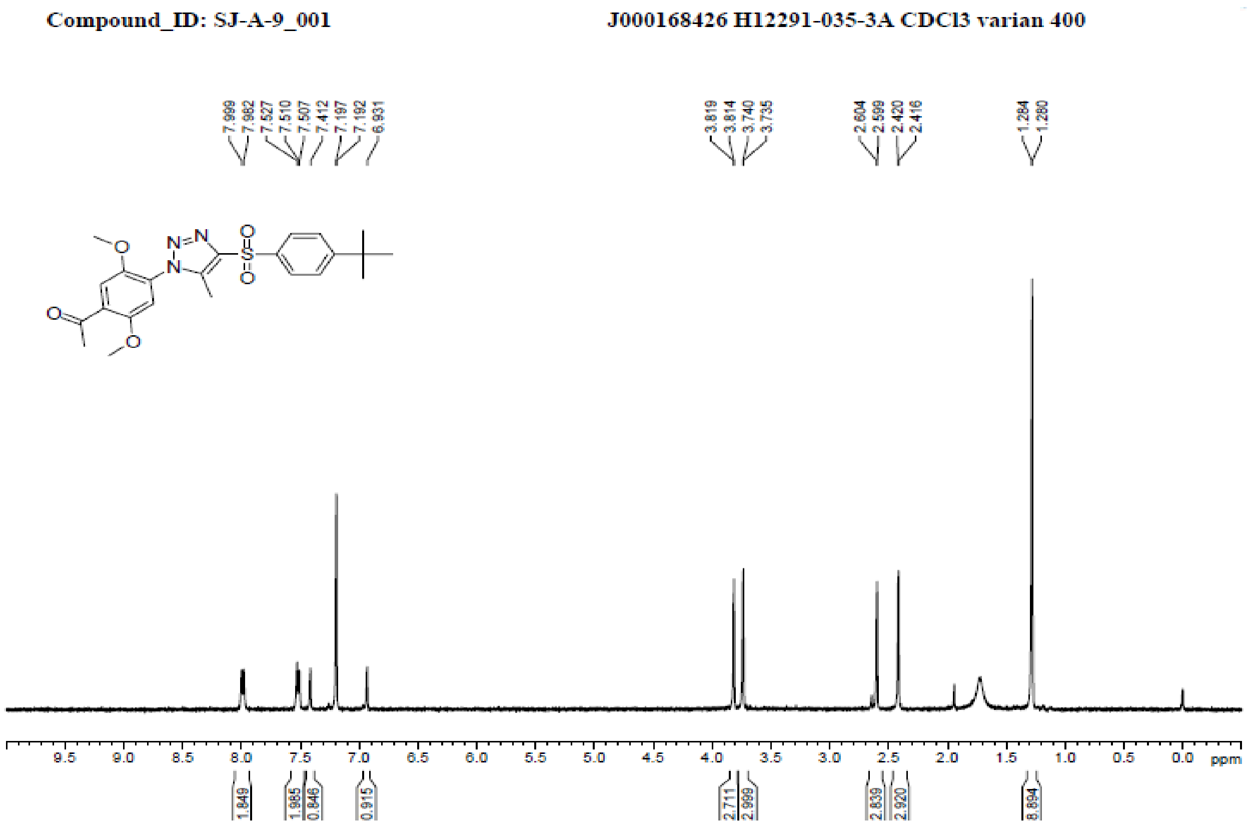

^{13}C NMR spectrum of compound **SJA9** (**LC-16**)

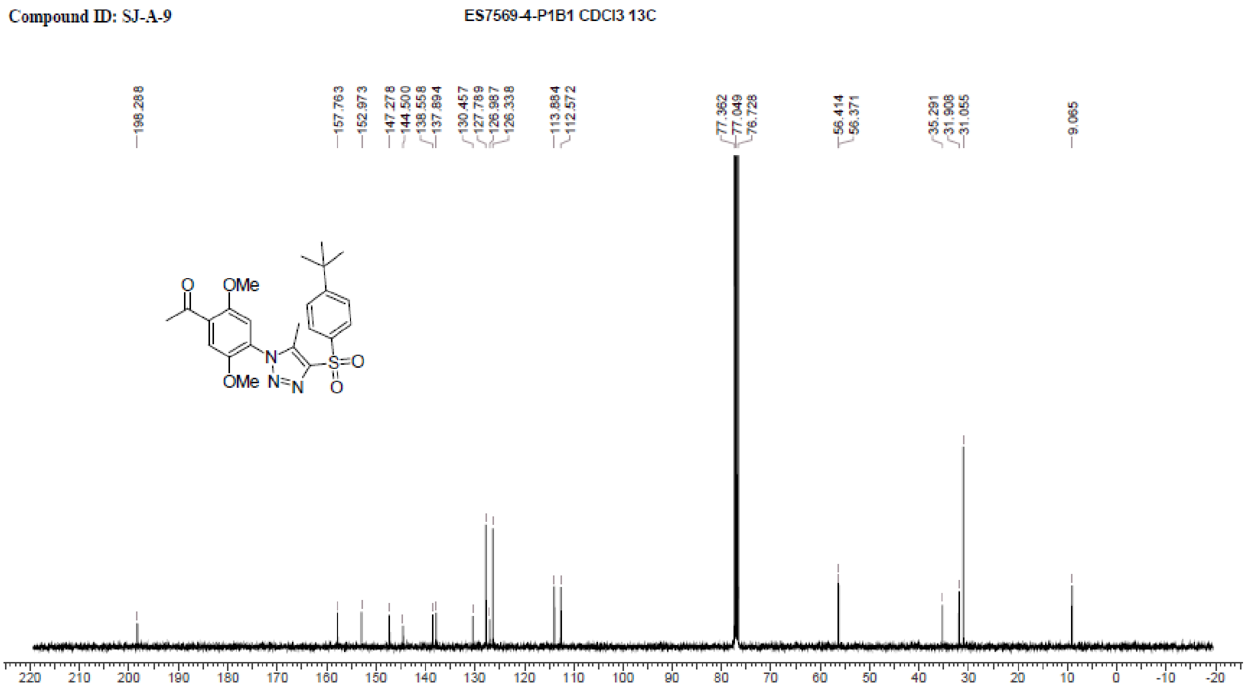

h ^{1}H NMR spectrum of compound **SJA10** (**LC-17**)

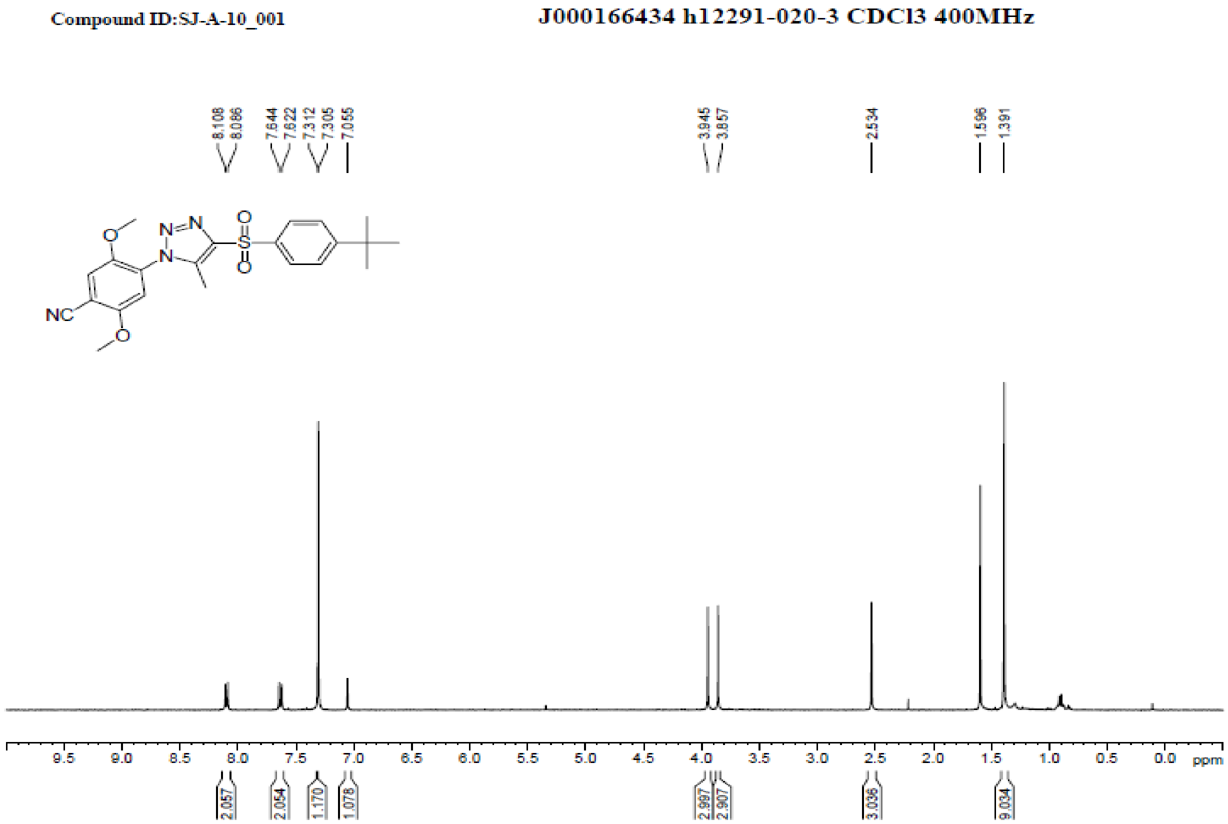

^{13}C NMR spectrum of compound **SJA10** (**LC-17**)

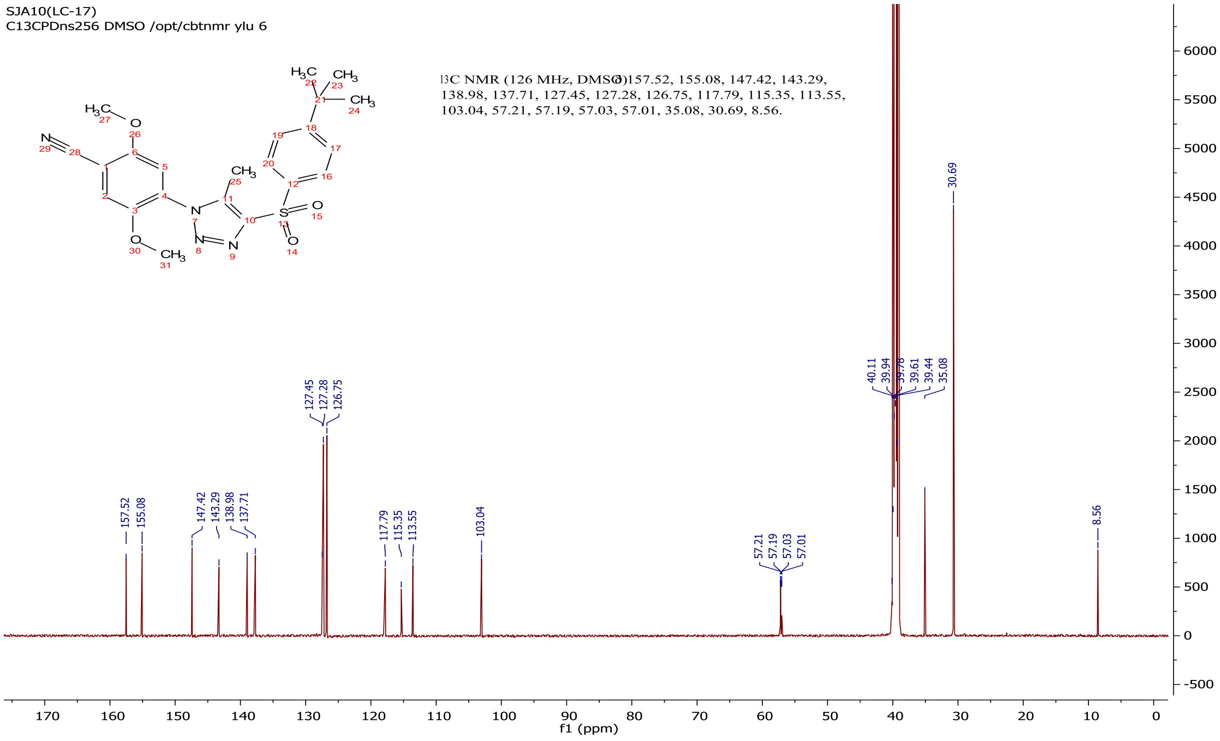

i ^{1}H NMR spectrum of compound **SJB4** (**LC-21**)

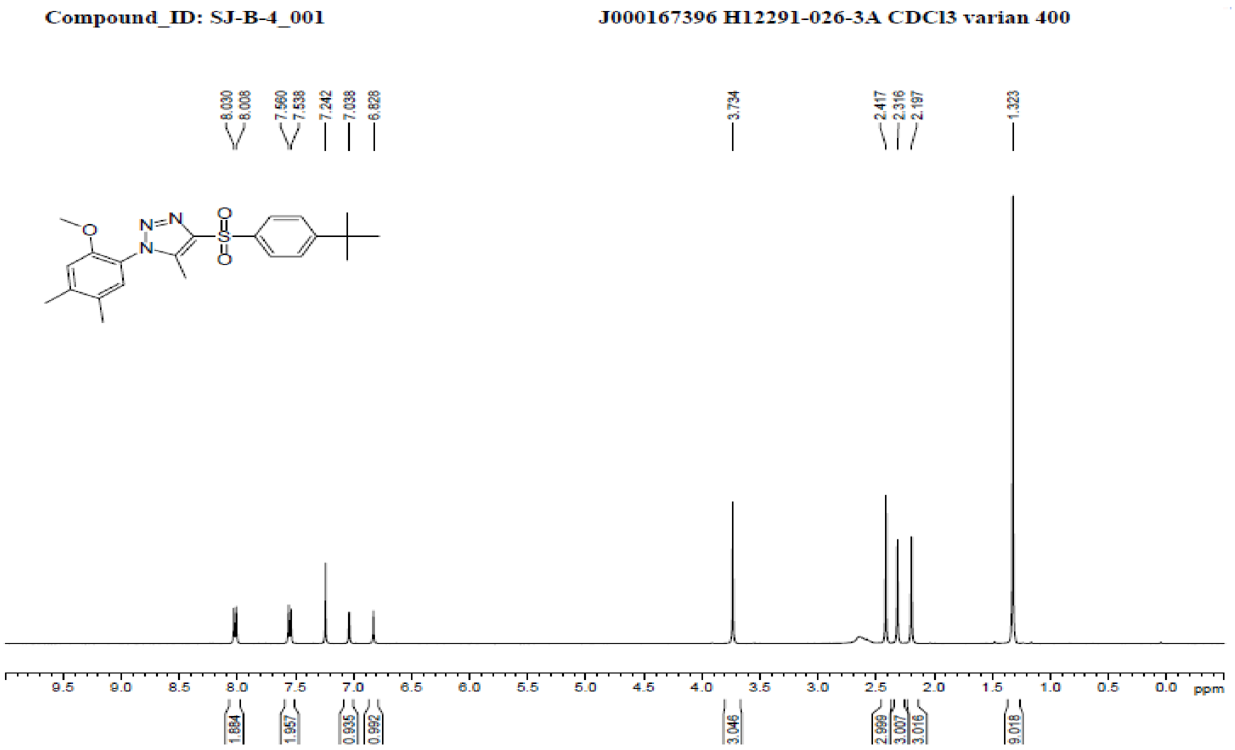

^{13}C NMR spectrum of compound **SJB4** (**LC-21**)

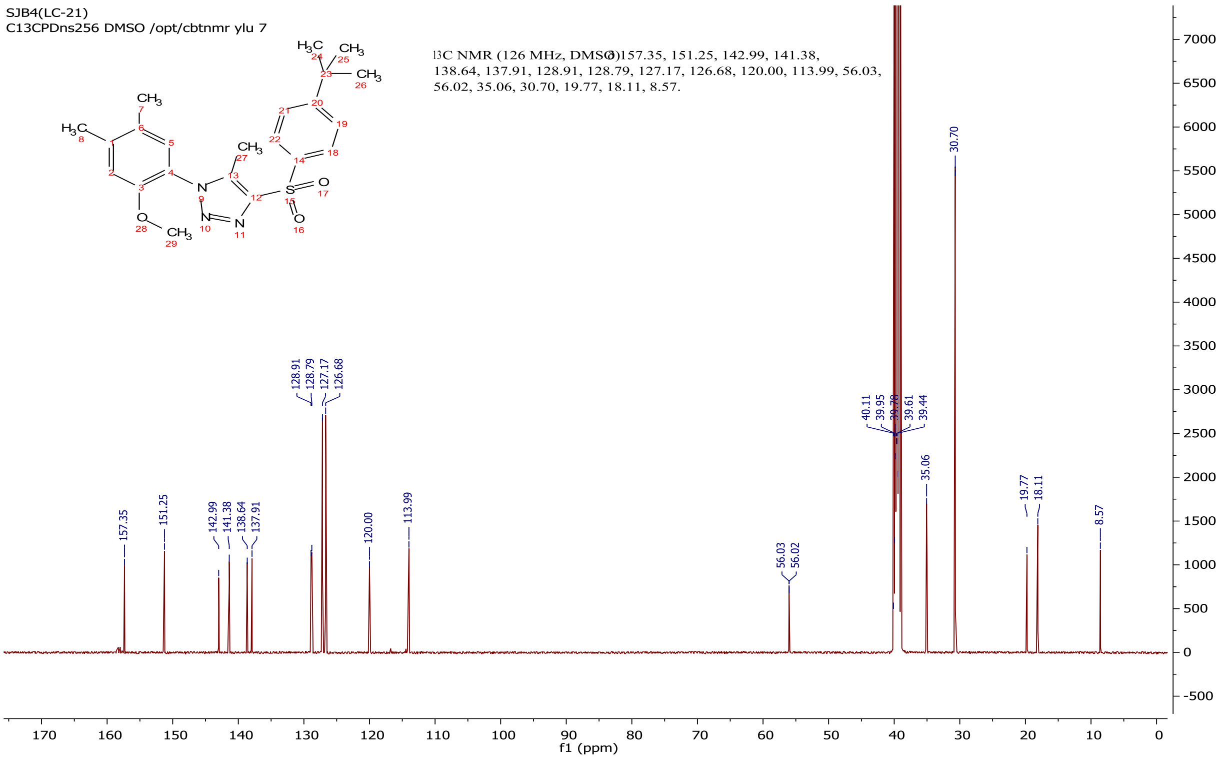

j ^{1}H NMR spectrum of compound **SJB7** (**LC-24**)

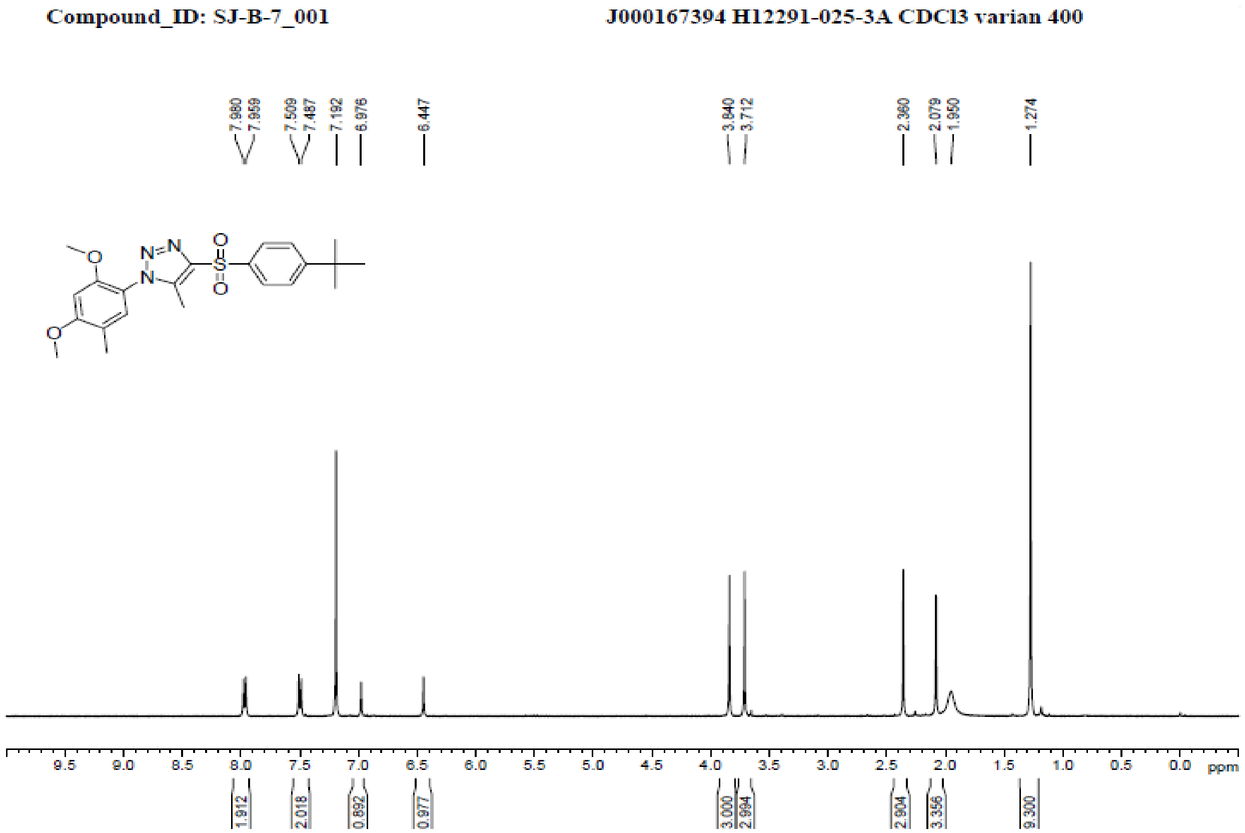

^{13}C NMR spectrum of compound **SJB7** (**LC-24**)

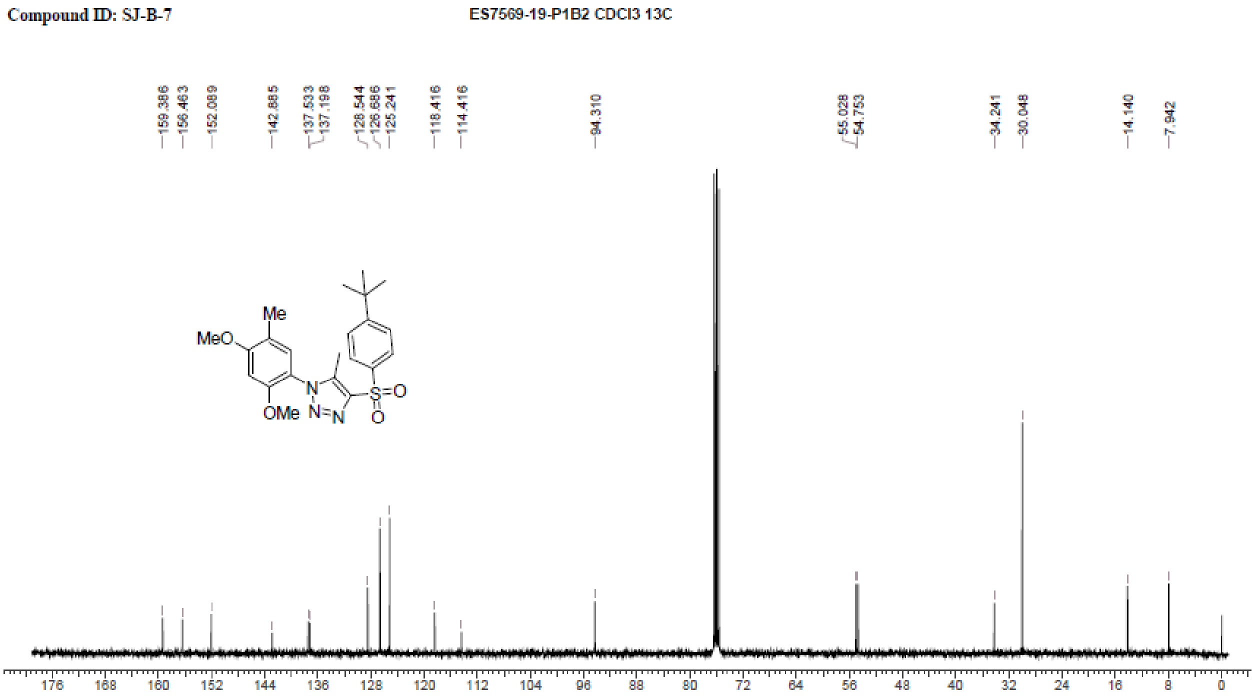

k ^{1}H NMR spectrum of compound **SJB10** (**LC-27**)

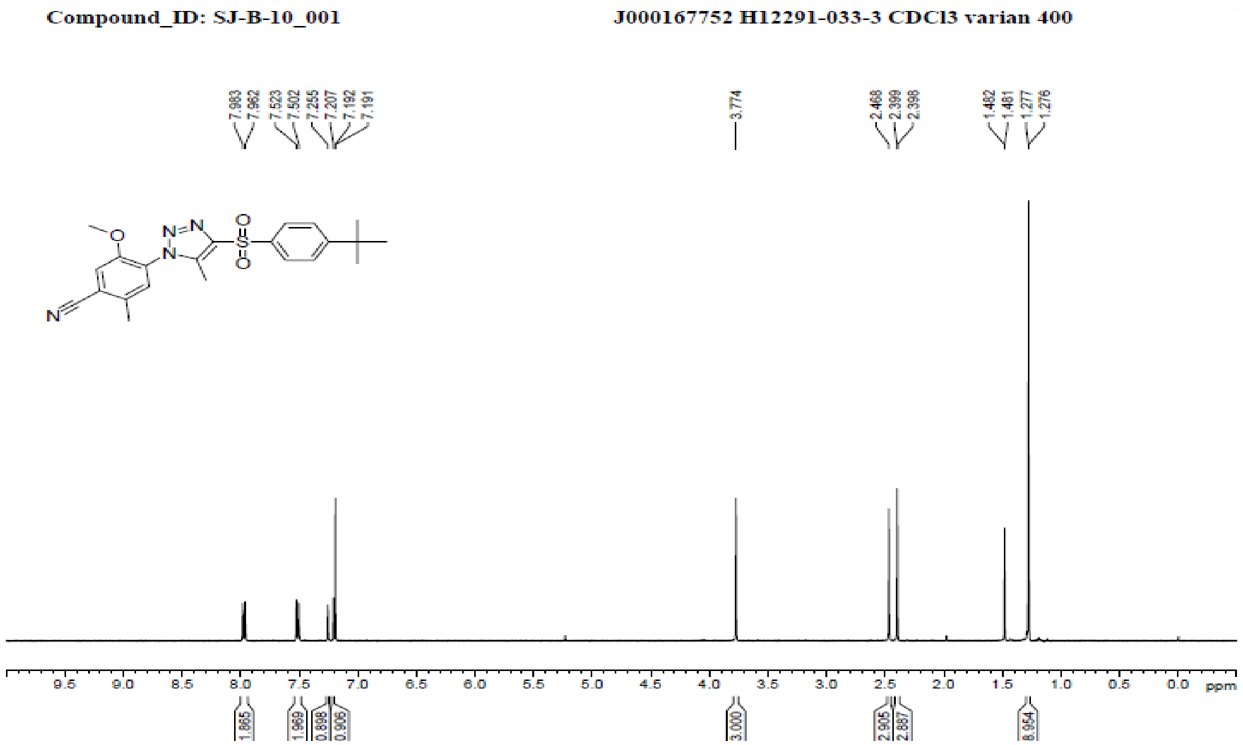

^{13}C NMR spectrum of compound **SJB10** (**LC-27**)

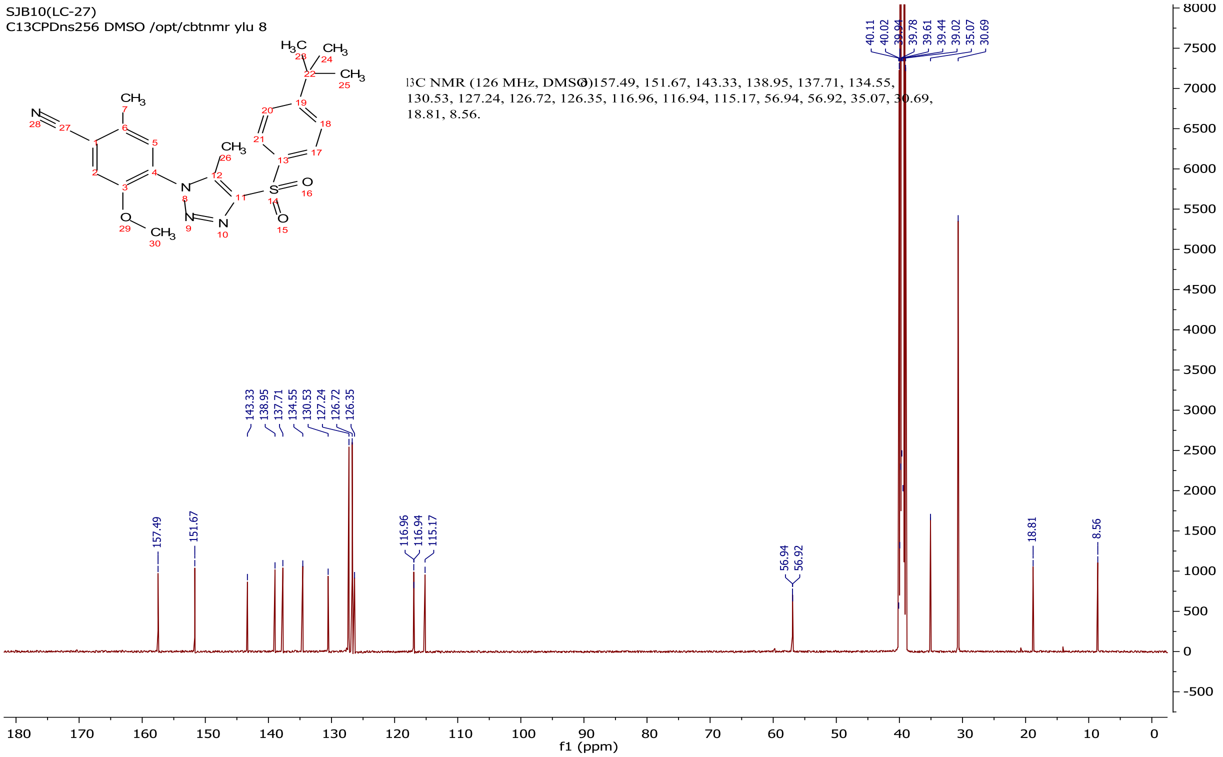

l ^{1}H NMR spectrum of compound **SJC2** (**LC-28**)

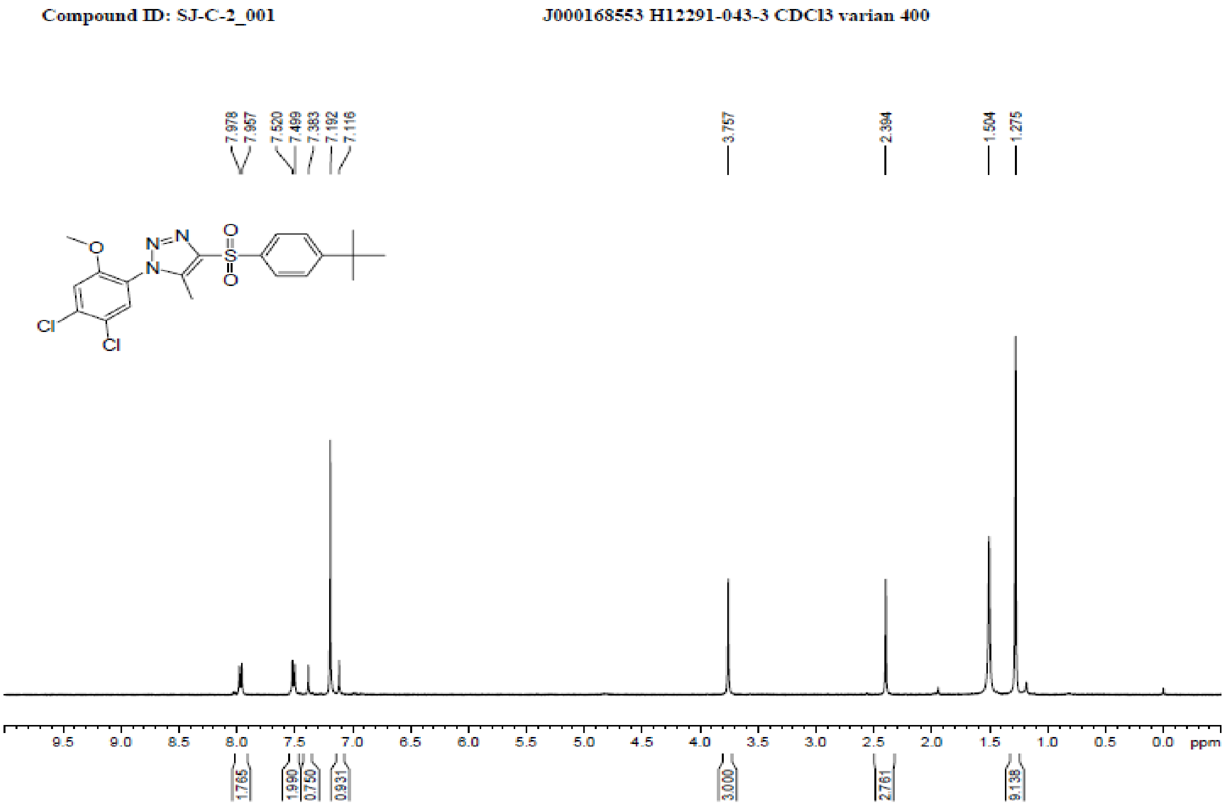

^{13}C NMR spectrum of compound **SJC2** (**LC-28**)

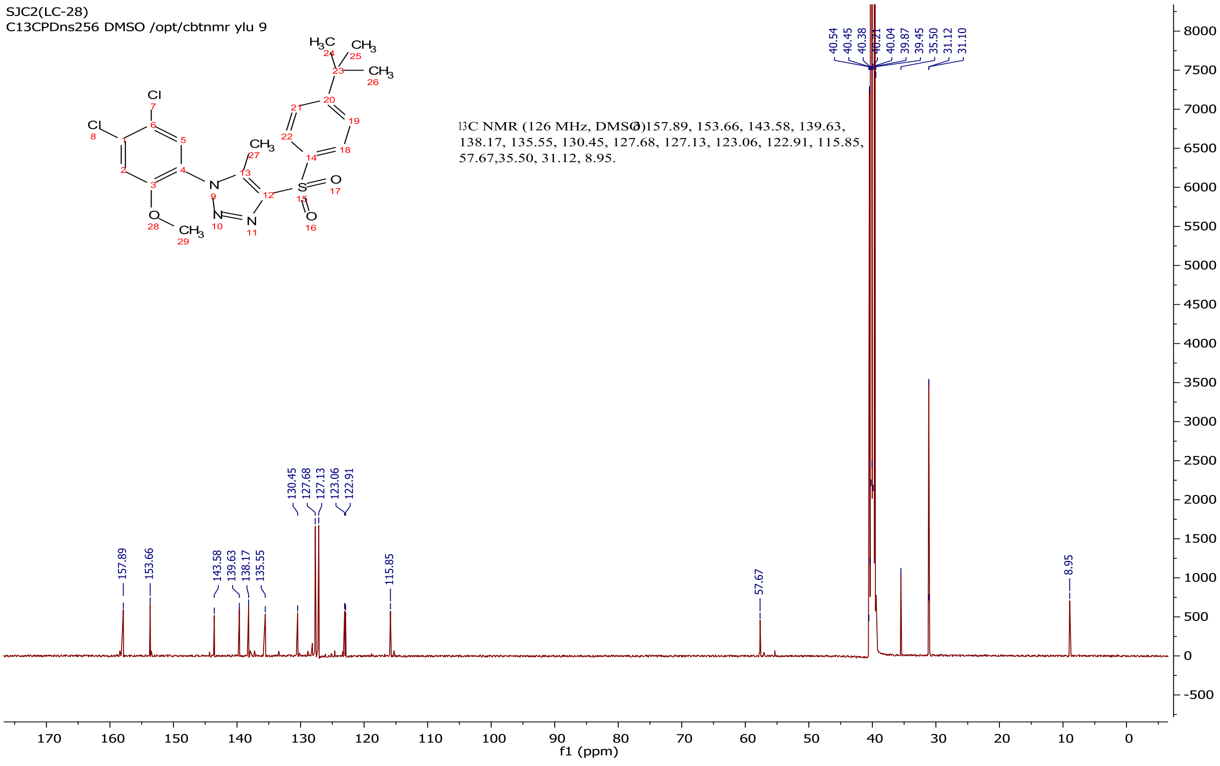

m ^{1}H NMR spectrum of compound **SJC6** (**LC-32**)

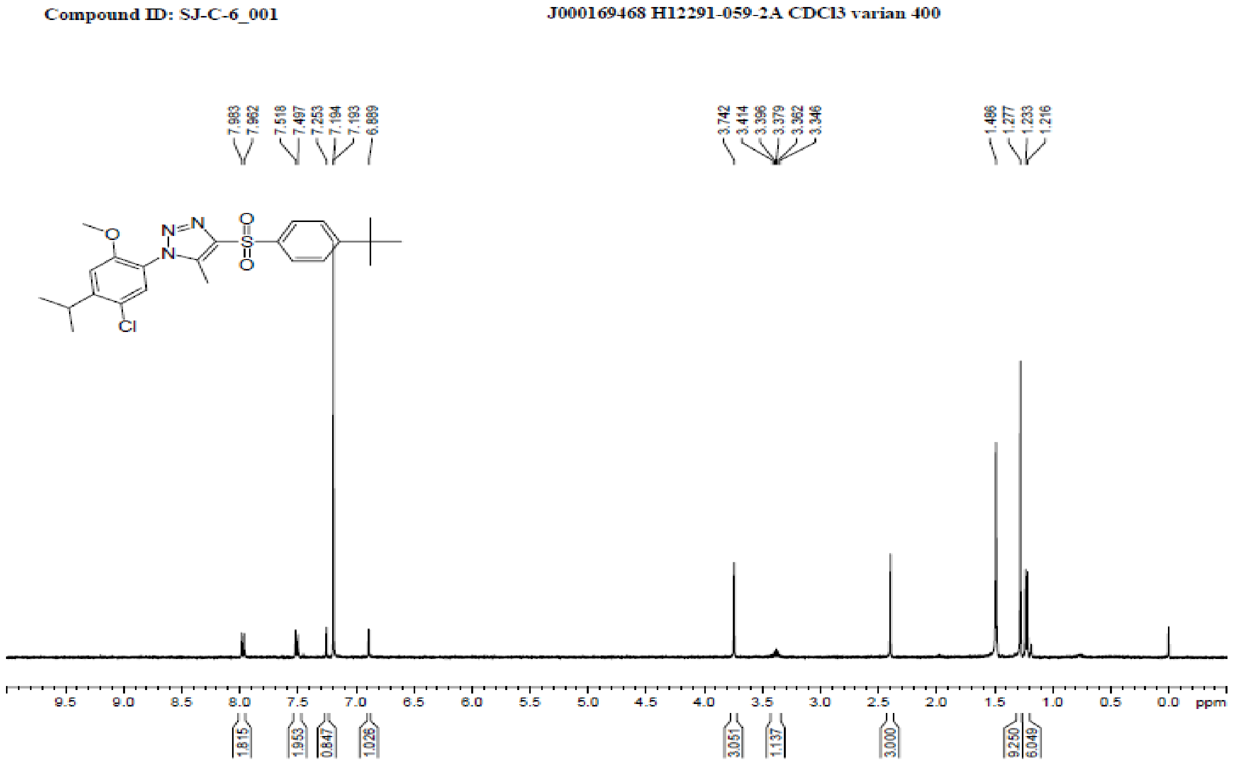

^{13}C NMR spectrum of compound **SJC6** (**LC-32**)

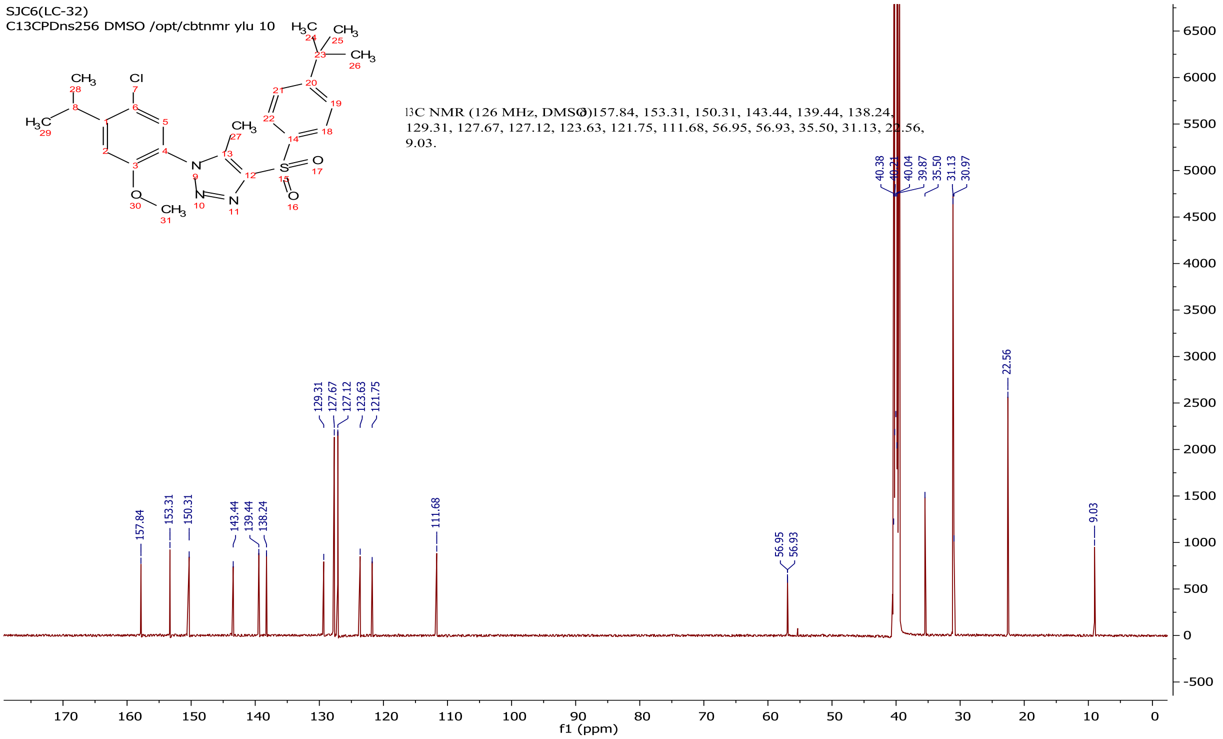

n ^{1}H NMR spectrum of compound **SJC7** (**LC-33**)

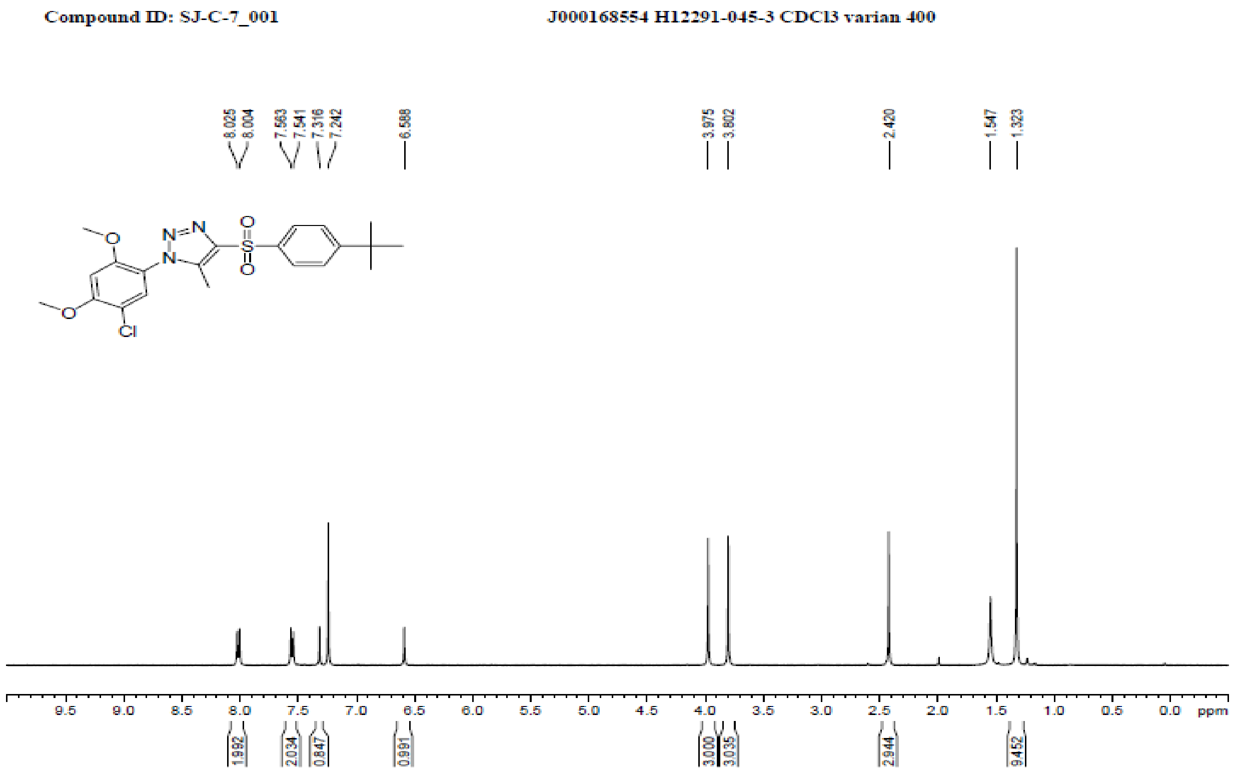

^{13}C NMR spectrum of compound **SJC7** (**LC-33**)

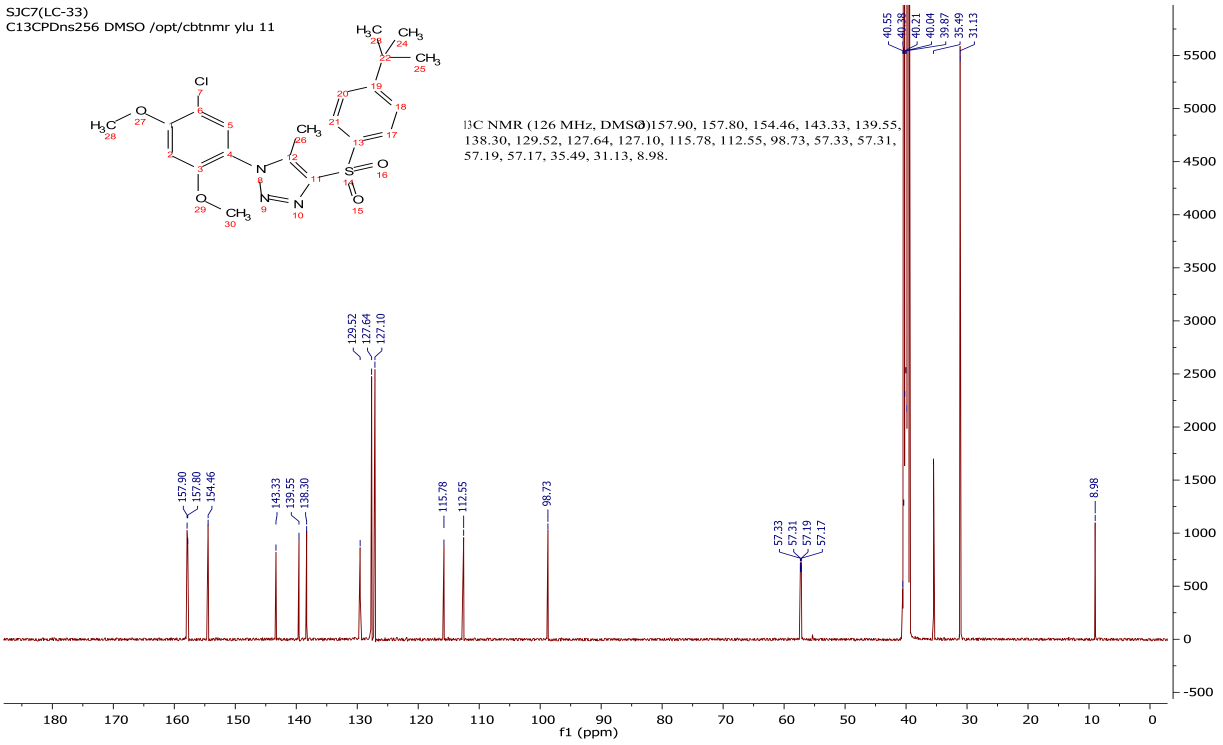

0 ^{1}H NMR spectrum of compound **SJC10** (**LC-36**)

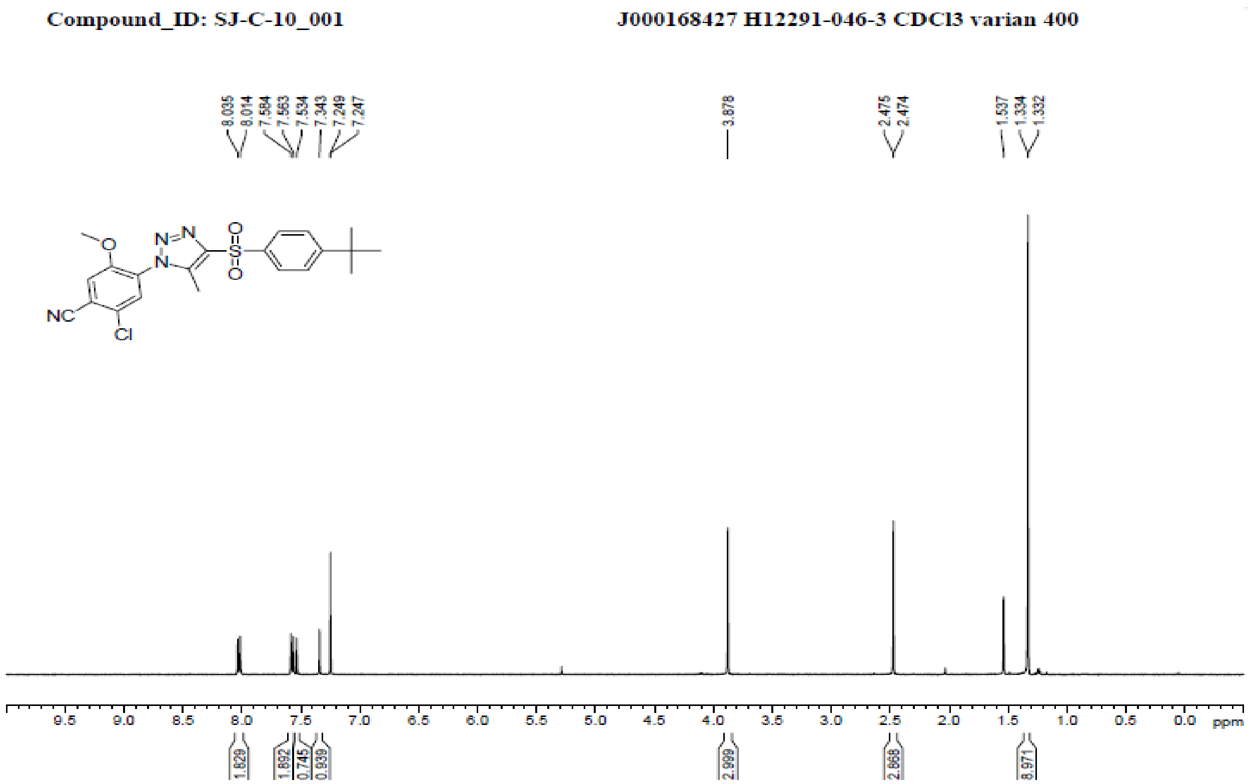

^{13}C NMR spectrum of compound **SJC10** (**LC-36**)

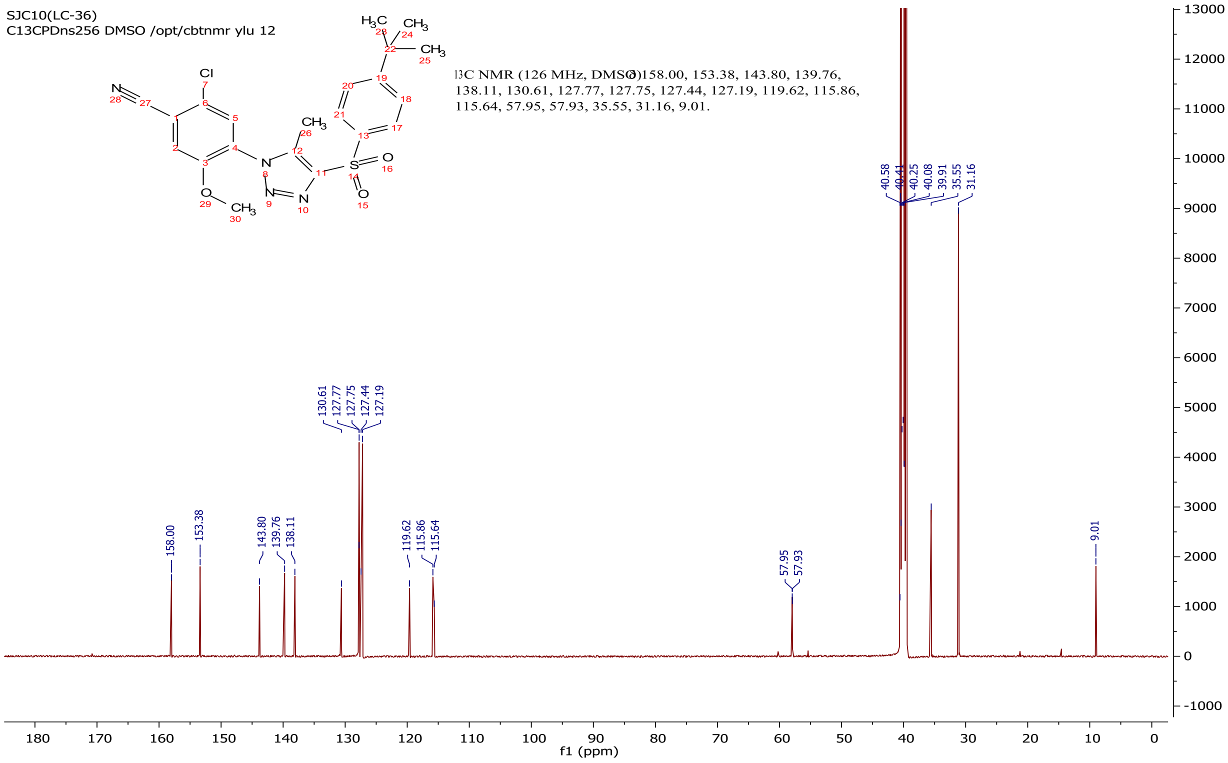

Supplementary Figure 10. ^{1}H and ^{13}C NMR spectra of compounds listed in Supplementary Table 1. ^{1}H (top) and ^{13}C (bottom) NMR spectra for (**a**) SJA1, (**b**) SJA3, (**c**) SJA5, (**d**) SJA6, (**e**) SJA7, (**f**) SJA8, (**g**) SJA9, (**h**) SJA10, (**i**) SJB4, (**j**) SJB7, (**k**) SJB10, (**l**) SJC2, (**m**) SJC6, (**n**) SJC7, and (**o**) SJC10, respectively.

Supplementary Tables

Supplementary Table 1. Activity profile of SPA70 and its analogs.

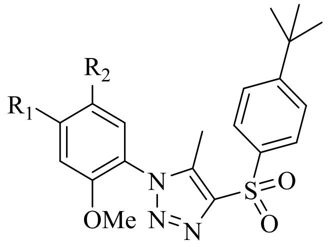

Name	R_1	R_2	IC_{50} (Binding[a]) (µM)	K_i (Binding) (µM)[b]	IC_{50} (Anta[c]) (µM)	EC_{50} (Ag[d]) (µM)
SPA70 (LC-1)	H	MeO	0.54 ± 0.02	0.39 ± 0.01	0.51 ± 0.06	
SJA1 (LC-8)	F	MeO	1.34 ± 0.09	0.96 ± 0.06	1.52 ± 0.13	
SJA3 (LC-10)	Br	MeO	1.53 ± 0.11	1.10 ± 0.08		0.75 ± 0.07
SJA5 (LC-12)	Et	MeO	0.53 ± 0.04	0.38 ± 0.03		0.78 ± 0.11
SJA6 (LC-13)	^{iso}Pro	MeO	0.31 ± 0.02	0.22 ± 0.01		0.84 ± 0.04
SJA7 (LC-14)	MeO	MeO	3.49 ± 0.17	2.51 ± 0.12		7.20 ± 0.28
SJA8 (LC-15)	MeOCO	MeO	4.45 ± 0.14	3.20 ± 0.10		3.80 ± 0.19
SJA9 (LC-16)	CH_3CO	MeO	2.33 ± 0.25	1.68 ± 0.18		1.20 ± 0.16
SJA10 (LC-17)	CN	MeO	2.20 ± 0.13	1.58 ± 0.09		1.30 ± 0.16
SJB4 (LC-21)	Me	Me	0.83 ± 0.09	0.60 ± 0.06	0.99 ± 0.06	
SJB7 (LC-24)	MeO	Me	0.75 ± 0.04	0.54 ± 0.03		0.88 ± 0.07
SJB10 (LC-27)	CN	Me	2.39 ± 0.27	1.72 ± 0.19		1.90 ± 0.16
SJC2 (LC-28)	Cl	Cl	0.56 ± 0.02	0.40 ± 0.01	0.61 ± 0.06	
SJC6 (LC-32)	^{iso}Pro	Cl	0.22 ± 0.03	0.16 ± 0.02		3.40 ± 0.24
SJC7 (LC-33)	MeO	Cl	0.99 ± 0.14	0.71 ± 0.10		1.20 ± 0.09
SJC10 (LC-36)	CN	Cl	0.76 ± 0.04	0.55 ± 0.03		2.40 ± 0.15

[a]Binding: hPXR TR-FRET binding assay; [b]K_i: the binding K_i was calculated using the *Cheng-Prusoff* equation[2] ($K_i = IC_{50}/(1+[S]/K_d$; [S] = 100 nM and K_d = 256 nM for BODIPY FL vindoline, the fluorescent hPXR ligand); [c]Anta: hPXR transactivation assay using the HepG2 stable cell line in antagonistic mode; [d]Ag: hPXR transactivation assay using the HepG2 stable cell line in agonistic mode.

Supplementary Table 2. Activities of reported hPXR antagonists.

Compound	Binding to hPXR LBD	Inhibition of hPXR in cells	Other known activity
ET-743 (ref. 3)	Unknown[a]	IC_{50}: 3 nM	Highly cytotoxic (IC_{50}: 1–100 nM)
PCB 197 (ref. 4)	~50% inhibition @ 25 μM[b]	K_i: 0.6 μM	Potently activates mPXR and rPXR
Ketoconazole[5]	IC_{50}: 74.4 μM	100% inhibition @25 μM[b]	Inhibits mPXR, mCAR, hCAR, FXR, LXRα, and LXRβ
Sulforaphane[6]	K_i: 16 μM	IC_{50}: 12 – 14 μM	Inhibits histone deacetylases; induces phase II enzymes
A-792611 (ref. 7)	Unknown[a]	IC_{50}: 2 μM	Inhibits HIV protease
Coumestrol[8]	K_i: 13 μM	IC_{50}: 12 μM	Potent agonist of ERα and ERβ; inverse agonist of hCAR
SPB06061 (ref. 9)	Unknown[a]	IC_{50}: 5.22 μM	Unknown
Camptothecin[10]	No binding	IC_{50}: 0.58 μM	Inhibitor of topoisomerase I, mPXR, and hCAR; activator of hVDR
FLB-12 (ref. 5,11)	< 10% inhibition @ 2 μM[b]; slightly weaker than ketoconazole	~80% inhibition @ 25 μM[b]	Unknown
Metformin[12]	Unknown[a]	~50% inhibition @ > 1 mM[b]	Inhibits hVDR, hGR, and hCAR
Sesamin[13]	Unknown[a]	~41% inhibition @ 30 μM[b]	Inhibits hCAR and rPXR
Fucoxanthin[14]	Unknown[a]	~60% inhibition @ 5 μM[b]	Inhibits hCAR an rPXR
Isosilybin[15]	~27% inhibition @ 100 μM[b]	IC_{50}: 74.4 μM	Unknown
Resveratrol[16]	Unknown[a]	~60% inhibition @ 25 μM[b]	Inhibits mPXR
Compound 20 (ref. 17)	Unknown[a]	IC_{50}: 11 μM	Downregulates hPXR expression
Allyl isothiocyanate[18]	Unknown[a]	~55% inhibition @ 20 μM[b]	Inhibits hCAR
Diethylstilbestrol[19]	Unknown[a]	IC_{50}:14.6 μM	Activates ERα; inhibits ERRβ

[a]No binding assay performed; [b]No dose response curve or IC_{50} value reported.

Abbreviation: hPXR, human PXR; mPXR, mouse PXR; rPXR, rat PXR; mCAR, mouse CAR; hCAR, human CAR; FXR, farnesoid X receptor; LXRα and LXRβ, liver X receptor α and β; ERα and ERβ, estrogen receptor α and β; hVDR, human vitamin D receptor; hGR, human glucocorticoid receptor; ERRβ, estrogen-related receptor β.

Supplementary Table 3. Primers used for sequencing and RT-PCR.

cDNA to be sequenced	Sequence of primer used for sequencing
hPXR, hPXRM425A, and hPXRF429A	F: 5’-GAGGTCTATATAAGCAGAGCTCTCTGG-3’
	F: 5’-TGGTGGACCAGCTGCAGGAGCAATT-3’
	F: 5’-CATGGCTGACATGTCAACCTACATGTT-3’
	R: 5’-TCAGCGAGCTCTAGCATTTAGGTGA-3’

**Gene**	**Species**	**Amplicon Length**	**Assay ID (for RT-PCR)**	**Vendor**
NR1I2/hPXR	Human	103	Hs01114267_m1	Thermo Fisher Scientific
GAPDH	Human	58	Hs03929097_g1	Thermo Fisher Scientific
18S	Human	187	Hs99999901_s1	Thermo Fisher Scientific
Cyp3a11	Mouse	128	Mm00731567_m1	Thermo Fisher Scientific
Gapdh	Mouse	107	Mm99999915_g1	Thermo Fisher Scientific

Supplementary Table 4. cDNA sequences of codon-optimized hPXR LBD and mSRC-1

cDNA fragment	Nucleotide sequence (5’ to 3’)
hPXR LBD (residues 130–434)	AGCGAGCGTACCGGCACACAGCCTTTAGGTGTGCAGGG<br>CCTGACAGAGGAGCAGCGCATGATGATTCGCGAGCTGA<br>TGGACGCCCAGATGAAAACCTTCGACACCACCTTCAGC<br>CACTTCAAGAATTTTCGCCTGCCGGGCGTGCTGAGTAGT<br>GGTTGCGAACTGCCGGAAAGTCTGCAGGCCCCGAGTCG<br>CGAAGAAGCCGCCAAATGGAGCCAGGTTCGCAAGGAC<br>CTGTGCAGCCTGAAGGTGAGCCTGCAGTTACGCGGCGA<br>AGATGGTAGTGTGTGGAACTACAAGCCGCCGGCAGACA<br>GTGGTGGCAAGGAGATCTTCAGCCTGCTGCCGCACATG<br>GCCGACATGAGCACATACATGTTCAAGGGCATCATTAG<br>CTTTGCCAAGGTTATCAGTTACTTCCGCGACCTGCCGAT<br>CGAGGACCAGATCAGTCTGCTGAAGGGCGCAGCCTTTG<br>AGCTGTGCCAGCTGCGCTTCAACACCGTGTTCAACGCA<br>GAGACCGGTACATGGGAGTGCGGCCGCCTGAGCTACTG<br>CTTAGAGGACACAGCCGGTGGCTTCCAGCAGCTGCTGC<br>TGGAGCCGATGCTGAAGTTCCACTACATGCTGAAGAAG<br>CTGCAACTGCATGAAGAGGAGTACGTTCTGATGCAGGC<br>CATCAGCCTGTTTAGTCCGGACCGCCCGGGCGTTTTACA<br>GCATCGCGTGGTGGACCAGCTGCAGGAGCAGTTCGCCA<br>TCACCCTGAAGAGCTACATTGAGTGCAACCGTCCGCAG<br>CCGGCACATCGCTTCCTGTTCCTGAAGATCATGGCCATG<br>CTGACAGAGCTGCGCAGCATCAACGCACAACACACCCA<br>GCGCCTGCTGCGCATTCAGGACATTCACCCTTTCGCCAC<br>ACCGCTGATGCAGGAGCTGTTCGGCATCACCGGCAGC
mSRC-1 (residues 623–710)	CTGAGCGAGGGCGACAGCAAGTACAGCCAGACCAGCC<br>ACAAGCTGGTGCAGCTGCTGACCACCACCGCCGAACAG<br>CAGCTGCGTCACGCAGACATCGACACCAGCTGCAAGGA<br>CGTGCTGAGCTGCACCGGCACCAGCAGCAGTGCCAGCA<br>GCAACCCTAGCGGTGGCACCTGCCCGAGCAGTCACAGC<br>AGCCTGACCGAGCGCCACAAGATCCTGCACCGCCTGCT<br>GCAGGAAGGTAGCCCGAGCGACATCACCACCCTGAGC

Supplementary Methods

General chemical preparation procedures. All reactions were carried out in flame-dried glassware under argon or nitrogen. Flash column chromatography was performed by using Sigma-Aldrich silica gel 60 (200-400 mesh) (Sigma, St. Louis, MO). All reactions were monitored by performing thin-layer chromatography (TLC) on pre-coated plates (silica gel HLF). The reactions, purities, or identities of final compounds were monitored or determined by TLC or a Waters Acquity UPLC MS system (Waters Corporation, Milford, MA). The purifications of reaction products were performed by using a Dionex APS 3000 dual purification/analytical LC/PDA/MS system (Dionex Corporation, Sunnyvale, CA). High-resolution mass spectra were determined by using a Waters Acquity UPLC system under Xevo G2Q-TOF ESI in positive resolution mode. All ^{1}H NMR spectra were recorded on a Bruker ULTRASHIELD 400 plus NMR spectrometer (Bruker BioSpin Corporation, Billerica, CA) and all ^{13}C NMR spectra were recorded on a Bruker Ascend 126 MHz Fourier transform (FT) NMR spectrometer (Bruker BioSpin Corporation) at room temperature. The chemical shift values are expressed in parts per million (ppm) relative to tetramethylsilane as the internal standard. Coupling constants (*J*) are reported in hertz (Hz).

Chemical synthesis. SPA70 analogs SJA1, SJA3, SJA5, SJA6, SJA7, SJA8, SJA9, SJA10, SJB4, SJB7, SJB10, SJC2, SJC6, SJC7 and SJC10 were prepared using a similar protocol described for SJB7.

4-((4-(tert-butyl)phenyl)sulfonyl)-1-(4-fluoro-2,5-dimethoxyphenyl)-5-methyl-1H-1,2,3-triazole (SJA1). ^{1}H NMR ($CDCl_3$, 400 MHz): δ (ppm) δ 8.03 (d, 2H, J = 8.4 Hz), 7.56 (d, 2H, J = 8.4 Hz), 6.94 (d, 1H, J = 8.4 Hz), 6.86 (d, 1H, J =12.4 Hz), 3.82 (s, 3H), 3.73 (s, 3H), 2.43 (s, 3H), 1.32 (s, 9H). ^{13}C NMR (126 MHz, DMSO-d_6) δ (ppm) 157.42, 154.07, 152.09, 148.09, 148.01, 143.03, 140.96, 140.87, 138.95, 137.84, 127.24, 126.71, 118.05, 118.02, 114.60, 114.57, 102.66, 102.48,

56.90, 56.89, 56.83, 56.81, 35.07, 30.70, 8.54. ESI-TOF HRMS: *m/z* 434.1546 ($C_{21}H_{24}FN_3O_4S + H^+$ requires 434.1550).

1-(4-bromo-2,5-dimethoxyphenyl)-4-((4-(tert-butyl)phenyl)sulfonyl)-5-methyl-1H-1,2,3-triazole (SJA3). ^{1}H NMR ($CDCl_3$, 400MHz): δ (ppm) 8.56 (d, 2H, J = 8.8 Hz), 8.03 (d, 2H, J = 8.8 Hz), 7.29 (s, 1H), 6.87 (s, 1H), 3.82 (s, 3H), 3.75 (s, 3H), 2.45 (s, 3H), 1.32 (s, 9H). ^{13}C NMR (126 MHz, $CDCl_3$) δ (ppm) 157.712, 150.435, 147.781, 144.405, 138.529, 137.945, 127.796, 126.330, 122.750, 117.770, 115.065, 112.032, 57.012, 56.655, 35.291, 31.063, 9.021. ESI-TOF HRMS: m/z 494.0747 (C21H24BrN3O4S + H+ requires 494.0749).

4-((4-(tert-butyl)phenyl)sulfonyl)-1-(4-ethyl-2,5-dimethoxyphenyl)-5-methyl-1H-1,2,3-triazole (SJA5). ^{1}H NMR ($CDCl_3$, 400MHz): δ (ppm) 7.98 (d, 2H, J = 8.8 Hz), 7.50 (d, 2H, J = 8.8 Hz), 6.81 (s, 1H), 6.70 (s, 1H), 3.69 (s, 3H), 3.67 (s, 3H), 2.65~2.59 (m, 2H), 2.39 (s, 3H), 1.27 (s, 9H), 1.14 (t, 3H, J = 7.6 Hz). ^{13}C NMR (126 MHz, DMSO-d_6) δ (ppm) 157.85, 151.17, 147.75, 143.42, 139.20, 138.37, 136.70, 127.69, 127.17, 120.71, 114.22, 111.57, 56.80, 56.78, 56.63, 56.61, 35.53, 31.17, 23.60, 14.50, 9.09. ESI-TOF HRMS: *m/z* 444.1945 ($C_{23}H_{29}N_3O_4S + H^+$ requires 444.1957).

4-((4-(tert-butyl)phenyl)sulfonyl)-1-(4-isopropyl-2,5-dimethoxyphenyl)-5-methyl-1H-1,2,3-triazole (SJA6). ^{1}H NMR ($CDCl_3$, 400MHz): δ (ppm) 8.03 (d, 2H, J = 8.4 Hz), 7.55 (d, 2H, J = 8.4 Hz), 6.90 (s, 1H), 6.75 (s, 1H), 3.74 (s, 3H), 3.72 (s, 3H), 3.38~3.31 (m, 1H), 2.45 (s, 3H), 1.32 (s, 9H), 1.22 (d, 6H, J = 6.8 Hz). ^{13}C NMR (126 MHz, DMSO-d_6) δ (ppm) 157.38, 150.16, 147.51, 142.95, 140.60, 138.73, 137.90, 127.23, 126.70, 120.18, 111.36, 110.94, 56.36, 56.34, 56.28, 56.26, 35.07,

30.70, 26.85, 22.26, 8.65. ESI-TOF HRMS: *m/z* 458.2109 ($C_{24}H_{31}N_3O_4S + H^+$ requires 458.2113).

4-((4-(tert-butyl)phenyl)sulfonyl)-5-methyl-1-(2,4,5-trimethoxyphenyl)-1H-1,2,3-triazole (SJA7). ^{1}H NMR ($CDCl_3$, 400MHz): δ (ppm) 7.98 (d, 2H, J = 8.4 Hz), 7.50 (d, 2H, J = 8.4 Hz), 6.75 (s, 1H), 6.55 (s, 1H), 3.90 (s, 3H), 3.75 (s, 3H), 3.69 (s, 3H), 2.38 (s, 3H), 1.27 (s, 9H). ^{13}C NMR (126 MHz, $CDCl_3$) δ (ppm) 157.540, 151.902, 148.419, 144.045, 143.281, 138.671, 138.116, 127.720, 126.252, 115.010, 111.554, 97.536, 56.610, 56.566, 56.352, 35.241, 31.037, 8.975. ESI-TOF HRMS: *m/z* 446.1745 ($C_{22}H_{27}N_3O_5S + H^+$ requires 446.1749).

Methyl-4-(4-((4-(tert-butyl)phenyl)sulfonyl)-5-methyl-1H-1,2,3-triazol-1-yl)-2,5-dimethoxybenzoate (SJA8). ^{1}H NMR ($CDCl_3$, 400MHz): δ (ppm) .98 (d, 2H, J = 8.8 Hz), 7.51 (d, 2H, J = 8.8 Hz), 7.44 (s, 1H), 6.92 (s, 1H), 3.87 (s, 3H), 3.78 (s, 3H), 3.73 (s, 3H), 2.41 (s, 3H), 1.27 (s, 9H). ^{13}C NMR (126 MHz, DMSO-d_6) δ (ppm) 165.40, 157.45, 151.54, 146.93, 143.17, 138.86, 137.77, 127.25, 126.72, 125.57, 123.49, 114.37, 113.78, 56.78, 56.57, 52.43, 35.06, 30.68, 8.56. ESI-TOF HRMS: *m/z* 474.1687 ($C_{23}H_{27}N_3O_6S + H^+$ requires 474.1699).

1-(4-(4-((4-(tert-butyl)phenyl)sulfonyl)-5-methyl-1H-1,2,3-triazol-1-yl)-2,5-dimethoxyphenyl)ethan-1-one (SAJ9). ^{1}H NMR ($CDCl_3$, 400MHz): δ (ppm) 7.99 (d, 2H, J = 8.8 Hz), 7.52 (d, 2H, J = 8.8 Hz), 7.41 (s, 1H), 6.93 (s, 1H), 3.81 (s, 3H), 3.74 (s, 3H), 2.60 (s, 3H), 2.42 (s, 3H), 1.28 (s, 9H). ^{13}C NMR (126 MHz, $CDCl_3$) δ (ppm) 198.288, 157.763, 152.973, 147.278, 144.500, 138.558, 137.894, 130.457, 127.789, 126.987, 126.338, 113.884, 112.572, 56.414, 56.371, 35.291, 31.908, 31.055, 9.065. ESI-TOF HRMS: *m/z* 458.1739 ($C_{23}H_{27}N_3O_5S + H^+$ requires 458.1749).

4-(4-((4-(tert-butyl)phenyl)sulfonyl)-5-methyl-1H-1,2,3-triazol-1-yl)-2,5-dimethoxybenzonitrile (SJA10). ^{1}H NMR ($CDCl_3$, 400MHz): δ (ppm) 8.63 (d, 2H, J = 8.8 Hz), 8.09 (d, 2H, J = 8.8 Hz), 7.31 (s, 1H), 7.05 (s, 1H), 3.94 (s, 3H), 3.85 (s, 3H), 2.53 (s, 3H), 1.39 (s, 9H). ^{13}C NMR (126 MHz, DMSO-d_6) δ (ppm) 157.52, 155.08, 147.42, 143.29, 138.98, 137.71, 127.45, 127.28, 126.75, 117.79, 115.35, 113.55, 103.04, 57.21, 57.19, 57.03, 57.01, 35.08, 30.69, 8.56. ESI-TOF HRMS: m/z 441.1588 ($C_{22}H_{24}N_4O_4S + H^+$ requires 441.1596).

4-((4-(tert-butyl)phenyl)sulfonyl)-1-(2-methoxy-4,5-dimethylphenyl)-5-methyl-1H-1,2,3-triazole (SJB4). ^{1}H NMR ($CDCl_3$, 400MHz): δ (ppm) 8.02 (d, 2H, J = 8.8 Hz), 7.55 (d, 2H, J = 8.8 Hz), 7.03 (s, 1H), 6.82 (s, 1H), 3.73 (s, 3H), 2.41 (s, 3H), 2.31 (s, 3H), 2.19 (s, 3H), 1.32 (s, 9H). ^{13}C NMR (126 MHz, DMSO-d_6) δ (ppm) 157.35, 151.25, 142.99, 141.38, 138.64, 137.91, 128.91, 128.79, 127.17, 126.68, 120.00, 113.99, 56.03, 56.02, 35.06, 30.70, 19.77, 18.11, 8.57. ESI-TOF HRMS: m/z 414.1859 ($C_{22}H_{27}N_3O_3S + H^+$ requires 414.1851).

4-(4-((4-(tert-butyl)phenyl)sulfonyl)-5-methyl-1H-1,2,3-triazol-1-yl)-5-methoxy-2-methylbenzonitrile (SJB10). ^{1}H NMR ($CDCl_3$, 400MHz): δ (ppm) 7.97 (d, 2H, J = 8.4 Hz), 7.51 (d, 2H, J = 8.4 Hz), 7.25 (s, 1H), 7.19 (s, 1H), 3.77 (s, 3H), 2.46 (s, 3H), 2.39 (s, 3H), 1.27 (s, 9H). ^{13}C NMR (126 MHz, DMSO-d_6) δ (ppm) 157.49, 151.67, 143.33, 138.95, 137.71, 134.55, 130.53, 127.24, 126.72, 126.35, 116.96, 116.94, 115.17, 56.94, 56.92, 35.07, 30.69, 18.81, 8.56. ESI-TOF HRMS: m/z 425.1649 ($C_{22}H_{24}N_4O_3S + H^+$ requires 425.1647).

4-((4-(tert-butyl)phenyl)sulfonyl)-1-(4,5-dichloro-2-methoxyphenyl)-5-methyl-1H-1,2,3-triazole (SJC2). ^{1}H NMR ($CDCl_3$, 400MHz): δ (ppm) 7.96 (d, 2H, J = 8.4 Hz), 7.51 (d, 2H, J = 8.4 Hz), 7.38 (s, 1H), 7.11 (s, 1H), 3.75 (s, 3H), 2.39 (s, 3H), 1.27 (s, 9H). ^{13}C NMR (126 MHz, DMSO-d_6) δ (ppm) 157.89, 153.66, 143.58, 139.63, 138.17, 135.55, 130.45, 127.68, 127.13, 123.06, 122.91, 115.85, 57.67, 35.50, 31.12, 8.95. ESI-TOF HRMS: m/z 454.0757 (C20H21Cl2N3O3S + H+ requires 454.0759).

4-((4-(tert-butyl)phenyl)sulfonyl)-1-(5-chloro-4-isopropyl-2-methoxyphenyl)-5-methyl-1H-1,2,3-triazole (SJC6). ^{1}H NMR ($CDCl_3$, 400MHz): δ (ppm) 7.97 (d, 2H, J = 8.4 Hz), 7.50 (d, 2H, J = 8.4 Hz), 7.25 (s, 1H), 6.83 (s, 1H), 3.70 (s, 3H), 3.13~3.06 (m, 1H), 2.38 (s, 3H), 2.21 (s, 3H), 1.27 (s, 9H), 1.19 (d, 6H, J = 6.8 Hz). ^{13}C NMR (126 MHz, DMSO-d_6) δ (ppm) 157.84, 153.31, 150.31, 143.44, 139.44, 138.24, 129.31, 127.67, 127.12, 123.63, 121.75, 111.68, 56.95, 56.93, 35.50, 31.13, 22.56, 9.03. ESI-TOF HRMS: *m*/*z* 462.1609 ($C_{23}H_{28}ClN_3O_3S$ + H^+ requires 462.1618).

4-((4-(tert-butyl)phenyl)sulfonyl)-1-(5-chloro-2,4-dimethoxyphenyl)-5-methyl-1H-1,2,3-triazole (SJC7). ^{1}H NMR ($CDCl_3$, 400MHz): δ (ppm) 8.01 (d, 2H, J = 8.4 Hz), 7.55 (d, 2H, J = 8.4 Hz), 7.31 (s, 1H), 6.58 (s, 1H), 3.97 (s, 3H), 3.80 (s, 3H), 2.42 (s, 3H), 1.32 (s, 9H). ^{13}C NMR (126 MHz, DMSO-d_6) δ (ppm) 157.90, 157.80, 154.46, 143.33, 139.55, 138.30, 129.52, 127.64, 127.10, 115.78, 112.55, 98.73, 57.33, 57.31, 57.19, 57.17, 35.49, 31.13, 8.98. ESI-TOF HRMS: *m*/*z* 450.1239 ($C_{21}H_{24}ClN_3O_4S$ + H^+ requires 450.1254).

4-(4-((4-(tert-butyl)phenyl)sulfonyl)-5-methyl-1H-1,2,3-triazol-1-yl)-2-chloro-5-methoxybenzonitrile (SJC10). ^{1}H NMR ($CDCl_3$, 400MHz): δ (ppm) 8.02 (d, 2H, J = 8.4 Hz), 7.57 (d, 2H, J = 8.4 Hz), 7.53 (s, 1H), 7.34 (s, 1H), 3.87 (s, 3H), 2.47 (s, 3H), 1.33 (s, 9H). ^{13}C NMR (126

MHz, DMSO-d_6) δ (ppm) 158.00, 153.38, 143.80, 139.76, 138.11, 130.61, 127.77, 127.75, 127.44, 127.19, 119.62, 115.86, 115.64, 57.95, 57.93, 35.55, 31.16, 9.01. ESI-TOF HRMS: *m/z* 445.1091 ($C_{21}H_{21}N_4O_3S + H^+$ requires 445.1101).

Kinase inhibition profiling assays. The kinase inhibition profiling assay (KINOME*scan*) was performed by Ambit Biosciences, now part of DiscoveryX (Fremont, CA) in accordance with the published protocol[20, 21]. SPA70 was first tested at 10 µM. If the inhibition of a kinase was greater than 50%, the K_d was determined by further testing with SPA70 (in 11-point 3-fold serial dilutions) starting from 30 µM.

Supplementary References

1. Keppel,T.R. & Weis,D.D. Mapping residual structure in intrinsically disordered proteins at residue resolution using millisecond hydrogen/deuterium exchange and residue averaging. *J. Am. Soc. Mass Spectrom.* **26**, 547-554 (2015).

2. Cheng,Y. & Prusoff,W.H. Relationship between the inhibition constant (K1) and the concentration of inhibitor which causes 50 per cent inhibition (I50) of an enzymatic reaction. *Biochem. Pharmacol.* **22**, 3099-3108 (1973).

3. Synold,T.W., Dussault,I., & Forman,B.M. The orphan nuclear receptor SXR coordinately regulates drug metabolism and efflux. *Nat Med* **7**, 584-590 (2001).

4. Tabb,M.M. *et al.* Highly chlorinated PCBs inhibit the human xenobiotic response mediated by the steroid and xenobiotic receptor (SXR). *Environ Health Persp* **112**, 163-169 (2004).

5. Huang,H. *et al.* Inhibition of drug metabolism by blocking the activation of nuclear receptors by ketoconazole. *Oncogene* **26**, 258-268 (2007).

6. Zhou,C. *et al.* The dietary isothiocyanate sulforaphane is an antagonist of the human steroid and xenobiotic nuclear receptor. *Mol Pharmacol.* **71**, 220-229 (2007).

7. Healan-Greenberg,C. *et al.* A human immunodeficiency virus protease inhibitor is a novel functional inhibitor of human pregnane X receptor. *Drug Metab Dispos.* **36**, 500-507 (2008).

8. Wang,H.W. *et al.* The phytoestrogen Coumestrol is a naturally occurring antagonist of the human pregnane x receptor. *Mol Endocrinol* **22**, 838-857 (2008).

9. Ekins,S. *et al.* Computational discovery of novel low micromolar human pregnane X receptor antagonists. *Mol Pharmacol* **74**, 662-672 (2008).

10. Chen,Y.K., Tang,Y., Robbins,G.T., & Nie,D.T. Camptothecin Attenuates Cytochrome P450 3A4 Induction by Blocking the Activation of Human Pregnane X Receptor. *J Pharmacol Exp Ther* **334**, 999-1008 (2010).

11. Venkatesh,M. *et al.* In vivo and in vitro characterization of a first-in-class novel azole analog that targets pregnane X receptor activation. *Mol. Pharmacol.* **80**, 124-135 (2011).

12. Krausova,L. *et al.* Metformin suppresses pregnane X receptor (PXR)-regulated transactivation of CYP3A4 gene. *Biochem Pharmacol* **82**, 1771-1780 (2011).

13. Lim,Y.P. *et al.* Sesamin: A Naturally Occurring Lignan Inhibits CYP3A4 by Antagonizing the Pregnane X Receptor Activation. *Evid-Based Compl Alt*(2012).

14. Liu,C.L., Lim,Y.P., & Hu,M.L. Fucoxanthin attenuates rifampin-induced cytochrome P450 3A4 (CYP3A4) and multiple drug resistance 1 (MDR1) gene expression through pregnane X receptor (PXR)-mediated pathways in human hepatoma HepG2 and colon adenocarcinoma LS174T cells. *Mar. Drugs* **10**, 242-257 (2012).

15. Mooiman,K.D. *et al.* Milk thistle's active components silybin and isosilybin: novel inhibitors of PXR-mediated CYP3A4 induction. *Drug Metab Dispos.* **41**, 1494-1504 (2013).

16. Deng,R. *et al.* Resveratrol suppresses the inducible expression of CYP3A4 through the pregnane X receptor. *J. Pharmacol. Sci.* **126**, 146-154 (2014).

17. Hodnik,Z. *et al.* Bazedoxifene-scaffold-based mimetics of solomonsterols A and B as novel pregnane X receptor antagonists. *J. Med. Chem.* **57**, 4819-4833 (2014).

18. Lim,Y.P. *et al.* Allyl isothiocyanate (AITC) inhibits pregnane X receptor (PXR) and constitutive androstane receptor (CAR) activation and protects against acetaminophen- and amiodarone-induced cytotoxicity. *Arch. Toxicol.* **89**, 57-72 (2015).

19. Hodnik,Z. *et al.* Diethylstilbestrol-scaffold-based pregnane X receptor modulators. *Eur. J. Med. Chem.* **103**, 551-562 (2015).

20. Fabian,M.A. *et al.* A small molecule-kinase interaction map for clinical kinase inhibitors. *Nat. Biotechnol.* **23**, 329-336 (2005).

21. Karaman,M.W. *et al.* A quantitative analysis of kinase inhibitor selectivity. *Nat. Biotechnol.* **26**, 127-132 (2008).
