# Supplementary material for: SPA70 is a potent antagonist of human pregnane X receptor
Source: Nat Commun. 2017 Sep 29;8:741. doi: 10.1038/s41467-017-00780-5 (PMC5622171; doi:10.1038/s41467-017-00780-5)
Supplement: Supplementary file 2 — Supplementary Description [file 41467_2017_780_MOESM2_ESM.pdf]

Description of Additional Supplementary Files

File Name: Supplementary Data 1
Description: Selectivity profiling of SPA70 against a panel of 384 kinases.
